# Epigenetic variation associated with responses to different habitats in the context of genetic divergence in *Phragmites australis*

**DOI:** 10.1002/ece3.7954

**Published:** 2021-07-30

**Authors:** Tian Qiu, Zhiyuan Liu, Yunfei Yang, Bao Liu

**Affiliations:** ^1^ School of Life Sciences Changchun Normal University Changchun China; ^2^ Institute of Grassland Science Key Laboratory of Vegetation Ecology Ministry of Education Northeast Normal University Changchun China; ^3^ Key Laboratory of Molecular Epigenetics Ministry of Education Northeast Normal University Changchun China; ^4^ College of Computer Science and Technology Changchun University Changchun China

**Keywords:** cosmopolitan species, DNA methylation, epigenetic differentiation, habitats, partial Mantel

## Abstract

The mechanisms underlying heritable phenotypic divergence associated with adaptation in response to environmental stresses may involve both genetic and epigenetic variations. Several prior studies have revealed even higher levels of epigenetic variation than genetic variation. However, few population‐level studies have explored the effects of epigenetic variation on species with high levels of genetic diversity distributed across different habitats. Using AFLP and methylation‐sensitive AFLP markers, we tested the hypothesis that epigenetic variation may contribute to differences in plants occupying different habitats when genetic variation alone cannot fully explain adaptation. As a cosmopolitan invasive species, *Phragmites australis* (common reed) together with high genetic diversity and remarkable adaptability has been suggested as a model for responses to global change and indicators of environmental fluctuations. We found high levels of genetic and epigenetic diversity and significant genetic/epigenetic structure within each of 12 studied populations sampled from four natural habitats of *P*. *australis*. Possible adaptive epigenetic variation was suggested by significant correlations between DNA methylation‐based epigenetic differentiation and adaptive genetic divergence in populations across the habitats. Meanwhile, various AMOVAs indicated that some epigenetic differences may respond to various local habitats. A partial Mantel test was used to tease out the correlations between genetic/epigenetic variation and habitat after controlling for the correlation between genetic and epigenetic variations. We found that epigenetic diversity was affected mostly by soil nutrient availability, suggesting that at least some epigenetic differentiation occurred independently of genetic variation. We also found stronger correlations between epigenetic variation and phenotypic traits than between genetic variation and such traits. Overall, our findings indicate that genetically based differentiation correlates with heterogeneous habitats, while epigenetic variation plays an important role in ecological differentiation in natural populations of *P*. *australis*. In addition, our results suggest that when assessing global change responses of plant species, intraspecific variation needs to be considered.

## Graphical abstract

Various morphological traits of native *Phragmites australis* differ significantly among four habitat types with markedly different edaphic characteristics, including moisture, pH, and ions in the Songnen Prairie in Northeast China. Our findings indicate that genetically based differentiation correlates with heterogeneous habitats, while epigenetic variation plays an important role in ecological differentiation in natural populations of *P. australis*.

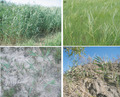

## 1 INTRODUCTION

Ecological theory predicts that adaptation to contrasting environmental stresses occurs when populations harbor adaptive phenotypic variation that matches the respective environments (Peláez et al., 2020; Robertson et al., 2017). Among the factors influencing adaptive phenotypic divergence, three prominent forces are divergent natural selection, gene flow, and phenotypic plasticity, which interact to propel divergence (Card et al., 2018; Crispo, 2008; Hendry et al., 2002). Phenotypic response to the environment is modulated at the molecular level. Classical population genetics has been informative regarding how natural selection can result in associations of genetic structure or candidate genes with different environments despite high frequency of gene flow (Linhart & Grant, 1996; Mooney et al., 2010; Yang et al., 2016). However, the molecular underpinnings of responses to complex environmental conditions across a diversity of taxa, which often show either low levels of genetic differences or no association with habitat, are not well understood (Foust et al., 2016; Geng et al., 2007; Richards et al., 2012). Besides genetic variation, evidence that epigenetic mechanisms are important due to their effect on heritable phenotypic variations is accumulating (Mounger et al., 2021). The most studied epigenetic mechanism in ecology is DNA methylation (Richards et al., 2017).

DNA methylation variation can be directly induced by specific environmental conditions and at least some of which can be transgenerationally inheritable (Shi et al., 2019). Moreover, DNA methylation variants may occur at faster and higher rates than do DNA sequence changes to allow phenotypic changes that enable initial and transgenerational adaptation (Banta & Richards, 2018; Paun et al., 2010). Empirical evidence showed that epigenetic variations can persist even after a particular stress is relieved or intermittently recurring, and after which, genetic changes can occur in a relying manner to sustain protracted organismal adaptation (Banta & Richards, 2018; Crispo, 2008; Robertson et al., 2017). Also, immediate phenotypic plasticity itself can be mediated by DNA methylation modification (Richards, 2011; Verhoeven et al., 2016). Many studies have correlated methylation variation with local environmental conditions, including biotic and abiotic factors. As such, DNA methylation can provide a mechanism of rapid adaptation under stressful conditions and hence impact evolution (Angers et al., 2010; Liu et al., 2018).

Necessarily, the occurrence of epigenetic variation is superimposed on genetic context, and the two are intermingled with each other. Detecting the relationships between genetic and epigenetic systems is considered as one of the most important ecological issues to be addressed by biologists (Mónica et al., 2020; Richards et al., 2017). Epigenetic variation in relation to genetic variation can be categorized as “obligate” or “facilitated,” while environment‐induced epigenetic variation is referred to as “pure” (Foust et al., 2016). A study of the violet *Viola cazorlensis* uncovered evidence of a significant correlation between epigenetic differentiation and adaptive genetic divergence (Herrera & Bazaga, 2010). Each component of phenotypic variance measured in quantitative genetic studies can be influenced by both genetic and epigenetic underpinnings (Banta & Richards, 2018). Although functional DNA methylation polymorphisms are suggested to be largely correlated with genetic polymorphisms, we still have limited data about the extent to which there are independent effects and their functions. Although it is difficult to disentangle genetic and epigenetic variations in wild populations, evaluating epigenetic processes in real‐world contexts in the presence of genetic variation is of factual importance.

**FIGURE 1:**
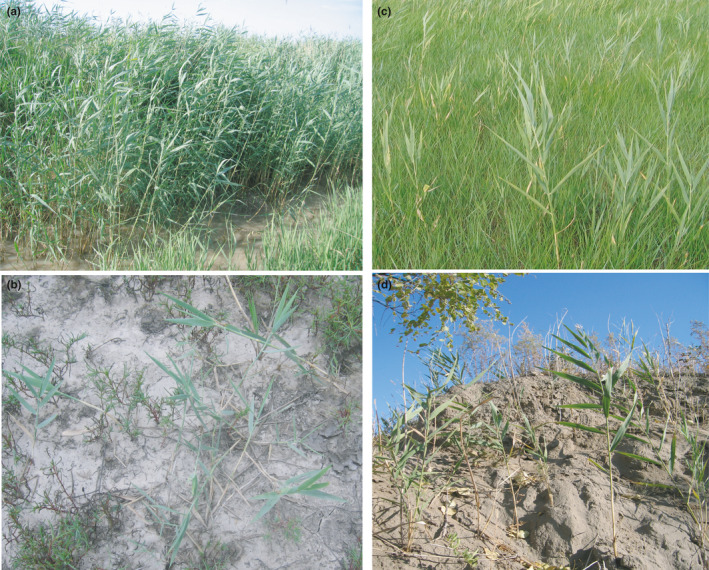
*Phragmites australis* of 4 habitats in the study area at the Pasture Ecology Research Station of Northeast Normal University. (a) Habitat 1; (b) Habitat 2; (c) Habitat 3; (d) Habitat 4

*Phragmites australis* (common reed) is a cosmopolitan grass with high intraspecific diversity and high genetic and morphological variations; it therefore can offer valuable insights into plant responses to global change. Changes of *P. australis* may have strong socioeconomic impacts that indicate and provide feedback on changing environmental conditions. *P. australis* can reproduce both sexually and asexually and is an invasive plant in North America, which makes it a particularly good model for understanding adaptation and invasion (Eller et al., 2017; Holmes et al., 2016). The Songnen Prairie is one of the three largest soda saline–alkaline areas in the world (Yu et al., 2014). Although the area is an important base for agriculture and stockbreeding, land salinization and desertification have become serious due to intensified anthropogenic activities over the last >100 years and have led to a mosaic of habitat patches (Bian et al., 2008; Dianfa et al., 2000). Various morphological traits of native *P. australis* differ significantly among four habitat types with markedly different edaphic characteristics, including moisture, pH, and ions (Figure 1, Table S1 and Figures S1–S3). Our previous studies revealed that height, total biomass, stem biomass, and leaf sheath biomass of *P. australis* differed significantly among these habitats. The stem and leaf fraction, rhizome length, and photosynthesis parameters also differed greatly (Yang & Lang, 1998; Yang et al., 2003).

In this study, we simultaneously examined genetic and DNA methylation variations in the four habitats of *P. australis*. We aimed to determine whether epigenetic variation contributes to wide environmental tolerance by assessing correlations and autonomy within each genetic context. We addressed this issue in four steps. First, we determined the extent and structure of genetic and epigenetic variations within and among habitats possibly shaped by the saline–alkaline edaphic environment, using classic population genetic approaches. Genetic differentiation can be detected over distances of even within a few meters when selection is sufficiently strong to overcome the homogenizing effect of gene flow (Linhart & Grant, 1996). Otherwise, organisms with very high levels of phenotypic plasticity, such as the invasive species of *Fallopia*, *Alternanthera philoxeroides*, and *Bromus tectorum*, often show low levels of genetic variation (Arnesen et al., 2017; Geng et al., 2006; Richards et al., 2012). Second, assuming the existence of genetic differentiation, we searched for correlations linking between‐habitat epigenetic differentiation with adaptive genetic divergence. Such correlations will be indicative of epigenetic differentiation being directly or indirectly driven by selection in the context of a genetically coherent, panmictic unit connected by extensive gene flow (Herrera & Bazaga, 2010). Third, given high levels of both genetic and epigenetic differentiation, it is difficult to determine whether there is a relationship between epigenetic variation and habitat that is not explained by genetic variation. However, adapting statistical tests offer a way to control for the genetic component of responses. We used the Mantel test to detect the relationship between genetic and epigenetic variations. Then, the partial Mantel test was used to examine the respective correlations of genetic and epigenetic variations with habitat by controlling for the correlation between genetic and epigenetic variations (Foust et al., 2016). Finally, we compared the correlation of both types of variation with phenotypic variation. Together, these steps tested the hypothesis that epigenetic mechanism in the form of DNA methylation repatterning may respond to habitat differences that are not fully explained by genetic variation.

## 2 MATERIALS AND METHODS

### 2.1 Study area and habitat sampling

The Songnen Prairie in Northeast China, surrounded by mountains to the east, west, and north and an uplift belt to the south, belongs to an agro‐pastoral interlocking zone with a total area of 1.7 × 10^5^ km^2^ (Zhang & Wang, 2001). The evolutionary processes of the plain were mainly affected by neotectonic movement, the East Asia monsoon, and human activity. Natural factors have established a vulnerable soil base, and social factors have turned potential vulnerabilities into real hazards (Dianfa et al., 2000). In the late Lower Pleistocene, *Phragmites* accounted for 3.8% of the local vegetation. The landscape consisted of meadow steppe. In the Middle Pleistocene, the natural landscape became forest steppe. Land desertification and a few geochemical periods of salt accumulation occurred (Bian et al., 2008; Zhang & Wang, 2001). In the intermediate stage of the Holocene (7,500–2,500 BP), reeds, marshes, and wetlands became widely distributed. In the late Holocene, the sand dunes were reactivated, and the arid climate led to extensive soda salts and salinization. The hinterland of the plain is a typical alkaline–saline meadow steppe (Bian et al., 2008). A mass of bare alkaline–saline patches has emerged due to regressive succession caused by irrational human exploitation since the 1980s. The effect of revegetation on these bare grounds is not satisfactory (Wu et al., 2005). Since 1990 AD, sandy desertification has been controlled, but land salinization is still expanding (Zhang & Wang, 2001). Most of the grassland area consists of saline meadow soil dominated by *Leymus chinensis* and surrounded by sand dunes (Wu et al., 2005). *P. australis* often grows as a companion species, yet it forms monodominant communities in local low‐lying areas or alkali‐saline patches.

*Phragmites australis* samples were collected from an approximately 10 × 5‐km area within the Pasture Ecology Research Station of Northeast Normal University, Changling, Jilin Province, in Songnen Prairie of China (123°45′E, 44°45′N). The area is influenced by a temperate, semiarid, and semiwet continental monsoon climate. The mean annual precipitation is 313–581 mm, most of which falls between June and August (Li et al., 2014). The four dryland habitats can be described as follows (Figure 1). (a) In the seasonally waterlogged, low‐lying meadow with a pH of 8–8.5 (Habitat 1, designated H1), the monodominant *P. australis* community appears with coverage greater than 85% beginning in July. The companion species are mainly *Echinochloa caudate, Scirpus planiculmis*, and *Polygonum sibiricum*. (b) The alkali‐saline patches lack accumulated rainwater and have a pH greater than 10 (Habitat 2, designated H2). The coverage of the monodominant *P. australis* community is 20%‐40%. The intrinsic soil has been severely destructed. However, after the rainy season, annual halophytes, including *Kochia scoparia*, *Suaeda heteroptera*, *Suaeda corniculata*, *Suaeda glauca*, and *Chloris virgata,* become dominant species at the end of the growing season. (c) The *Leymus chinensis* + Phragmites *australis* community (Habitat 3, designated H3) is the typical community of meadow steppe, with coverage greater than 90%. There is no accumulated rainwater or extremely short‐term accumulation throughout the year. (d) In the sandy soil habitat (Habitat 4, designated H4) with a pH of 8–8.5 and good soil aeration, the community coverage is 75%–80%. In addition to *P. australis, Cleistogenes squarrosa*, *Trigonella ruthenica*, *Lespedeza daurica, Setaria viridis,* and other species also occur in the community (Yang & Lang, 1998; Yang & Li, 2003).

**FIGURE 2:**
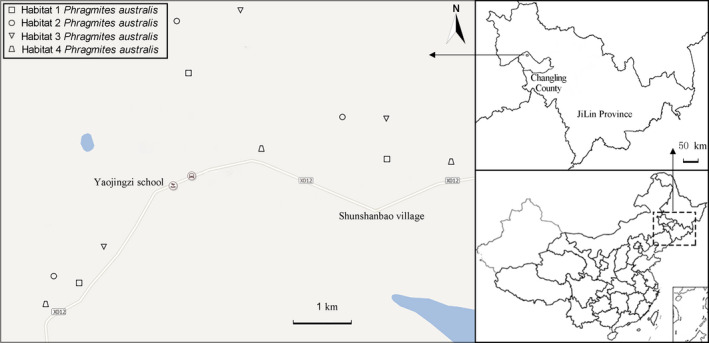
Sampling locations of *Phragmites australis* in 4 habitats. Different habitats are indicated by distinct symbols (square, habitat 1 (H1); circle, habitat 2 (H2); triangle, habitat 3 (H3); trapezoid, habitat 4 (H4))

The samples collected from each habitat were distributed widely because these four habitats lie within a mosaic of diverse patchy habitats. Within each habitat, fully expanded fresh leaves were collected from 20 randomly selected plants, which were distributed in the western, middle, and eastern areas of the research station; thus, three populations were sampled (Figure 2; Table S2). The numbers of individuals at the sites were 6, 7, and 7 in H1; 7, 6, and 7 in H2; 7, 6, and 7 in H3; and 6, 6, and 8 in H4. All the individuals sampled were separated by a minimum of 30 m in case they were members of the same clone. We sampled on a spatial scale that maximized the likelihood of collecting different genotypes in order to represent the full study area. Minimizing the influence of collection on local species conservation and ecological restoration was also considered.

### 2.2 Study material

When the individuals had reached maximum biomass by early September, the aboveground parts were harvested, and the morphological traits of 30 randomly selected individual plants at each site were noted. Leaf length and width refer to the greatest length and width for each plant. Internode length refers to the length between the 5th to 6th nodes from the top. After the plants were divided into stems, leaf blades, and leaf sheaths, all of them were dried at 80°C to a constant weight, and the dry weight was determined. Root tips of all 80 sampled individuals from the four habitats were also taken and subjected to cytological analysis. We found that except for 13 plants whose chromosomes cannot be reliably counted, all the rest 67 plants have a somatic chromosome number of 2*n* = 48.

Five soil samples were collected when individuals were sampled at each site for genetic analysis and were measured for moisture, pH, electrical conductivity (EC), total phosphorus, total nitrogen, organic matter, NO_3_‐nitrogen, NH_4_‐nitrogen, Cl^−^, SO4^2−^, Na^+^, K^+^, Mg^2+^, and Ca^2+^ (Li & Wang, 2012; Su et al., 2005; Walky & Black, 1934; Wu et al., 2009; Zhang & Mu, 2009).

### 2.3 AFLP and MS‐AFLP analyses

Genomic DNA was extracted using a modified cetyltrimethylammonium bromide (CTAB) method (Kidwell & Osborn, 1992). A standard amplified fragment length polymorphism (AFLP) analysis with minor modifications was performed (Vos et al., 1995; Wang et al., 2005). Fluorescently labeled selective amplification primers at the 5’ end (Applied Biosystems Inc., Foster City, CA, USA), a ROX‐500‐labeled internal size standard (Applied Biosystems) and an ABI‐automated 3730XL DNA capillary sequencer were used. The 13 *Eco*RI + 3/*Mse*I + 3 primer pairs (New England Biolabs, Massachusetts, USA) that provided the most reliable, consistently scorable bands were chosen after screening (Table S3). Using the same DNA extracted for the AFLP analysis, we ran two separate protocols for MS‐AFLPs (methylation‐sensitive amplified polymorphisms (MSAPs)) with essentially the same AFLP protocol but replaced the *Mse*I enzyme with *Msp*I or *Hpa*II at the same concentration and chose 10 primers. GeneMapper v.4.1 software (Applied Biosystems) was utilized to analyze the raw data.

We scored all well‐resolved and reliable bands with a binary code: zero for band absent and one for band present. We assessed reliable bands across duplicates for each sample by determining whether banding patterns were consistent between duplicates. For the particular MSAP fragment of every individual, we determined whether the polymorphism was (1) present in both *Eco*RI/*Hpa*II and *Eco*RI/*Msp*I products, (2) absent from both *Eco*RI/*Hpa*II and *Eco*RI/*Msp*I products, or (3) present in only the *Eco*RI/*Hpa*II or the *Eco*RI/*Msp*I product. Condition (1) corresponds to a nonmethylated state, condition (3) indicates a methylated state, and condition (2) was treated as a missing score because it could result from either fragment absence or hypermethylation. Loci were individually classified as either “methylation‐susceptible loci (MSL)” or “nonmethylated loci (NML)” according to whether the observed proportion of discordant *Msp*I/*Hpa*II scores exceeded a given threshold that was set equal to the expected per‐individual probability of obtaining a mismatch of *Msp*I and *Hpa*II scores due to errors (Herrera & Bazaga, 2010). We excluded singleton observations, that is, markers with only one nonconsensus band, from the dataset. All scoring was performed “blindly” by the same person, who lacked any information about the samples. Nonoverlapping peaks in the 150–500 bp range were included, and differences in intensity were taken into account. We did not consider bands that appeared in fewer than four individuals. Genotyping error rates were evaluated for each primer combination by running repeated, independent analyses of six individuals.

### 2.4 Statistical analyses

#### 2.4.1 Soil and plant characteristics

The soil and plant characteristic data were analyzed using SPSS Statistics 17.0 (IBM, New York). When the overall variation in the analysis of variation (ANOVA) was significant, post hoc comparisons among means of soil characteristics were performed (Tukey's honest significant difference (HSD) test, *α* = 0.05). The patterns of variation in plant characteristics among habitats and locations were analyzed with habitat as a fixed factor and location nested within habitat as a random factor. The relationships of different soil and plant characteristics among the 4 habitats were investigated using a principal coordinate analysis (PCA) program (Anderson, 2003, 2003).

#### 2.4.2 Genetic/epigenetic diversity and structure

The levels of genetic diversity were calculated using the POPGENE 1.32 program (Yeh et al., 2000). The parameters included the number of alleles per locus (*N_A_*), the effective allele number per locus (*N_E_*), the percentage of polymorphic loci (*P*), Nei's gene diversity index (*H_E_*), the Shannon information index (*I*), and gene flow (*N_m_*). The percentage of polymorphic bands at the 5% level was calculated by AFLPSURV 1.0 (Vekemans et al., 2002).

We used GENALEX version 6.5 to calculate Nei's genetic distance and Nei's genetic identity for AFLP loci and the MSL of MSAP loci (Peakall & Smouse, 2006). We conducted hierarchical AMOVA to identify variance among habitats, among sites (populations) within habitats, and within sites (populations). Pairwise AMOVA was performed with 9,999 random permutations among all sites to determine which sites were significantly differentiated.

An unweighted pair group method with arithmetic mean (UPGMA) analysis was used to build a dendrogram of the relationships among individuals from the 4 habitats by PAUP4b10 with 1,000 bootstrap replicates (Swofford, 2003).

Principal coordinate analysis (PCoA) based on AFLP and MSAP‐MSL loci was conducted with the package “msap” in R (Pérez‐Figueroa, 2013). Pairwise AMOVA of AFLP and MSAP‐MSL loci was used to calculate Phi_ST between pairs of habitats. Four methylation states were scored: type 1, with the banding pattern of AFLPs through *Hpa*Ⅱ and *Msp*Ⅰ digestion, that is, (1,1), represented “unmethylated”; type 2, with (1,0), represented “hemimethylated”; type 3, with (0,1), represented “internal cytosine methylation”; and type 4, with (0,0), represented “full methylation or absence of target.” AMOVAs in MSL and NML and a comparison of Shannon's diversity index were performed, and the proportions of the four methylation states in the habitats were also calculated.

To gain further perspectives on genetic structure, we implemented the STRUCTURE 2.3 for the AFLPs and MSAPs. *K* varied from 2 to 12 for AFLPs and MSAPs with 10 runs for each *K*, with a burn‐in of 10^5^ followed by 10^5^ Markov chain Monte Carlo (MCMC) iterations (Pritchard et al., 2000). To estimate the real number of clusters (*K*), Δ*K*, an ad hoc quantity related to the second‐order rate of change of the log probability was calculated using the Evanno method (Evanno et al., 2005) in the online version of Structure Harvester v 0.6.9 (Earl & vonHoldt, 2012). We used Arlequin 3.5.1.2 to calculate estimates of genetic and epigenetic differentiation for some individuals between habitats 1 and 3 and habitats 2 and 3 (Excoffier & Lischer, 2010). We then performed locus‐by‐locus AMOVA to characterize significant epigenetic differentiation at some loci.

#### 2.4.3 Identifying outlier loci

To identify candidate loci potentially under selection among habitats, three complementary outlier detection approaches were implemented. A modification of distributed FDIST was performed in Arlequin 3.5.1.2. Outlier loci were identified by comparing empirical *F*_ST_ values for each locus against a null distribution of *F*_ST_ values expected from a neutral drift model based on 20,000 coalescent simulations. Some outliers presented a lower *F*_ST_ value than expected under neutrality, which suggests that they have potentially undergone balancing selection. However, *F*_ST_ values above the upper 99% quantile were considered outlier loci potentially under directional selection (Excoffier & Lischer, 2010).

To cross‐check the reliability of the outlier loci, we also used a Bayesian outlier locus approach by BayeScan 2.1, which implements different algorithms and assumptions compared with those of the former method (Foll & Gaggiotti, 2008). BayeScan uses differences in allele frequencies between populations to identify candidate loci under selection from dominant binary data (as AFLPs) via reversible‐jump Monte Carlo Markov chain (MCMC) algorithm. It is based on the multinomial Dirichlet model. For each locus, the approach involves directly calculating a posterior probability for the model including selection, which is very convenient for making decisions. We used 20 pilot runs, the sample size was set to 5,000, and the thinning interval was set to 10. The loci were ranked according to their estimated posterior probability.

To link outlier loci with specific local environmental conditions, we used another method, that is, the multiple univariate logistic regressions in spatial analysis method (SAM), to identify candidate loci (Joost et al., 2007). Using Samβada v0.5.1, we directly detected associations between the allele and 14 soil parameters. The dataset used for analysis is in the form of a matrix. Each row of the matrix corresponds to an individual, while the columns contain binary information (1 or 0) or values of environmental parameters. The model fit was considered significant when both G and Wald score tests were significant. After Bonferroni's correction of the significance level for multiple comparisons (set to 1.345E−06, corresponding to 95% confidence), the SAM‐detected loci should be associated with soil parameters.

#### 2.4.4 Correlation analyses

Correlations between AFLP pairwise Phi_ST and MSL‐MSAP pairwise Phi_ST, phenotypic variation and genetic/epigenetic differentiation, and genetic/epigenetic differentiation and soil characteristics were tested using SPSS. Mantel test between MSL and NML was tested via the package “msap.”

The allelic frequencies of all the 2,478 loci were used as predictor variables in a distance‐based redundancy analysis (dbRDA; Anderson, 2003) and a distance‐based linear model (DistLM) using the software PRIMER 7 (PRIMER‐E Ltd., New Zealand); both test for a relationship between epigenetic differentiation and adaptive genetic divergence in populations. Marginal permutation test of individual regressors and distlm‐forward method (Anderson, 2003) with 10^5^ permutations and the proportion of the total sum of squares as the criterion for selection was used. A population‐by‐population matrix of epigenetic distances was obtained using the AMOVA‐based population differentiation parameter Φ_ST_. The numbers of loci that had significant or marginally significant effects on epigenetic differences between outliers and nonoutliers were compared to test whether epigenetic loci are more correlated to putatively adapted loci than to loci chosen at random. The biological basis of the statistical relationship in the population‐level dbRDA was verified by an individual‐based dbRDA performed using all the individual plants with binary scores for the genetic and epigenetic data. Storey & Tibshirani's (2003) *q*‐value method for estimation of false discovery rates to the set of *p*‐values for those loci having significant or marginally significant effect was applied. Likelihood ratio χ tests were used to test for significant associations across populations or individuals between outlier locus and epigenetic state through R.

RDA is a form of constrained ordination. The calculation can be simply described as a set of linear regression analyses, where multivariate responses (genotypes) are regressed against multivariate predictors (environmental variables). The RDA was performed to quantify the effects of 14 soil variables on genetic variations of *P. australis* of different habitats using the “vegan” library in R (available at https://www.davidzeleny.net/anadat‐r/doku.php/en:forward_sel) (Oksanen et al., 2013). To avoid inflation of variance components in RDA, we performed forward variable selection in the R package ADESPATIAL.

A genetic/epigenetic distance matrix was used to calculate pairwise population PhiPT values and thus to analyze the correlations between genetic variation, epigenetic variation. and habitat by ZT software (Bonnet & Van de Peer, 2002). The simple Mantel test considers two matrices, while the partial Mantel test involves three matrices. The goal is to test the correlation between two matrices while controlling the effect of a third matrix to remove spurious correlations. Using a simple Mantel test, we tested for a relationship between genetic and epigenetic variations with 10,000 randomizations. A partial Mantel test was used to test for an independent relationship between genetic variation and habitat while controlling for epigenetic variation with 100,000 randomizations, and vice visa. Difference matrices describing the differences of soil parameters among populations were used.

## 3 RESULTS

### 3.1 Variation in morphological traits

Various morphological traits of *P. australis* differed significantly among the 4 habitats (Figure S2). The values of growth traits concerning height, stem diameter, leaf length and width, node number, internode length, and biomass from *P. australis* plant cohorts of H1 and H4 were much greater than those from H3 and H2, with those from H2 usually being the lowest. The stem fractions of *P. australis* from H1 and H3 were higher than those from other habitats. The leaf fraction was highest in plants from H4, followed by H2. The leaf sheath fraction was highest plants of H2.

### 3.2 Genetic and epigenetic diversity and population structure

The 13 primer combinations of AFLP and 10 primer combinations of MSAP profiles produced a total of 2,478 AFLP fragments and 2,884 MSAP fragments with high levels of diversity (Tables S4 and S5). Approximately two‐thirds of the loci were methylation‐sensitive loci (MSL). Overall, all 4 habitats showed high levels of genetic and epigenetic diversity, with H1 showing the highest level of genetic diversity, followed by H3, H2, and then H4 (Tables S6 and S7). Diversity indices indicated the lowest epigenetic diversity in H4, although the differences among H1, H2, and H3 were only moderate. Different DNA methylation levels among habitats were observed. The dendrogram indicated that individuals from the same habitat often grouped together (Figure S4). Individuals from different locations were usually intermingled, suggesting a lack of geographic pattern, which was consistent with the ANOVA test that revealed no significant effect of sampling location on phenotypes (Table S8).

The RDA model considering the effect of soil on genetic structure among habitats was significant (Monte Carlo test with 999 permutations, *F* = 2.0908, *p* = .001) and accounted for the 25.27% variance. The biplot showed the distribution of the 80 samples, demonstrating no major effect of location and separation among habitats (Figure S5). The most important variables were soil Ca^2+^, total nitrogen and phosphorus, organic matter, Mg^2+^, electrical conductivity (EC), NO_3_‐nitrogen, Na^+^, K^+^, and pH (Table S9).

We identified *K* = 3 in STRUCTURE for AFLPs as the most probable number of clusters according to Δ*K*. Location did not play a major role in defining groups of individuals but habitat did. Varying degrees of admixture were found from *K* = 2–4 but it matched general expectations for patterns of relationship across the habitat range (Figure S6). It is suggested that when the water level of swamp area drops, meadow reeds were formed. When the meadow completely dried up, alkali‐saline patches appeared. The salinized soil is further covered by sand layer and sand reed grows. *K* = 4 was identified as the most probable cluster number for MSAPs. The figure showed more remarkable differentiation among habitats than AFLPs with the pattern a little different.

**FIGURE 3:**
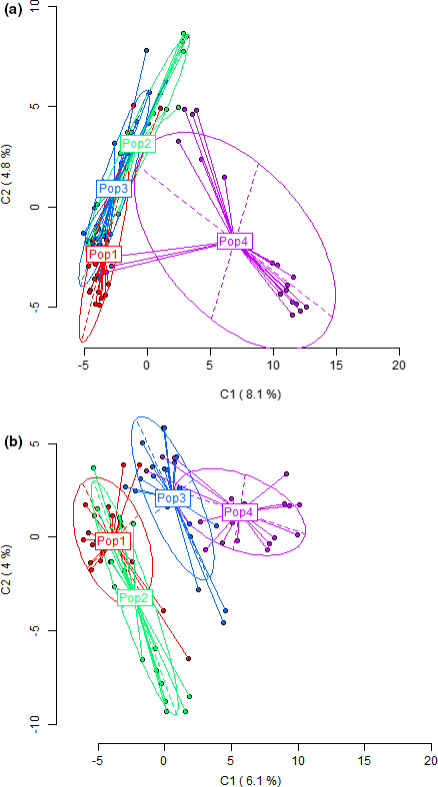
Principal coordinate analysis (PCoA) based on AFLP (a) and MSAP (b) banding patterns, showing the relationships among individuals from the four habitats

Hierarchical AMOVA revealed both significant genetic and epigenetic differentiation between habitats, among populations (sampling sites), and within populations (in all cases *p* < .001) (Table 1). Most variation existed within rather than among habitats. All pairwise comparisons between the 4 habitats were significant (Table 2). The pairs between H4 and H1, H4 and H2, and H4 and H3 showed more genetic and epigenetic differentiation than other pairs. The PCoA plot showed consistent results (Figure 3).

**TABLE 1:** Results of AMOVA with respect to *Phragmites australis* within and among habitats based on AFLP (a) and MSAP (b) banding patterns (with or without populations within habitats according to sites)

Source	*df*	SS	Variance components	% variation	*p*‐Value
(a)					
**Without populations**					
Among habitats	3	2,857.963	31.79406	9.12	
Within habitats	76	24,074.75	316.773	90.88	
Total	79	26,932.713	348.5671	100	
PhiPT = 0.09121					*p* < .001
**With populations**					
Among habitats	3	2,857.963	22.82091	6.55	*p* < .001
Among populations within habitats	8	3,949.042	29.77987	8.54	*p* < .001
Within populations	68	20,125.708	295.9663	84.91	*p* < .001
Total	79	26,932.713	348.5671	100	
PhiPT = 0.15091					
(b)					
**Without populations**					
Among habitats	3	3,730.200	38.686	7.61	
Within habitats	76	35,694.950	469.670	92.39	
Total	79	39,425.150	508.357	100	
PhiPT = 0.076					*p* < .001
**With populations**					
Among habitats	3	3,730.200	31.703	6.24	*p* < .001
Among populations within habitats	8	4,858.492	23.177	4.56	*p* < .001
Within populations	68	30,836.458	453.477	89.20	*p* < .001
Total	79	39,425.150	508.357	100	
PhiPT = 0.108					

**TABLE 2:** Pairwise AMOVA among *Phragmites australis* in 4 different habitats based on AFLP markers (below the diagonal) and methylation‐susceptible loci (MSL) of MSAP markers (above the diagonal)

Habitat	H1	H2	H3	H4
H1		0.02318**	0.05297***	0.1193***
H2	0.06548***		0.05827***	0.1026***
H3	0.0394***	0.02731*		0.05864***
H4	0.142***	0.1267***	0.1421***	

Note

*p < .01, **p < .001, ***p < .0001.

### 3.3 Epigenetic differentiation in relation to adaptive genetic divergence

Three complementary approaches were used to identify candidate genomic regions (outliers) corresponding to loci potentially influenced by directional selection or linked to a locus under selection. According to modified FDIST, 24 outliers (0.97%) showed higher *F*_ST_ values than expected under neutrality at the 99.5% probability level (Figure S7; Table S10). BayeScan analyses identified 18 candidate loci (0.73%) under selection on Jeffrey's scale (Foll & Gaggiotti, 2008). Three loci were “substantial” (0.91 > posterior probability (PP) > 0.76, 1 > log10(PO) > 0.5); 4 loci were “strong” (0.97 > PP > 0.91, 1.5 > log10(PO) > 1); 2 loci were “very strong” (0.99 > PP > 0.97, 2 > log10(PO) > 1.5); and 9 loci were “decisive” (1 > PP > 0.99, log10(PO) > 2) (Figure S8, Table S11). According to Samβada (95% confidence), univariate logistic regression models that tested AFLP marker frequency variation for the environmental variables indicated 13 loci (0.52%) for both the G test and the more stringent Wald test (Table 3). The most associated environmental variable was soil Ca^2+^ content, followed by total P content, Mg^2+^ content, pH, soil moisture, organic matter, and total nitrogen.

**TABLE 3:** Significant associations between AFLP loci and environmental parameters, as indicated by Samβada analysis with a significance threshold (ST) set to 95% (corresponding to *p* < 1.345E−06)

	Environmental parameter	*p*	Environmental parameter	*p*
M1899	Ca^2+^(mg/L)	1.61421E−08		
M1834	Ca^2+^(mg/L)	2.15227E−08		
M1962	Ca^2+^(mg/L)	8.07103E−08		
M1864	Ca^2+^(mg/L)	1.34517E−07		
M862	Ca^2+^(mg/L)	1.61421E−08	pH	1.07614E−06
M1957	Total P(g/kg)	2.15227E−07	Moisture(%)	2.15227E−07
	Organic matter(g/kg)	8.1E−07	Total *N*(g/kg)	1.21065E−06
M1940	Ca^2+^(mg/L)	5.38068E−07		
M1917	Total P((g/kg))	8.07103E−07	Mg^2+^(mg/L)	1.07614E−06
M1900	Total P(g/kg)	1.07614E−06	Mg^2+^(mg/L)	1.34517E−06
M1961	Mg^2+^(mg/L)	1.21065E−06		
M1918	Ca^2+^(mg/L)	1.21065E−06		
M2139	Ca^2+^(mg/L)	1.21065E−06		
M945	pH	1.34517E−06		

A total of 33 loci (1.33%) were identified by these 3 approaches. The loci identified with Samβada were the same as those identified with BayeScan, with the exception of 1 locus. Most of the outliers identified by BayeScan were consistent with those identified using these two methods (Figure S9). One locus, 1,248, of the 3 loci (0.91 > PP) was not detected by modified FDIST and Samβada and was therefore not included. A dbRDA, in which the 12 × 12 matrix of AMOVA‐based epigenetic distances between populations was regressed on the population allelic frequencies for all the AFLP loci (Table 4). The first two axes of the RDA projection represent 78.65% (RDA1) and 12.84% (RDA2) of the explained variance, respectively. Twenty of the total 32 outlier loci and 675 of 2,446 nonoutliers had significant or marginally significant effects on epigenetic differences, indicating that outliers (62.5% of total) are more significantly correlated with epigenetic loci than nonoutliers (27.60% of total) (χ = 19.069, *df* = 1, *p* = 1.261e−05) (Table 4, Table S12). Individual‐based DistLM also revealed that outliers (31 of 32 loci, 96.875%) are significantly higher than nonoutliers (1,208 of 2,446 loci, 49.387%) to be correlated with epigenetic loci (χ = 28.493, *df* = 1, *p* = 9.404e−08) (Table 4, Table S12). All the q‐values were consistent with *p*‐values.

**TABLE 4:** DistLM analysis testing for, first, a relationship between epigenetic differences among *Phragmites australis* populations in the form of a resemblance matrix and the allelic frequencies in each population for all the AFLP loci (“Population‐level analysis”) and, second, a relationship between individual epigenotypes and genotypes across 80 individual plants (“Individual‐level analysis”)

AFLP outlier locus	Population‐level analysis (*n* = 12)			Individual‐level analysis (*n* = 80)		
	Pseudo‐*F*	*p*‐value	*q*‐value	Pseudo‐*F*	*p*‐value	*q*‐value
M116	24.466	.0008	0.003	2.536	.00001	0.00010
M362	25.253	.0016	0.004	2.6699	.00001	0.00010
M372	10.245	.0002	0.002	1.7743	.00001	0.00010
M500	23.478	.0078	0.0104	2.7358	.00001	0.00010
M501	23.055	.0023	0.0046	2.4952	.00001	0.00010
M502	12.503	.0027	0.004909	1.755	.00001	0.00010
M645	25.537	.0009	0.003	2.6262	.00001	0.00010
M1246	11.628	.0046	0.007077	2.1438	.00001	0.00010
M1263	3.5858	.0524	0.055158	1.4384	.00005	0.00034
M1482	11.371	.0004	0.002	1.9028	.00001	0.00010
M1620	13.901	.0014	0.004	2.2765	.00001	0.00010
M1644	14.778	.0004	0.002	2.1264	.00001	0.00010
M1695	8.5273	.0038	0.006333	2.01	.00001	0.00010
M1864	3.5575	.051	0.055158	1.4673	.00004	0.00030
M1881	16.612	.0004	0.002	2.0521	.00001	0.00010
M1940	3.0536	.0791	0.0791	1.3594	.00030	0.00139
M1954	6.8647	.0162	0.019059	1.5324	.00002	0.00017
M2139	7.3061	.0136	0.017	1.5731	.00001	0.00010
M2163	19.669	.0018	0.004	2.4024	.00001	0.00010
M2164	7.8908	.0065	0.009286	1.814	.00001	0.00010
M862				1.2608	.00210	0.00638
M945				1.2311	.00360	0.00963
M1819				1.0983	.06650	0.07643
M1834				1.18	.01250	0.02424
M1899				1.2909	.00120	0.00414
M1917				1.0972	.06850	0.07815
M1918				1.2688	.00190	0.00590
M1957				1.1642	.01640	0.02862
M1961				1.4349	.00004	0.00030
M1962				1.1503	.02240	0.03600
M1973				1.2723	.00160	0.00520

Note

Twenty loci and 31 loci of the total 32 outliers which revealed significant or marginally significant *p*‐values and *q*‐values at the population and individual level are shown, respectively. Results correspond to Marginal tests using every AFLP locus as single variable with a forward selection procedure and proportion of the total sum of squares as selection criterion.

### 3.4 Comparison between genetic and epigenetic variations

AFLP pairwise Phi_ST had a positive correlation with MSAP‐MSL pairwise Phi_ST (Figure S10; *R* ^2^ = 0.716, *p* < .001). We also detected a significant correlation between the matrices of MSL‐based distances and NML‐based distances (*r* = 0.4751653, *p* = .000999). A significant correlation between genetic variation and epigenetic variation was revealed by the Mantel test (*r* = 0.926559, *p* < .001).

The mean Shannon's diversity index (*I*) for the polymorphic MSL (*M* ± *SD* = 0.514 ± 0.159) was significantly higher than the corresponding value for the NML (*M* ± *SD* = 0.244 ± 0.139) (*p* < .0001, Wilcoxon rank sum test). Therefore, it was apparent that methylation‐based epigenetic diversity exceeded genetic diversity when the two magnitudes were compared using the same index. H1, H2, and H3 displayed a higher value of all diversity indices for genetic compared with epigenetic variation, in contrast to H4, which showed a higher level of epigenetic diversity than genetic diversity. There was more epigenetic variance than genetic variance within habitats (Table 1). The genetic variance among habitats (PhiPT = 0.09121) was greater than the epigenetic variance among habitats (PhiPT = 0.076). Consistently, the MSAP data revealed greater differences for NML (Phi_ST = 0.09027) than for MSL (Phi_ST = 0.06992) (Table S13), suggesting that genetic variation may be more strongly structured than epigenetic variation by the environment, making independent epigenetic variation inconspicuous. However, the percentage of variance explained by habitat (6.55%) in the hierarchical AMOVA of AFLPs was smaller than that explained by “among populations” (8.54%), while the percentage of variance explained by habitat (6.24%) for MSAPs was greater than that explained by “among populations” (4.56%), indicating that some epigenetic loci may respond to different local habitats (Table 1).

Pairwise AMOVA of habitats revealed two pairs, that is, H1 and H3, and H2 and H3, whose genetic differentiation was smaller than their epigenetic differentiation (Table 2). Pairwise genetic and epigenetic distances and the PCoA plot showed consistent results (Table S14; Figure 3). Thirteen of 66 pairwise comparisons between sites showed incongruence between epigenetic and genetic markers (Table 5). The 13 pairs included 8 pairs that were significant at genetic but not epigenetic markers and 5 pairs (2 between H1 and H3; 3 between H2 and H3) that were significant for epigenetic but not genetic markers, suggesting that at least some portion of the epigenetic differentiation was not dependent on genetic differentiation.

**TABLE 5:** Pairwise AMOVA among *Phragmites australis* from 12 different sites based on AFLP and MSAP markers. The results from AFLP markers are presented below the diagonal, and those from MSAP markers are presented above the diagonal. An asterisk denotes statistical significance (α = 0.01)

	Site 1	Site 2	Site 3	Site 4	Site 5	Site 6	Site 7	Site 8	Site 9	Site 10	Site 11	Site 12
Site 1		0.013	0.020	0.024	0.092*	0.025	0.067*	0.067*	0.086*	0.220*	0.188*	0.094*
Site 2	0.023		0.006	0.012	0.098*	0.021*	0.077*	0.063*	0.071*	0.221*	0.183*	0.088*
Ste 3	0.048*	0.008		0.028	0.118*	0.020*	0.087*	0.066*	0.075*	0.222*	0.193*	0.089*
Site 4	0.098*	0.085*	0.101*		0.026	0.028	0.055*	0.069*	0.086*	0.179*	0.143*	0.068*
Site 5	0.145*	0.152*	0.166*	0.064		0.107*	0.108*	0.134*	0.154*	0.230*	0.199*	0.119*
Site 6	0.076*	0.052*	0.066*	0.052*	0.154*		0.100*	0.081*	0.083*	0.232*	0.204*	0.099*
Site 7	0.053	0.080*	0.110*	0.052	0.101*	0.060		0.032	0.055*	0.142*	0.109*	0.038*
Site 8	0.041	0.043*	0.060*	0.074*	0.128*	0.030	0.051		0.038*	0.176*	0.146*	0.037
Site 9	0.074*	0.056*	0.073*	0.085*	0.146*	0.040*	0.073*	0.046*		0.186*	0.156*	0.049*
Site10	0.309*	0.305*	0.319*	0.273*	0.324*	0.330*	0.299*	0.336*	0.328*		0.000	0.136*
Site11	0.231*	0.239*	0.250*	0.214*	0.249*	0.271*	0.230*	0.262*	0.255*	0.078*		0.093*
Site12	0.119*	0.098*	0.102*	0.111*	0.153*	0.139*	0.123*	0.112*	0.140*	0.285*	0.189*	

Note

Sites 1–3 correspond to H1, sites 4–6 correspond to H2, sites 7–9 correspond to H3, and sites 10–12 correspond to H4.

### 3.5 Studies specifically among the three habitats (H1, H2, and H3)

The analysis of individuals with nonsignificant genetic differentiation more clearly suggested the potential contribution of epigenetic variation to plant ecological differentiation in response to different habitats. AMOVA revealed significant epigenetic differentiation (*F*_ST_ = 0.01836, *p* = .03715) but nonsignificant genetic differentiation (*F*_ST_ = 0.03042, *p* = .07625) between 9 individuals from H1 and 9 individuals from H3 (Figure S11). Locus‐by‐locus AMOVA showed that 6 loci were significantly differentiated between habitats (alpha = 0.01) (Table S15, Figure S11). Likewise, significant epigenetic differentiation (*F*_ST_ = 0.04012, *p* < .0001) but nonsignificant genetic differentiation (*F*_ST_ = 0.02102, *p* = .05572) was found between 7 individuals from H2 and 15 individuals from H3, and 23 loci were significantly differentiated, indicating that the methylation patterns at these loci were different between habitats.

To test for independent epigenetic variation, the correlations between genetic variation, epigenetic variation, and habitat for all individuals across all sites in H1, H2, and H3 were analyzed (Table 6). A significant correlation between genetic and epigenetic variations (*r* = 0.6596, *p* = .0001) was found. Both epigenetic variation and genetic variation were significantly correlated with most soil variables. The values of correlation coefficients between epigenetic and environmental distances, with variables including soil moisture, total phosphorus (P), total nitrogen (N), organic matter, Ca^2+^, and Mg^2+^, were greater than the values of those between genetic and those environmental distances when we controlled for the correlation between genetic and epigenetic variations. However, the correlation values between genetic and environmental distances such as soil pH, conductivity, NO^3^‐N, NH_4_‐N, Cl^−^, SO_4_^2−^, K^+^, and Na^+^ were greater than those between epigenetic and environmental distances. Thus, our data suggest epigenetic variation was more affected by soil nutrient availability than was genetic variation.

**TABLE 6:** The independent relationships between genetic variation or epigenetic variation and habitat for all individuals across all sites in Habitats 1, 2, and 3 were calculated via a partial Mantel test. The *p*‐value and *r* (correlation coefficient) are shown

Variable	Genetic variation		Epigenetic variation	
	*r*	*p*	*r*	*p*
Soil moisture	0.241	.084.	−0.292	.046*
pH	0.429	.002**	−0.406	.001**
Soil conductivity	0.430	.001**	−0.270	.024*
Total phosphorus	0.217	.111	−0.263	.075.
Total nitrogen	0.407	.004**	−0.430	.000***
NO3‐nitrogen	0.397	.012*	−0.218	.079.
NH_4_‐nitrogen	0.334	.026*	−0.033	.440
organic matter	0.340	.014*	−0.352	.002**
Cl^−^	0.369	.013*	−0.169	.137
SO_4_^2−^	0.419	.007**	−0.259	.042*
Na^+^	0.432	.001**	−0.278	.020*
K^+^	0.436	.0009***	−0.288	.017*
Mg^2+^	0.233	.092.	−0.309	.025*
Ca^2+^	0.303	.027*	−0.333	.006**

Note

***p < .001; ** .01; * .05; .1. Euclidean genetic and epigenetic distance matrices and difference matrices describing the differences of soil parameters among populations were used.

Trait variation was significantly correlated with almost all the soil parameters (Table S16). Among all traits measured, only node number increased significantly with increasing genetic diversity, that is, *H_e_* and *I* (Table S17). The height, leaf length, internode length, node number, stem biomass, leaf biomass, leaf sheath biomass, and total biomass, all decreased (except for the leaf sheath fraction, which increased) significantly with an increase in the mean epiallelic frequency of (0,1) detected by *Hpa*Ⅱ and *Msp*Ⅰ among the MSL.

## 4 DISCUSSION

### 4.1 Morphological traits among habitats

In 3 of the 4 habitats, traits were significantly correlated with almost all the soil environmental factors (Table S16). Biomass allocation among habitats provided evidence of trade‐offs between the leaf fraction and stem fraction (significantly negatively correlated). The growth parameters in H2 were usually the lowest, but the leaf fraction and leaf width were not (Figure S2). Node number, which was equivalent to leaf number, was similar in H2 to that in H3. Thus, *P. australis* from H2 was likely to have maximized photosynthetic efficacy. In addition, it has been suggested that plants under saline or sand dune conditions store more water in leaves to enable adaptive responses, which might explain the higher leaf fractions in H2 and H4 (Yang et al., 2014). In H1 and H3, with higher community coverage, plants may maximize light interception by elongating seedling stems and producing more leaves, which may explain the higher stem fraction. The leaf sheath of Poaceae plants provides physical support and protection and enhances the transport of nutrients and photosynthetic products (Guo et al., 2014). For example, the leaf sheath fraction was higher in an invasive common reed population in North America than in native European genotypes. The highest leaf sheath fraction detected in H2 could therefore confer a competitive advantage. The soil moisture and nutrients, including phosphorus, nitrogen, and organic matter, in H4 were similar to those in H3, whereas various ions, except for Ca^2+^ and Mg^2+^, were the lowest, with the soil being alkaline. We also found that the growth parameters were significantly positively correlated with Mg^2+^ and Ca^2+^ (data not shown). These results may explain why the growth parameters in H4 were always higher than those in H2 and H3, thereby indicating that the soil desertification in H4 was not very serious. Therefore, the variation in many traits in this set of *P. australis* variants from 4 habitats reflects the ability to acquire light resources and functionally accommodate alkaline–saline conditions; thus, they are putatively adaptive traits.

### 4.2 Diversity and structure of habitat genetic variation

The levels of genetic diversity were generally high, based on a comparison of the mean population‐level measures (na = 1.556, *p* = 55.615, *H_E_* =0.202) with those of *P. australis* in the eastern half of the United States (Pellegrin & Hauber, 1999), northeastern North America (Kirk et al., 2011), the Po Plain and Razim in Europe (Lambertini et al., 2008), and the Yellow River Delta and the Songnen Steppe of China (Guo et al., 2003; Li et al., 2009). This is partly because sexual reproduction may occur more frequently in the study area than in other areas, despite clonal reproduction being the most typical form of reproduction. A population history including multiple founder events or reproduction with new colonizers might also explain this result (Zhang & Wang, 2001). The autumn monsoon promoting dispersal and selection of beneficial somatic mutations are also plausible explanations. The lowest genetic diversity index being detected in H4 is consistent with the niche‐width variation hypothesis (Valen, 1965). Populations growing in a broader niche (e.g., alkaline–saline meadow soil of H1, H2, and H3) are hypothesized to exhibit more variability than populations in a narrower niche (e.g., sandy soil of H4). Among factors contributing to spatial genetic structure involving habitat induction, phylogeographic history, gene flow, genetic drift, population demographic processes, and selection pressure should be major ones. Environmental heterogeneity generates different selection pressures and divergent selection, which in turn may lead to genetic heterogeneity, local adaptation, ecological speciation, and even reproductive isolation (Mark et al., 2015). Meanwhile, it may constrain gene flow via variation in phenology. Genetic differentiation is observed at small scales whenever localized selection is sufficiently intense at a lower or higher level or in the absence of gene flow (Defaveri & Merilä, 2014; He et al., 2019). In particular, we have detected outlier loci. Although loci across the genome are influenced by genome‐wide evolutionary forces, selection is locus‐specific and imprints a particular pattern of variability.

### 4.3 Diversity and structure of habitat epigenetic variation

MS‐AFLP analysis offers a genome‐wide snapshot for nonmodel organisms that lack sequenced genomes. It accommodates the large sample sizes necessary for population‐level studies and allows direct comparisons of genetic and epigenetic variation. Evolution relies on heritable phenotypic diversity. In addition to genetic variation, epigenetic variation can also generate different phenotypes by conferring different levels of space‐temporal gene expression and even result in adaptive phenotypic divergence and fitness differences. Before this role can be validated, fundamental questions including the magnitude, structure, and degree of autonomy of epigenetic variation in relation to genetic context need to be addressed. The high levels of epigenetic diversity that we found can serve as a critical prerequisite for microevolution, consistent with results for the violet *Viola cazorlensis* (Herrera & Bazaga, 2010). Similar to invasive *P. australis* being found to harbor more epigenetic variation than its native counterparts in midcoastal Maine (Spens & Douhovnikoff, 2016), the higher epigenetic diversity in H1, H2, and H3 than in H4 may suggest that increased epigenetic diversity facilitates a wider distribution. The significant epigenetic differentiation among habitats suggested that epigenetic diversity might have been structured by environmental gradients. STRUCTURE identified groups of individuals via ∆*K* which is a good predictor of the real number of clusters. We found habitat other than location is the major factor in shaping genetic and epigenetic structure. As such, we addressed whether such structuring can be interpreted as adaptive.

### 4.4 The relationship between genetic and epigenetic variation

In theory, in a genetically coherent panmictic unit with extensive gene flow among populations, a significant correlation between epigenetic differentiation and adaptive genetic divergence would provide a possibility for functionally linked epigenetic and genetic variation as an evolutionary unit (Herrera & Bazaga, 2010). We indeed found such a correlation.

Since functional DNA methylation is largely under genetic control, we have very limited data on epigenetic differentiation in natural populations that is not explained by genetic variation. Although clonal organisms, organisms with low genetic diversity, or approaches for artificially regulating methylation have been used to explore relationships between epigenetic variation and the environment (Thieme et al., 2017), as studies expand into more nonmodel organisms, researchers must consider the relationship between genetic and epigenetic variations in the context of their coexistence. More traits were correlated significantly with epigenetic variation with a mean epiallelic frequency of (0,1) than with genetic variation. Given that a recent study revealed that the (0,1) epiloci are much more stable than the (1,0) epiloci across asexual generations (Shi et al., 2019), our results clearly point to the effect of epigenetic variation on adaptation. Specifically, the H4 group possessed the lowest genetic diversity, but it increased in its epigenetic diversity. However, the epigenetic diversity of H1, H2, and H3 groups decreased compared with its genetic diversity. The two pairs, that is, H1 and H3 and H2 and H3, whose genetic differentiation was the lowest showed higher epigenetic differentiation. Since epigenetic diversity could partially explain the incongruence between lower genetic diversity and a broader distribution in *P. australis* of the DELTA and the initialized invasion of Kenyan house sparrows, our findings revealed a trend suggesting that epigenetic variation functions in compensating for decreased genetic variation to contribute to broad ecological adaptation (Foust et al., 2015; Liu et al., 2018).

### 4.5 Implications for global change responses

In addition, when assessing global change responses of plant species, intraspecific variation is suggested to be considered. According to CRC (cause–response–consequence) model, global change driver affects lineages composed of different genotypes or ecotypes (Eller et al., 2017). The likely responses of the genotypes with highly phenotypic plasticity which may be associated with methylation variation are acclimation, increased fitness, and range expansion. Thanks to the compensation effect, the epigenetic diversity of H4 was not too much low. While the genotypes with low plasticity undergo range shift or local extinction. Likewise, the lineage with high phenotypic diversity would be more likely to survive just like the *P. australis* in the Songnen Prairie of China. Considering the reports that the salt tolerance of *P. australis* is genotype‐specific rather than lineage‐specific, we should pay attention to such genotype tolerant to specific global change driver for other plants for restoration or management. Response of *P. australis* and other plant types mediated by interactions with other drivers including temperature and atmospheric CO_2_ on a global scale need to be further investigated due to patchy changes such as soil salinity.

## 5 CONCLUSIONS

Together, our results suggest that epigenetic variation is correlated with genetic variation, and both may contribute to adaptation to stressful environments in the cosmopolitan grass, *P. australis*. Before a population acquires adequate genetic diversity through propagation at the intra‐ or interhabitat level, epigenetic variation may rapidly emerge. Thus, our study adds to the growing ecological and evolutionary genomics studies reporting on population‐level responses to heterogeneous environments and “genetic‐independent” epigenetic variation on a nonmodel plant species, which can offer valuable insights into global change responses of plant species. Determining if and to which extent these environment‐induced epigenetic variations are heritable when decoupled from the induction condition would entail common garden studies for multiple generations. In theory, even if the epigenetic state is stable for only a few generations, it may exert an effect on evolutionary trajectories. Reduced representation bisulfite sequencing approaches and detection of differentially methylated regions (DMRs) will be important next steps in exploring the functional significance of epigenetic variation, allowing the direct comparison of genetic and epigenetic variations, and in turn, allowing the differentiation of “facilitated” and “pure” epigenetic variation. Then, carefully designed experiments, such as targeted bisulfite sequencing or expression of candidate loci in knockouts or transgenic manipulations, can be used to investigate the autonomy and importance of epigenetic effects.

## CONFLICT OF INTEREST

The authors have no conflict of interest to declare.

## AUTHOR CONTRIBUTION

**Tian Qiu:** Investigation (equal); Writing‐original draft (equal). **Zhiyuan Liu:** Investigation (equal); Software (equal). **Yunfei Yang:** Conceptualization (equal); Supervision (equal). **Bao Liu:** Supervision (equal); Writing‐review & editing (equal).

## Supporting information

Appendix S1

## Data Availability

We would like to archive our data in a publicly accessible repository, that is, Dryad. The dataset (“TianQiu data”) has been assigned a unique identifier, called a DOI (https://doi.org/10.5061/dryad.gtht76hmt), and it can be found on https://doi.org/10.5061/dryad.gtht76hmt.
