# Supplementary material for: Epigenetic variation associated with responses to different habitats in the context of genetic divergence in Phragmites australis
Source: Ecol Evol. 2021 Jul 30;11(17):11874–89. doi: 10.1002/ece3.7954 (PMC8427615; doi:10.1002/ece3.7954)
Supplement: Supplementary file 1 — Appendix S1 [file ECE3-11-11874-s001.docx]

**Appendix**

**TABLE S1-S17，FIGURE S1-S11：**

**TABLE S1** Soil characteristics in 4 different habitats from the field survey and their comparison (M±SE, different letters indicate significant differences between habitats at the 5% level.)

| Habitat | H1 | H2 | H3 | H4 |
| --- | --- | --- | --- | --- |
| Soil moisture (%) | 16.87±1.87a | 6.02±0.5b | 6.34±0.76b | 6.53±0.27b |
| pH | 8.16±0.12c | 10.83±0.07a | 9.54±0.16b | 8.47±0.06c |
| Conductivity (us cm^-1^) | 142.94±11.29b | 628.92±77.92a | 189.38±34.69b | 75.94±2.52c |
| Content of total P (g kg^-1^) | 0.27±0.03a | 0.13±0.01b | 0.13±0.01b | 0.17±0.01b |
| Content of total N (g kg^-1^) | 1.18±0.15a | 0.4±0.03c | 0.7±0.08ab | 0.6±0.04b |
| Organic matter (g kg^-1^) | 11.5±1.27a | 5.37±0.89b | 6.68±1.11b | 5.29±0.47b |
| NO_3_-N (mg kg^-1^) | 5.25±0.18a | 3.41±0.18b | 5.78±0.49a | 4.62±0.4ab |
| NH_4_-N (mg kg^-1^) | 10.33±1.08b | 99.67±13.55a | 7.21±0.83b | 9.13±0.92b |
| Cl^-^ (mg L^-1^) | 6.6±0.8b | 15.93±0.43a | 5.6±0.27b | 3.09±0.23c |
| SO4^2-^ (mg L^-1^) | 3.47±0.32b | 33.7±6.21a | 3.73±0.62b | 1.74±0.14c |
| Na^+^ (mg L^-1^) | 18.27±1.62b | 103.63±12.59a | 25.96±4.15b | 2.4±0.41c |
| K^+^ (mg L^-1^) | 6.99±0.2bc | 29.31±0.47a | 10±1.86b | 4.36±0.28c |
| Mg^2+^ (mg L^-1^) | 3.76±0.65a | 1.24±0.02b | 1.31±0.41b | 2.44±0.27ab |
| Ca^2+^ (mg L^-1^) | 2.17±0.33a | 0.39±0.01c | 0.7±0.08b | 2.73±0.21a |

**TABLE S2** Geographical information of site locations used for sampling.

| Site | Location information | Longitude (E) | Latitude (N) |
| --- | --- | --- | --- |
| 1 | West H1 | 123.496 | 44.553 |
| 2 | West H2 | 123.49 | 44.555 |
| 3 | West H3 | 123.501 | 44.56 |
| 4 | West H4 | 123.488 | 44.55 |
| 5 | Middle H1 | 123.521 | 44.589 |
| 6 | Middle H2 | 123.519 | 44.599 |
| 7 | Middle H3 | 123.533 | 44.599 |
| 8 | Middle H4 | 123.538 | 44.576 |
| 9 | East H1 | 123.568 | 44.574 |
| 10 | East H2 | 123.557 | 44.582 |
| 11 | East H3 | 123.567 | 44.581 |
| 12 | East H4 | 123.583 | 44.574 |

**TABLE S3** The adaptors, preselective primers, and primer pairs for selective amplification used for the AFLP and MSAP analyses.

|  | | | | Sequence | | | | | | | | | |  |  |
| --- | --- | --- | --- | --- | --- | --- | --- | --- | --- | --- | --- | --- | --- | --- | --- |
| Adaptor | | | |  | | | | | | | | | |  |  |
| *EcoR*Ⅰ-adaptor 1 | | | | 5'-CTCGTAGACTGCGTACC-3' | | | | | | | | | |  |  |
| *EcoR*Ⅰ-adaptor 2 | | | | 5'-AATTGGTACGCAGTCTAC-3' | | | | | | | | | |  |  |
| *Mse*Ⅰ-adaptor 1 | | | | 5'-GACGATGAGTCCTGAG-3' | | | | | | | | | |  |  |
| *Mse*Ⅰ-adaptor 2 | | | | 5'-TACTCAGGACTCAT-3' | | | | | | | | | |  |  |
| *Hpa*Ⅱ/*Msp*Ⅰ-adaptor 1 | | | | 5'-GACGATGAGTCTAGAA-3' | | | | | | | | | |  |  |
| *Hpa*Ⅱ/*Msp*Ⅰ-adaptor 2 | | | | 5'-CGTTCTAGACTCATC-3' | | | | | | | | | |  |  |
| Pre-selective primer | | | | | | | | | | | | | |  |  |
| *EcoR*Ⅰ+A | | | | 5'-GACTGCGTACCAATTCA-3' | | | | | | | | | |  |  |
| *Mse*Ⅰ+C | | | | 5'-GATGAGTCCTGAGTAAC-3' | | | | | | | | | |  |  |
| *Hpa*Ⅱ/*Msp*Ⅰ+T | | | | 5'-GATGAGTCTAGAACGGT-3' | | | | | | | | | |  |  |
| *EcoR*Ⅰ+3 primer (without or with 5'-FAM) | | | | | | | | | | | | | |  |  |
| a. *E*-AAC | | | | 5'-GACTGCGTACCAATTCAAC-3' | | | | | | | | | |  |  |
| b*. E-*AAG | | | | 5'-GACTGCGTACCAATTCAAG-3' | | | | | | | | | |  |  |
| c. *E*-ACA | | | | 5'-GACTGCGTACCAATTCACA-3' | | | | | | | | | |  |  |
| d. *E*-ACT | | | | 5'-GACTGCGTACCAATTCACT-3' | | | | | | | | | |  |  |
| g. *E*-AGC | | | | 5'-GACTGCGTACCAATTCAGC-3' | | | | | | | | | |  |  |
| h. *E*-AGG | | | | 5'-GACTGCGTACCAATTCAGG-3' | | | | | | | | | |  |  |
| i. *E*-AGA | | | | 5'-GACTGCGTACCAATTCAGA-3' | | | | | | | | | |  |  |
| j. *E*-ATC | | | | 5'-GACTGCGTACCAATTCATC-3' | | | | | | | | | |  |  |
| *Mse*Ⅰ+3 primer | | | | | | | | | | | | | |  |  |
| 2. *M*-CAC | | | | 5'-GATGAGTCCTGAGTAACAC-3' | | | | | | | | | |  |  |
| 3. *M*-CAG | | | | 5'-GATGAGTCCTGAGTAACAG-3' | | | | | | | | | |  |  |
| 4. *M*-CAT | | | | 5'-GATGAGTCCTGAGTAACAT-3' | | | | | | | | | |  |  |
| 5. *M*-CTA | | | | 5'-GATGAGTCCTGAGTAACTA-3' | | | | | | | | | |  |  |
| 6. *M*-CTC | | | | 5'-GATGAGTCCTGAGTAACTC-3' | | | | | | | | | |  |  |
| 7. *M*-CTG | | | | 5'-GATGAGTCCTGAGTAACTG-3' | | | | | | | | | |  |  |
| 8. *M*-CTT | | | | 5'-GATGAGTCCTGAGTAACTT-3' | | | | | | | | | |  |  |
| *Hpa*Ⅱ/*Msp*Ⅰ+3 primer | | | | | | | | | | | | | |  |  |
| 3. *H/M*-TCT | | | | 5'-GATGAGTCTAGAACGGTCT-3' | | | | | | | | | |  |  |
| 5. *H/M*-TTC | | | | 5'-GATGAGTCTAGAACGGTTC-3' | | | | | | | | | |  |  |
| 6. *H/M*-TTG | | | | 5'-GATGAGTCTAGAACGGTTG-3' | | | | | | | | | |  |  |
| 7. *H/M*-TTA | | | | 5'-GATGAGTCTAGAACGGTTA-3' | | | | | | | | | |  |  |
| primer combinations | | | | | | | | | | | | | |  |  |
| *EcoR*Ⅰ+3 | a | b | c | | i | c | i | i | a | j | j | j | j | | i |
| *Mse*Ⅰ+3 | 7 | 4 | 3 | | 4 | 4 | 5 | 2 | 5 | 6 | 2 | 7 | 8 | | 7 |
| *EcoR*Ⅰ+3 | h | g | i | | c | b | d | j | g | g | d |  |  | |  |
| *Hpa*Ⅱ/*Msp*Ⅰ+3 | 5 | 3 | 6 | | 7 | 5 | 6 | 6 | 7 | 6 | 3 |  |  | |  |

**TABLE S4** Primer combinations, number of bands obtained in the size range of 150-500 base pairs, and polymorphic band and scoring error rate information in the amplified fragment length polymorphism (AFLP) analysis

| Primer combinations | Total bands (150-  500 bp) | | Polymorphic bands^1^ | Polymorphic rate  (%) | Scoring error rate (%)^2^ | Polymorphic bands at the 5% level | Polymorphic rate  at the 5% level (%) |
| --- | --- | --- | --- | --- | --- | --- | --- |
| 4c | 157 | 156 | | 99.36 | 2.14 | 115 | 73.20 |
| 5i | 295 | 290 | | 98.31 | 3.31 | 222 | 75.30 |
| 4b | 138 | 138 | | 100 | 2.89 | 98 | 71.00 |
| 2i | 134 | 131 | | 97.76 | 2.80 | 99 | 73.90 |
| 5a | 203 | 197 | | 97.04 | 2.83 | 159 | 78.30 |
| 6j | 126 | 125 | | 99.21 | 1.80 | 90 | 71.40 |
| 2j | 213 | 208 | | 97.65 | 3.34 | 148 | 69.50 |
| 4i | 257 | 249 | | 96.89 | 3.70 | 215 | 83.70 |
| 7a | 113 | 110 | | 97.35 | 1.81 | 90 | 79.60 |
| 3c | 172 | 169 | | 98.26 | 2.70 | 113 | 65.70 |
| 7j | 187 | 182 | | 97.33 | 3.27 | 148 | 79.10 |
| 8j | 218 | 212 | | 97.25 | 2.96 | 178 | 81.70 |
| 7i | 265 | 258 | | 97.36 | 3.68 | 199 | 75.10 |
| Mean | 190.62 | 186.54 | | 97.98 | 2.935 | 144.15 | 75.19 |
| Sum | 2478 | 2425 | |  |  | 1874 |  |

Note: ^1^A locus was considered polymorphic if at least one individual in the sample showed a variant score.

^2^Calculated from the 6 individual plants that were reassayed as 100×(number of discordant scores in two independent analyses)/(number of scored markers×number of individuals).

**TABLE S5** Primer combinations, number of markers obtained in the size range of 150-500 base pairs, and polymorphic band and scoring error rate information in the methylation-sensitive amplified polymorphism (MSAP) analysis

| Primer combinations | Total bands  (150-500 bp) | Methylation-susceptible loci  (MSL) | | | Nonmethylated loci  (NML) | | | Scoring error rate (%) | | |
| --- | --- | --- | --- | --- | --- | --- | --- | --- | --- | --- |
|  |  | NL^1^ | PL^2^ | PPL  (%)^2^ | NL | PL | PPL  (%) | *Hpa*Ⅱ^3^ | *Msp*Ⅰ^3^ | *Hpa*Ⅱ  -*Msp*Ⅰ  Mismatch^4^ |
| 3g | 276 | 169 | 152 | 90 | 107 | 99 | 93 | 2.65 | 2.56 | 0.0507 |
| 6i | 205 | 107 | 91 | 85 | 98 | 71 | 72 | 2.56 | 2.8 | 0.0522 |
| 7c | 238 | 164 | 140 | 85 | 74 | 72 | 97 | 1.74 | 2.01 | 0.0368 |
| 5b | 388 | 276 | 239 | 87 | 112 | 105 | 94 | 2.84 | 3.05 | 0.0572 |
| 6d | 321 | 186 | 151 | 81 | 135 | 126 | 93 | 2.85 | 2.62 | 0.0532 |
| 6j | 332 | 233 | 208 | 89 | 99 | 91 | 92 | 3.14 | 3.36 | 0.0629 |
| 7g | 245 | 157 | 137 | 87 | 88 | 84 | 95 | 1.39 | 2.51 | 0.0383 |
| 6g | 267 | 173 | 140 | 81 | 94 | 86 | 91 | 2.13 | 2.66 | 0.0468 |
| 3d | 322 | 221 | 188 | 85 | 101 | 98 | 97 | 2.37 | 1.98 | 0.0426 |
| 5h | 290 | 201 | 183 | 91% | 89 | 77 | 87 | 3.44 | 3.18 | 0.064 |
| Mean | 288.4 | 188.7 | 162.9 | 86 | 99.7 | 90.9 | 91 | 2.511 | 2.673 | 0.05047 |
| Sum | 2884 | 1887 | 1629 | 86 | 997 | 909 | 91 |  |  |  |

Note: ^1^NL: Number of loci.

^2^PL: Number of polymorphic loci, PPL: Percentage of polymorphic loci. A locus was considered polymorphic if at least one individual in the sample showed a variant score.

^3^Calculated from the 6 individual plants that were reasassayed as 100×(number of discordant scores in two independent analyses)/(number of scored markers×number of individuals).

^4^*Hpa*Ⅱ-*Msp*Ⅰ Mismatch: Estimated probability of erroneous *Hpa*Ⅱ-*Msp*Ⅰ mismatches. Estimated average probability of obtaining discordant *EcoR*I-*Hpa*II and *EcoR*I-*Msp*I scores estimated from the scoring error rates for *EcoR*I-*Hpa*II (=e*Hpa*) and *EcoR*I-*Msp*I (=e*Msp*) as e*Hpa*+e*Msp*-2e*Hpa*e*Msp*.

**TABLE S6** Estimates of the genetic diversity of *Phragmites australis* from four habitats.

| Habitat | na | ne | H_E_ | I | *P* (%) |
| --- | --- | --- | --- | --- | --- |
| At the habitat level |  |  |  |  |  |
| H1 | 1.878±0.007 | 1.423±0.007 | 0.261±0.003 | 0.404±0.004 | 87.74 |
| H2 | 1.820±0.008 | 1.405±0.007 | 0.247±0.003 | 0.381±0.005 | 82.07 |
| H3 | 1.831±0.008 | 1.410±0.007 | 0.250±0.003 | 0.386±0.005 | 83.90 |
| H4 | 1.743±0.009 | 1.345±0.007 | 0.213±0.004 | 0.332±0.005 | 74.38 |
| N_m_=4.363 |  | | | | |
| At the population level | | | | | |
| H1 | 1.628 | 1.383 | 0.228 | 0.341 | 62.81 |
| H2 | 1.567 | 1.348 | 0.206 | 0.309 | 56.70 |
| H3 | 1.591 | 1.365 | 0.216 | 0.324 | 59.14 |
| H4 | 1.438 | 1.264 | 0.158 | 0.237 | 43.81 |

Note: The values of na, ne, H_E_, and I are indicated as the means±SEs; *P*, percentage of all loci that are polymorphic regardless of allele frequencies; Nm= estimate of gene flow from G_ST_, e.g., Nm= 0.5 (1-G_ST_) / G_ST_.

**TABLE** **S7** Estimates of epigenetic diversity and methylation levels of *Phragmites australis* from four habitats.

|  | na | ne | H_E_ | I | *P* (%) | Methylation levels | | | |
| --- | --- | --- | --- | --- | --- | --- | --- | --- | --- |
|  |  |  |  |  |  | Non-methylated | Hemimethylated | internal cytosine methylation | full methylation or absence of target |
| H1 | 1.820±  0.007 | 1.39±  0.006 | 0.24±  0.003 | 0.374±  0.004 | 82.09 | 0.1687 | 0.1140 | 0.1525 | 0.5647 |
| H2 | 1.812±  0.007 | 1.39±  0.006 | 0.24±  0.003 | 0.376±  0.004 | 81.27 | 0.1772 | 0.1117 | 0.1601 | 0.5511 |
| H3 | 1.816±  0.007 | 1.39±  0.006 | 0.24±  0.003 | 0.378±  0.004 | 81.67 | 0.1814 | 0.1168 | 0.1567 | 0.5451 |
| H4 | 1.790±  0.008 | 1.38±  0.006 | 0.23±  0.003 | 0.363±  0.005 | 79.10 | 0.1773 | 0.1174 | 0.1591 | 0.5463 |

Note: The values of na, ne, H_E_, and I are the means±SEs; *P*, percentage of all loci that are polymorphic regardless of allele frequencies.

**TABLE S8** ANOVA results for the field survey experiment concerning *Phragmites australis* in four different habitats (F value, * *P*<0.05, ** *P*<0.01)

|  | Habitat (df=3,8) | Location (df=8,348) |
| --- | --- | --- |
| Height (cm) | 2132.516^**^ | 0.111 |
| Stem diameter (cm) | 493.337^**^ | 0.243 |
| Leaf length (cm) | 330.304^**^ | 0.584 |
| Leaf width (cm) | 59.821^**^ | 0.616 |
| Node number | 26.568^**^ | 0.722 |
| Internode length (cm) | 349.761^**^ | 0.434 |
| Stem biomass (g) | 169.265^**^ | 0.347 |
| Leaf biomass (g) | 1211.249^**^ | 0.068 |
| Leaf sheath biomass (g) | 195.001^**^ | 0.305 |
| Total biomass (g) | 364.086^**^ | 0.200 |
| Stem fraction (%) | 66.175^**^ | 0.415 |
| Leaf fraction (%) | 32.143^**^ | 0.734 |
| Leaf sheath fraction (%) | 20.259^**^ | 1.321 |

**TABLE S9** List of measured soil factors and their contribution to AFLP variation as determined by forward variable selection with RDA

| Variables | R^2^ | F | *P* value | *P* value adjusted |
| --- | --- | --- | --- | --- |
| Ca^2+^ | 0.044 | 3.596 | 0.001 | 0.014 |
| Content of total N | 0.046 | 3.917 | 0.001 | 0.014 |
| Content of total P | 0.019 | 1.644 | 0.001 | 0.014 |
| Organic matter | 0.019 | 1.623 | 0.001 | 0.014 |
| Mg^2+^ | 0.019 | 1.653 | 0.001 | 0.014 |
| Conductivity | 0.020 | 1.761 | 0.001 | 0.014 |
| NO_3_-N | 0.022 | 1.929 | 0.001 | 0.014 |
| Na^+^ | 0.021 | 1.874 | 0.001 | 0.014 |
| K^+^ | 0.016 | 1.406 | 0.004 | 0.024 |
| pH | 0.014 | 1.265 | 0.012 | 0.060 |

Note: Significance in forward selection was determined by permutation tests and adjusted by Holm’s correction.

**TABLE S10** Result of the outlier loci of *Phragmites australis* in the 4 habitats obtained with Arlequin 3.5, with 35, 24 outliers showing higher F_ST_ values than expected under neutrality at the 99%, 99.5% probability level, respectively.

| Locus | Heterozygosity | F_ST_ | F_ST_ *P-*value |
| --- | --- | --- | --- |
| 116 | 0.3 | 0.57894737 | 0.00360047 |
| 183 | 0.49 | 0.3877551 | 0.00718083 |
| 362 | 0.35 | 0.68421053 | 0.00004017 |
| 466 | 0.48166667 | 0.40174832 | 0.00570665 |
| 500 | 0.275 | 0.52631579 | 0.00485478 |
| 501 | 0.33916667 | 0.57325747 | 0.00363695 |
| 502 | 0.44 | 0.4527512 | 0.00177561 |
| 636 | 0.3675 | 0.49158611 | 0.00949873 |
| 645 | 0.3 | 0.57894737 | 0.00360047 |
| 716 | 0.4925 | 0.40689287 | 0.00550693 |
| 945 | 0.50333333 | 0.48762635 | 0.00185249 |
| 1050 | 0.25 | 0.47368421 | 0.00657257 |
| 1062 | 0.45916667 | 0.40108893 | 0.00623189 |
| 1211 | 0.25 | 0.47368421 | 0.00657257 |
| 1246 | 0.425 | 0.84210526 | 0.0000001 |
| 1263 | 0.57 | 0.57756233 | 0.0000001 |
| 1482 | 0.57416667 | 0.57375296 | 0.0000001 |
| 1620 | 0.45833333 | 0.60095694 | 0.0001183 |
| 1644 | 0.51833333 | 0.46183787 | 0.00380608 |
| 1657 | 0.46833333 | 0.38190672 | 0.00885195 |
| 1695 | 0.5075 | 0.44775732 | 0.00356751 |
| 1819 | 0.5075 | 0.44775732 | 0.00356751 |
| 1881 | 0.42083333 | 0.53413236 | 0.0014534 |
| 1900 | 0.49333333 | 0.4905761 | 0.00146496 |
| 1917 | 0.505 | 0.57529964 | 0.0006622 |
| 1922 | 0.4325 | 0.38850015 | 0.00801688 |
| 1939 | 0.25 | 0.47368421 | 0.00657257 |
| 1954 | 0.4525 | 0.44751381 | 0.00205435 |
| 1957 | 0.45833333 | 0.60095694 | 0.0001183 |
| 1961 | 0.5375 | 0.59363525 | 0.0000001 |
| 1973 | 0.55083333 | 0.57480691 | 0.0000001 |
| 2139 | 0.56583333 | 0.55119758 | 0.00328815 |
| 2163 | 0.435 | 0.65214761 | 0.0000001 |
| 2164 | 0.42416667 | 0.4354255 | 0.00320005 |
| 2228 | 0.4825 | 0.40823561 | 0.00507182 |

**TABLE S11** Results of the outlier loci in *Phragmites australis* from 4 habitats obtained with BayeScan 2.0, which identified 18 candidate loci under selection on Jeffrey’s scale.

| Locus | probability | log10(PO) | F_ST_ |
| --- | --- | --- | --- |
| 372 | 0.92238 | 1.075 | 0.21904 |
| 862 | 0.9996 | 3.3977 | 0.29585 |
| 945 | 0.93459 | 1.155 | 0.21928 |
| 1246 | 0.86917 | 0.82241 | 0.21152 |
| 1248 | 0.87958 | 0.86356 | 0.20312 |
| 1263 | 0.997 | 2.5215 | 0.28871 |
| 1482 | 0.97079 | 1.5217 | 0.24027 |
| 1834 | 0.9994 | 3.2215 | 0.29465 |
| 1864 | 0.9776 | 1.6398 | 0.24966 |
| 1899 | 1 | 1000 | 0.36332 |
| 1900 | 0.96039 | 1.3847 | 0.24765 |
| 1917 | 0.999 | 2.9995 | 0.30543 |
| 1940 | 0.82577 | 0.67572 | 0.18463 |
| 1957 | 0.9952 | 2.3166 | 0.29629 |
| 1961 | 0.9982 | 2.7439 | 0.29709 |
| 1962 | 1 | 1000 | 0.32103 |
| 1973 | 0.9996 | 3.3977 | 0.29545 |
| 2139 | 0.94939 | 1.2732 | 0.22305 |

**TABLE S12** DistLM analysis testing for, first, a relationship between epigenetic differences among populations in the form of a resemblance matrix and the allelic frequencies in each population for all the AFLP loci ('Population-level analysis') and, second, a relationship between individual epigenotypes and genotypes across 80 individual plants ('Individual-level analysis'). The 675 and 1208 loci of the total 2446 non-outliers which revealed significant or marginally significant *P*-values and q-values at the population and individual level are shown, respectively.

|  | Population-level analysis (n=12) | | | |  | Individual-level analysis (n=80) | | |
| --- | --- | --- | --- | --- | --- | --- | --- | --- |
| AFLP  non-outlier locus | Pseudo-F | *P*-value | q-value |  | AFLP  non-outlier  locus | Pseudo-F | *P*-value | q-value |
| M3 | 7.1496 | 0.0131 | 0.0328717 |  | M1 | 1.3128 | 0.000700 | 0.002736 |
| M4 | 15.638 | 0.0097 | 0.029361 |  | M2 | 1.0923 | 0.078100 | 0.085331 |
| M10 | 2.6912 | 0.0671 | 0.0813123 |  | M3 | 1.4179 | 0.000100 | 0.000543 |
| M16 | 2.8607 | 0.0628 | 0.0802358 |  | M4 | 2.113 | 0.000010 | 0.000099 |
| M20 | 10.495 | 0.0078 | 0.0284595 |  | M7 | 1.1094 | 0.060100 | 0.070986 |
| M21 | 13.46 | 0.0046 | 0.02538 |  | M9 | 1.1085 | 0.054800 | 0.067092 |
| M22 | 5.2794 | 0.0243 | 0.0463347 |  | M10 | 1.2453 | 0.003300 | 0.009026 |
| M27 | 3.9447 | 0.0437 | 0.0668905 |  | M12 | 1.3642 | 0.000300 | 0.001392 |
| M35 | 3.5436 | 0.0554 | 0.0755455 |  | M13 | 1.4971 | 0.000020 | 0.000171 |
| M36 | 5.8989 | 0.0155 | 0.0355743 |  | M15 | 1.1787 | 0.012300 | 0.024037 |
| M38 | 2.9257 | 0.0873 | 0.0913605 |  | M16 | 1.1704 | 0.014900 | 0.026990 |
| M40 | 3.5982 | 0.056 | 0.0759036 |  | M18 | 1.1254 | 0.041100 | 0.054874 |
| M44 | 3.3868 | 0.0557 | 0.0758014 |  | M20 | 1.8596 | 0.000010 | 0.000099 |
| M49 | 6.8622 | 0.0095 | 0.0292808 |  | M21 | 1.8762 | 0.000010 | 0.000099 |
| M60 | 3.9371 | 0.0455 | 0.0680987 |  | M22 | 1.3104 | 0.000600 | 0.002383 |
| M62 | 12.248 | 0.0006 | 0.02 |  | M27 | 1.3 | 0.000900 | 0.003349 |
| M70 | 16.982 | 0.0002 | 0.016875 |  | M29 | 1.1052 | 0.060100 | 0.070986 |
| M76 | 3.7084 | 0.0442 | 0.0671959 |  | M32 | 1.1466 | 0.026900 | 0.040895 |
| M77 | 18.543 | 0.0008 | 0.02 |  | M33 | 1.1802 | 0.011600 | 0.023484 |
| M80 | 4.3246 | 0.0362 | 0.0603333 |  | M34 | 1.2786 | 0.001400 | 0.004650 |
| M84 | 3.2433 | 0.0654 | 0.0813123 |  | M35 | 1.364 | 0.000300 | 0.001392 |
| M85 | 2.9879 | 0.0841 | 0.0905383 |  | M36 | 1.3264 | 0.000500 | 0.002107 |
| M89 | 6.5092 | 0.0084 | 0.0286463 |  | M38 | 1.1782 | 0.011800 | 0.023811 |
| M91 | 4.0717 | 0.041 | 0.0651232 |  | M40 | 1.2399 | 0.003400 | 0.009258 |
| M92 | 3.6935 | 0.0574 | 0.0765533 |  | M42 | 1.091 | 0.079800 | 0.086502 |
| M101 | 2.9981 | 0.085 | 0.0909271 |  | M44 | 1.2626 | 0.001900 | 0.005900 |
| M109 | 12.94 | 0.0002 | 0.016875 |  | M46 | 1.1327 | 0.032800 | 0.046819 |
| M110 | 15.131 | 0.009 | 0.0286557 |  | M47 | 1.129 | 0.034500 | 0.048630 |
| M112 | 3.2138 | 0.0716 | 0.0839063 |  | M49 | 1.3015 | 0.001000 | 0.003602 |
| M113 | 5.433 | 0.0215 | 0.0437123 |  | M51 | 1.0938 | 0.077500 | 0.085051 |
| M114 | 7.0708 | 0.0086 | 0.0286463 |  | M53 | 1.1366 | 0.029900 | 0.044473 |
| M115 | 12.02 | 0.0065 | 0.0269172 |  | M58 | 1.0969 | 0.073200 | 0.081855 |
| M117 | 5.3184 | 0.022 | 0.0441964 |  | M60 | 1.4318 | 0.000090 | 0.000541 |
| M121 | 3.6701 | 0.0505 | 0.0716124 |  | M61 | 1.1144 | 0.048800 | 0.061950 |
| M126 | 2.8291 | 0.0857 | 0.0912988 |  | M62 | 1.6328 | 0.000010 | 0.000099 |
| M128 | 7.3907 | 0.0245 | 0.0464537 |  | M66 | 1.6398 | 0.000010 | 0.000099 |
| M131 | 8.0923 | 0.0015 | 0.0215426 |  | M69 | 1.1843 | 0.010600 | 0.022036 |
| M147 | 2.9904 | 0.0811 | 0.0887237 |  | M70 | 1.5489 | 0.000010 | 0.000099 |
| M148 | 23.413 | 0.0031 | 0.0247277 |  | M75 | 1.095 | 0.077600 | 0.085085 |
| M150 | 19.26 | 0.015 | 0.0352787 |  | M76 | 1.1752 | 0.013700 | 0.025797 |
| M151 | 8.8443 | 0.0048 | 0.0255118 |  | M77 | 1.9408 | 0.000010 | 0.000099 |
| M153 | 5.5994 | 0.0188 | 0.0401582 |  | M78 | 1.1619 | 0.017000 | 0.029377 |
| M158 | 3.7048 | 0.0532 | 0.0740412 |  | M79 | 1.3341 | 0.000500 | 0.002107 |
| M160 | 3.1209 | 0.0739 | 0.0849787 |  | M80 | 1.3113 | 0.000800 | 0.003059 |
| M161 | 3.5029 | 0.0572 | 0.0764554 |  | M81 | 1.1227 | 0.040600 | 0.054323 |
| M162 | 3.0633 | 0.0767 | 0.0863866 |  | M83 | 1.1876 | 0.024600 | 0.038679 |
| M164 | 2.6254 | 0.0947 | 0.0961824 |  | M84 | 1.4341 | 0.000050 | 0.000344 |
| M170 | 4.4284 | 0.0303 | 0.0532617 |  | M85 | 1.3177 | 0.000600 | 0.002383 |
| M172 | 3.5892 | 0.0726 | 0.084201 |  | M86 | 1.105 | 0.057300 | 0.069128 |
| M175 | 4.2468 | 0.0386 | 0.062482 |  | M88 | 1.2359 | 0.003400 | 0.009258 |
| M179 | 2.7815 | 0.0755 | 0.0856731 |  | M89 | 1.4106 | 0.000090 | 0.000541 |
| M181 | 3.9205 | 0.0438 | 0.0668905 |  | M90 | 1.0929 | 0.080400 | 0.086773 |
| M183 | 3.0601 | 0.0797 | 0.0880483 |  | M91 | 1.151 | 0.022300 | 0.035883 |
| M187 | 7.7247 | 0.0053 | 0.0257143 |  | M93 | 1.1681 | 0.015900 | 0.028103 |
| M188 | 3.3859 | 0.0673 | 0.0813123 |  | M95 | 1.1419 | 0.026600 | 0.040488 |
| M189 | 5.1724 | 0.0174 | 0.0378871 |  | M99 | 1.1104 | 0.056300 | 0.068254 |
| M190 | 4.0516 | 0.0434 | 0.0668836 |  | M101 | 1.2056 | 0.007100 | 0.016171 |
| M192 | 5.8591 | 0.0133 | 0.0328846 |  | M102 | 1.1518 | 0.021300 | 0.034816 |
| M194 | 5.4407 | 0.0242 | 0.0463347 |  | M103 | 1.0881 | 0.085100 | 0.089735 |
| M197 | 2.9843 | 0.0802 | 0.0881006 |  | M105 | 1.3063 | 0.001100 | 0.003872 |
| M200 | 4.5115 | 0.0318 | 0.0550385 |  | M106 | 1.1173 | 0.045800 | 0.059234 |
| M201 | 5.1131 | 0.0215 | 0.0437123 |  | M107 | 1.0877 | 0.089900 | 0.092899 |
| M203 | 8.9936 | 0.0055 | 0.0257143 |  | M108 | 1.1153 | 0.056200 | 0.068200 |
| M205 | 5.5241 | 0.0143 | 0.0344681 |  | M109 | 1.2543 | 0.002300 | 0.006850 |
| M211 | 5.8896 | 0.0239 | 0.0462249 |  | M110 | 1.6635 | 0.000010 | 0.000099 |
| M214 | 3.8787 | 0.0424 | 0.0659447 |  | M113 | 1.3303 | 0.000500 | 0.002107 |
| M217 | 6.0085 | 0.0117 | 0.0312353 |  | M114 | 1.3846 | 0.000200 | 0.000999 |
| M220 | 4.1613 | 0.0411 | 0.0651232 |  | M115 | 1.546 | 0.000010 | 0.000099 |
| M222 | 3.3712 | 0.0458 | 0.0682434 |  | M117 | 1.2925 | 0.001100 | 0.003872 |
| M223 | 14.167 | 0.0009 | 0.02025 |  | M121 | 1.1225 | 0.040900 | 0.054666 |
| M224 | 8.2306 | 0.0021 | 0.0225 |  | M122 | 1.4432 | 0.000100 | 0.000543 |
| M225 | 4.0912 | 0.0453 | 0.06795 |  | M123 | 1.1304 | 0.034000 | 0.048089 |
| M226 | 4.0467 | 0.0402 | 0.0643009 |  | M124 | 1.1501 | 0.024400 | 0.038512 |
| M227 | 10.449 | 0.0022 | 0.0225 |  | M125 | 1.1002 | 0.065700 | 0.075794 |
| M228 | 3.5323 | 0.0447 | 0.0675 |  | M126 | 1.1067 | 0.057100 | 0.068954 |
| M229 | 9.5256 | 0.0035 | 0.0247277 |  | M128 | 1.1181 | 0.048000 | 0.061311 |
| M231 | 4.5678 | 0.03 | 0.0528721 |  | M129 | 1.1227 | 0.040200 | 0.054021 |
| M235 | 6.8831 | 0.0152 | 0.0353793 |  | M130 | 1.0934 | 0.080700 | 0.086945 |
| M236 | 2.7267 | 0.0735 | 0.084663 |  | M131 | 1.1124 | 0.050400 | 0.063076 |
| M237 | 8.3754 | 0.0064 | 0.0269172 |  | M140 | 1.334 | 0.000600 | 0.002383 |
| M241 | 2.9851 | 0.0828 | 0.0898553 |  | M142 | 1.1189 | 0.044600 | 0.058291 |
| M242 | 7.8189 | 0.0047 | 0.02538 |  | M143 | 1.282 | 0.001400 | 0.004650 |
| M245 | 12.101 | 0.0129 | 0.0326124 |  | M145 | 1.1144 | 0.048900 | 0.062013 |
| M246 | 5.9262 | 0.011 | 0.0305556 |  | M147 | 1.1713 | 0.014000 | 0.026084 |
| M253 | 3.0837 | 0.0796 | 0.0880483 |  | M148 | 1.6548 | 0.000010 | 0.000099 |
| M254 | 3.8989 | 0.0475 | 0.0699946 |  | M150 | 2.0583 | 0.000010 | 0.000099 |
| M257 | 4.424 | 0.0484 | 0.0705616 |  | M151 | 1.4517 | 0.000060 | 0.000395 |
| M260 | 7.8037 | 0.01 | 0.0298673 |  | M153 | 1.1612 | 0.017500 | 0.029948 |
| M265 | 3.1865 | 0.0719 | 0.0839663 |  | M156 | 1.1949 | 0.008100 | 0.018050 |
| M270 | 21.718 | 0.0078 | 0.0284595 |  | M157 | 1.1919 | 0.012300 | 0.024037 |
| M271 | 18.895 | 0.0005 | 0.02 |  | M158 | 1.0898 | 0.081500 | 0.087276 |
| M273 | 3.448 | 0.0501 | 0.0716124 |  | M159 | 1.0883 | 0.084000 | 0.088954 |
| M277 | 8.4499 | 0.005 | 0.0257143 |  | M160 | 1.0878 | 0.086100 | 0.090328 |
| M279 | 6.186 | 0.013 | 0.0327425 |  | M161 | 1.2717 | 0.001700 | 0.005443 |
| M284 | 4.62 | 0.0261 | 0.0481352 |  | M162 | 1.1939 | 0.009100 | 0.019711 |
| M291 | 4.7743 | 0.0244 | 0.0463944 |  | M163 | 1.1394 | 0.031300 | 0.045464 |
| M293 | 4.2458 | 0.0367 | 0.0605685 |  | M166 | 1.1053 | 0.059800 | 0.070902 |
| M295 | 4.7936 | 0.0224 | 0.0444706 |  | M170 | 1.1355 | 0.031100 | 0.045386 |
| M301 | 4.6571 | 0.0325 | 0.0556789 |  | M172 | 1.0798 | 0.099700 | 0.099900 |
| M302 | 7.9344 | 0.0092 | 0.0288837 |  | M173 | 1.1704 | 0.014800 | 0.026887 |
| M303 | 9.4612 | 0.0056 | 0.0257143 |  | M174 | 1.1274 | 0.036000 | 0.049837 |
| M306 | 3.1762 | 0.0703 | 0.0828141 |  | M175 | 1.1809 | 0.011500 | 0.023320 |
| M307 | 3.1762 | 0.0698 | 0.0824869 |  | M178 | 1.1124 | 0.050000 | 0.062702 |
| M314 | 6.8815 | 0.0218 | 0.0439254 |  | M181 | 1.1161 | 0.047500 | 0.060798 |
| M316 | 2.8502 | 0.0933 | 0.0954205 |  | M183 | 1.2816 | 0.001500 | 0.004917 |
| M317 | 20.593 | 0.0002 | 0.016875 |  | M186 | 1.0855 | 0.092800 | 0.094946 |
| M323 | 4.9187 | 0.0222 | 0.0443343 |  | M187 | 1.207 | 0.006800 | 0.015719 |
| M324 | 19.746 | 0.0016 | 0.0216 |  | M189 | 1.1723 | 0.013800 | 0.025867 |
| M328 | 8.2486 | 0.0045 | 0.02538 |  | M191 | 1.0991 | 0.070700 | 0.079998 |
| M336 | 4.4256 | 0.032 | 0.055102 |  | M192 | 1.2498 | 0.003200 | 0.008830 |
| M343 | 4.2691 | 0.0384 | 0.06247 |  | M194 | 1.155 | 0.020400 | 0.033701 |
| M344 | 7.1968 | 0.0028 | 0.0247277 |  | M200 | 1.1708 | 0.013900 | 0.026015 |
| M350 | 2.6219 | 0.087 | 0.0913605 |  | M201 | 1.1477 | 0.023400 | 0.037075 |
| M355 | 5.4147 | 0.0119 | 0.031377 |  | M203 | 1.3691 | 0.000300 | 0.001392 |
| M356 | 16.368 | 0.0074 | 0.027905 |  | M204 | 1.1205 | 0.043900 | 0.057680 |
| M360 | 2.7634 | 0.0668 | 0.0813123 |  | M205 | 1.2516 | 0.002500 | 0.007237 |
| M364 | 5.4903 | 0.0132 | 0.0328846 |  | M208 | 1.0957 | 0.071900 | 0.080912 |
| M366 | 3.0982 | 0.0609 | 0.0787285 |  | M209 | 1.1184 | 0.045600 | 0.059037 |
| M367 | 14.619 | 0.0098 | 0.0294 |  | M211 | 1.264 | 0.002100 | 0.006377 |
| M368 | 4.7468 | 0.0206 | 0.0427846 |  | M212 | 1.1044 | 0.062100 | 0.072793 |
| M375 | 3.9824 | 0.0395 | 0.0636337 |  | M217 | 1.175 | 0.012200 | 0.024037 |
| M379 | 3.8717 | 0.0332 | 0.0565909 |  | M220 | 1.1603 | 0.017500 | 0.029948 |
| M380 | 5.3482 | 0.0165 | 0.0367574 |  | M221 | 1.1804 | 0.012400 | 0.024157 |
| M381 | 10.946 | 0.0087 | 0.0286463 |  | M222 | 1.2535 | 0.002500 | 0.007237 |
| M384 | 3.7352 | 0.0417 | 0.0654593 |  | M223 | 1.6531 | 0.000010 | 0.000099 |
| M385 | 3.1822 | 0.0437 | 0.0668905 |  | M224 | 1.3958 | 0.000200 | 0.000999 |
| M387 | 7.1935 | 0.0032 | 0.0247277 |  | M226 | 1.1709 | 0.015200 | 0.027294 |
| M388 | 4.3031 | 0.0369 | 0.06075 |  | M227 | 1.6036 | 0.000020 | 0.000171 |
| M391 | 2.7342 | 0.0788 | 0.0873399 |  | M231 | 1.2798 | 0.001500 | 0.004917 |
| M393 | 4.9498 | 0.0266 | 0.0486503 |  | M234 | 1.1838 | 0.010700 | 0.022207 |
| M398 | 23.973 | 0.0015 | 0.0215426 |  | M235 | 1.2042 | 0.007000 | 0.016002 |
| M400 | 6.2871 | 0.0123 | 0.0318103 |  | M236 | 1.2438 | 0.003000 | 0.008372 |
| M401 | 17.025 | 0.0045 | 0.02538 |  | M237 | 1.267 | 0.001800 | 0.005646 |
| M402 | 4.6834 | 0.0314 | 0.0547674 |  | M238 | 1.1233 | 0.039600 | 0.053389 |
| M403 | 10.377 | 0.0074 | 0.027905 |  | M241 | 1.1743 | 0.013700 | 0.025797 |
| M408 | 7.0968 | 0.0216 | 0.0437838 |  | M242 | 1.4019 | 0.000080 | 0.000496 |
| M410 | 2.7501 | 0.0629 | 0.0802358 |  | M245 | 1.602 | 0.000010 | 0.000099 |
| M413 | 2.4648 | 0.0821 | 0.0893831 |  | M246 | 1.1446 | 0.025000 | 0.038962 |
| M416 | 3.282 | 0.0559 | 0.0759036 |  | M250 | 1.1771 | 0.012800 | 0.024626 |
| M417 | 2.9457 | 0.0601 | 0.0784459 |  | M253 | 1.1348 | 0.030800 | 0.045001 |
| M418 | 20.015 | 0.0031 | 0.0247277 |  | M254 | 1.0905 | 0.079800 | 0.086502 |
| M425 | 4.583 | 0.0236 | 0.0459078 |  | M257 | 1.2193 | 0.005000 | 0.012341 |
| M430 | 9.2791 | 0.0027 | 0.0247277 |  | M260 | 1.3473 | 0.000400 | 0.001764 |
| M433 | 6.7301 | 0.0083 | 0.0286463 |  | M261 | 1.1113 | 0.052500 | 0.064983 |
| M434 | 4.6587 | 0.0345 | 0.0583647 |  | M269 | 1.1085 | 0.055200 | 0.067449 |
| M436 | 4.998 | 0.0209 | 0.0431422 |  | M270 | 1.9558 | 0.000010 | 0.000099 |
| M447 | 4.5973 | 0.0425 | 0.0659483 |  | M271 | 1.2382 | 0.003700 | 0.009859 |
| M453 | 5.2165 | 0.0271 | 0.0486503 |  | M273 | 1.1211 | 0.042100 | 0.055669 |
| M462 | 4.2621 | 0.0385 | 0.06247 |  | M274 | 1.1052 | 0.062500 | 0.073054 |
| M466 | 14.437 | 0.000040 | 0.0135 |  | M276 | 1.0867 | 0.089200 | 0.092484 |
| M467 | 7.1088 | 0.0094 | 0.0291055 |  | M277 | 1.2831 | 0.001300 | 0.004377 |
| M474 | 6.0177 | 0.0085 | 0.0286463 |  | M279 | 1.2057 | 0.006900 | 0.015832 |
| M475 | 3.2747 | 0.0687 | 0.0818541 |  | M281 | 1.1039 | 0.059200 | 0.070528 |
| M481 | 2.9601 | 0.0772 | 0.0863866 |  | M284 | 1.1092 | 0.052900 | 0.065282 |
| M482 | 22.886 | 0.0016 | 0.0216 |  | M286 | 1.0888 | 0.089100 | 0.092458 |
| M494 | 4.3215 | 0.0348 | 0.058725 |  | M287 | 1.1548 | 0.020700 | 0.034105 |
| M495 | 4.6804 | 0.0186 | 0.0399841 |  | M288 | 1.2783 | 0.001300 | 0.004377 |
| M497 | 2.3808 | 0.0959 | 0.0967164 |  | M289 | 1.096 | 0.070900 | 0.080078 |
| M499 | 4.0504 | 0.0381 | 0.06227 |  | M291 | 1.2012 | 0.008000 | 0.017892 |
| M503 | 4.2204 | 0.0365 | 0.0605685 |  | M293 | 1.0987 | 0.066000 | 0.076069 |
| M507 | 4.7562 | 0.0271 | 0.0486503 |  | M294 | 1.1018 | 0.064000 | 0.074178 |
| M510 | 10.685 | 0.0023 | 0.0231716 |  | M295 | 1.4028 | 0.000100 | 0.000543 |
| M513 | 13.523 | 0.0041 | 0.0252391 |  | M297 | 1.1158 | 0.045600 | 0.059037 |
| M518 | 16.983 | 0.0063 | 0.0269146 |  | M298 | 1.0936 | 0.075800 | 0.083929 |
| M519 | 3.9704 | 0.0477 | 0.0699946 |  | M299 | 1.2045 | 0.006600 | 0.015313 |
| M520 | 6.2913 | 0.0171 | 0.0378443 |  | M301 | 1.2836 | 0.001400 | 0.004650 |
| M523 | 3.323 | 0.0575 | 0.0765533 |  | M302 | 1.4459 | 0.000060 | 0.000395 |
| M527 | 2.9874 | 0.0591 | 0.0774612 |  | M303 | 1.6414 | 0.000020 | 0.000171 |
| M528 | 28.183 | 0.0001 | 0.016875 |  | M304 | 1.2228 | 0.004500 | 0.011332 |
| M530 | 11.087 | 0.0008 | 0.02 |  | M309 | 1.1526 | 0.021300 | 0.034816 |
| M533 | 10.396 | 0.0003 | 0.016875 |  | M311 | 1.2729 | 0.001800 | 0.005646 |
| M537 | 7.9214 | 0.0105 | 0.0301596 |  | M312 | 1.3054 | 0.001000 | 0.003602 |
| M551 | 2.344 | 0.0628 | 0.0802358 |  | M313 | 1.3209 | 0.000800 | 0.003059 |
| M552 | 4.1065 | 0.0366 | 0.0605685 |  | M314 | 1.4555 | 0.000060 | 0.000395 |
| M563 | 4.4504 | 0.0271 | 0.0486503 |  | M317 | 1.9168 | 0.000010 | 0.000099 |
| M565 | 13.589 | 0.0118 | 0.0312353 |  | M323 | 1.611 | 0.000010 | 0.000099 |
| M567 | 3.8508 | 0.0421 | 0.0656293 |  | M324 | 1.7847 | 0.000010 | 0.000099 |
| M569 | 2.5482 | 0.0855 | 0.0912988 |  | M326 | 1.1607 | 0.018500 | 0.031186 |
| M570 | 4.9909 | 0.0195 | 0.0413915 |  | M328 | 1.4644 | 0.000040 | 0.000295 |
| M575 | 14.554 | 0.0021 | 0.0225 |  | M331 | 1.1227 | 0.041600 | 0.055244 |
| M580 | 3.4843 | 0.0552 | 0.0755455 |  | M336 | 1.2356 | 0.003900 | 0.010238 |
| M583 | 6.1604 | 0.0162 | 0.03645 |  | M343 | 1.2263 | 0.004200 | 0.010752 |
| M587 | 3.4281 | 0.0439 | 0.0668905 |  | M345 | 1.1003 | 0.066800 | 0.076634 |
| M588 | 2.5031 | 0.0949 | 0.0961824 |  | M350 | 1.1128 | 0.049400 | 0.062392 |
| M590 | 7.4171 | 0.0152 | 0.0353793 |  | M355 | 1.2157 | 0.005700 | 0.013767 |
| M596 | 10.628 | 0.0003 | 0.016875 |  | M356 | 1.5599 | 0.000050 | 0.000344 |
| M598 | 4.5902 | 0.0342 | 0.0581486 |  | M358 | 1.1583 | 0.018600 | 0.031269 |
| M601 | 3.4389 | 0.061 | 0.0787285 |  | M363 | 1.0999 | 0.068400 | 0.078151 |
| M606 | 8.6104 | 0.0035 | 0.0247277 |  | M365 | 1.0853 | 0.092000 | 0.094517 |
| M607 | 6.8709 | 0.0079 | 0.028516 |  | M366 | 1.2332 | 0.003600 | 0.009634 |
| M609 | 16.549 | 0.0042 | 0.0252391 |  | M367 | 1.8933 | 0.000010 | 0.000099 |
| M613 | 3.6645 | 0.0493 | 0.0711058 |  | M368 | 1.087 | 0.087800 | 0.091569 |
| M614 | 8.5761 | 0.0055 | 0.0257143 |  | M370 | 1.0837 | 0.095300 | 0.096864 |
| M616 | 3.2701 | 0.0658 | 0.0813123 |  | M373 | 1.0954 | 0.075200 | 0.083488 |
| M630 | 6.2086 | 0.0156 | 0.0355743 |  | M374 | 1.1361 | 0.031900 | 0.045958 |
| M633 | 7.478 | 0.0036 | 0.0247277 |  | M377 | 1.1333 | 0.032100 | 0.046086 |
| M635 | 26.633 | 0.0005 | 0.02 |  | M379 | 1.2241 | 0.004600 | 0.011537 |
| M636 | 23.155 | 0.0021 | 0.0225 |  | M380 | 1.2698 | 0.001700 | 0.005443 |
| M637 | 5.6604 | 0.0213 | 0.0437123 |  | M381 | 1.4942 | 0.000040 | 0.000295 |
| M646 | 4.5002 | 0.029 | 0.0512435 |  | M384 | 1.1601 | 0.018200 | 0.030933 |
| M648 | 15.867 | 0.0031 | 0.0247277 |  | M385 | 1.2611 | 0.002200 | 0.006616 |
| M651 | 3.3303 | 0.0669 | 0.0813123 |  | M386 | 1.1365 | 0.029500 | 0.044037 |
| M657 | 3.4198 | 0.0651 | 0.0813123 |  | M387 | 1.188 | 0.009900 | 0.021040 |
| M663 | 10.501 | 0.0029 | 0.0247277 |  | M388 | 1.1333 | 0.031800 | 0.045958 |
| M665 | 4.1022 | 0.0667 | 0.0813123 |  | M389 | 1.257 | 0.002300 | 0.006850 |
| M668 | 2.9217 | 0.0873 | 0.0913605 |  | M391 | 1.2672 | 0.001800 | 0.005646 |
| M671 | 10.575 | 0.0021 | 0.0225 |  | M393 | 1.2512 | 0.002900 | 0.008148 |
| M675 | 15.739 | 0.011 | 0.0305556 |  | M394 | 1.4557 | 0.000100 | 0.000543 |
| M680 | 5.9262 | 0.0155 | 0.0355743 |  | M398 | 2.1614 | 0.000010 | 0.000099 |
| M681 | 3.334 | 0.069 | 0.0818541 |  | M399 | 1.1759 | 0.012100 | 0.023987 |
| M685 | 16.305 | 0.0007 | 0.02 |  | M400 | 1.2923 | 0.001300 | 0.004377 |
| M690 | 17.597 | 0.0047 | 0.02538 |  | M401 | 1.7911 | 0.000010 | 0.000099 |
| M693 | 23.429 | 0.0074 | 0.027905 |  | M402 | 1.2524 | 0.002700 | 0.007726 |
| M698 | 2.5763 | 0.0892 | 0.0922052 |  | M403 | 1.6316 | 0.000010 | 0.000099 |
| M699 | 9.6709 | 0.0014 | 0.0215426 |  | M406 | 1.1676 | 0.015200 | 0.027294 |
| M703 | 5.3686 | 0.0182 | 0.039375 |  | M407 | 1.096 | 0.078000 | 0.085331 |
| M710 | 6.1901 | 0.0184 | 0.0396805 |  | M408 | 1.3673 | 0.000400 | 0.001764 |
| M712 | 20.269 | 0.0083 | 0.0286463 |  | M411 | 1.1052 | 0.062300 | 0.072958 |
| M714 | 3.4251 | 0.0659 | 0.0813123 |  | M413 | 1.1734 | 0.014200 | 0.026248 |
| M717 | 2.4875 | 0.0903 | 0.0931995 |  | M416 | 1.0944 | 0.076500 | 0.084252 |
| M718 | 2.3305 | 0.0671 | 0.0813123 |  | M417 | 1.1683 | 0.015400 | 0.027533 |
| M719 | 5.337 | 0.0156 | 0.0355743 |  | M418 | 1.3554 | 0.000500 | 0.002107 |
| M720 | 3.1787 | 0.0563 | 0.0759543 |  | M419 | 1.1178 | 0.046900 | 0.060404 |
| M722 | 2.9245 | 0.0758 | 0.0856731 |  | M421 | 1.3364 | 0.000500 | 0.002107 |
| M723 | 3.985 | 0.0502 | 0.0716124 |  | M425 | 1.3501 | 0.000400 | 0.001764 |
| M724 | 4.1826 | 0.0358 | 0.0599628 |  | M427 | 1.0912 | 0.081300 | 0.087276 |
| M726 | 3.9597 | 0.049 | 0.0711058 |  | M429 | 1.1651 | 0.015400 | 0.027533 |
| M728 | 9.1103 | 0.0015 | 0.0215426 |  | M430 | 1.4649 | 0.000050 | 0.000344 |
| M733 | 4.1465 | 0.0385 | 0.06247 |  | M431 | 1.1114 | 0.051400 | 0.064005 |
| M735 | 2.4577 | 0.0866 | 0.0912988 |  | M433 | 1.1257 | 0.038100 | 0.051875 |
| M740 | 6.4011 | 0.006 | 0.0259615 |  | M434 | 1.1744 | 0.013200 | 0.025123 |
| M741 | 4.9476 | 0.0257 | 0.048054 |  | M436 | 1.1275 | 0.037500 | 0.051283 |
| M743 | 6.1557 | 0.0122 | 0.0317954 |  | M439 | 1.3509 | 0.000400 | 0.001764 |
| M746 | 12.37 | 0.0067 | 0.027244 |  | M440 | 1.4583 | 0.000090 | 0.000541 |
| M747 | 19.504 | 0.0015 | 0.0215426 |  | M441 | 1.0874 | 0.085100 | 0.089735 |
| M748 | 10.419 | 0.0006 | 0.02 |  | M442 | 1.1156 | 0.047800 | 0.061119 |
| M753 | 3.3202 | 0.0657 | 0.0813123 |  | M445 | 1.3107 | 0.000700 | 0.002736 |
| M755 | 12.668 | 0.001 | 0.0210938 |  | M447 | 1.2862 | 0.001200 | 0.004142 |
| M756 | 4.5115 | 0.0367 | 0.0605685 |  | M450 | 1.3097 | 0.000900 | 0.003349 |
| M759 | 8.7367 | 0.0138 | 0.0335072 |  | M451 | 1.1316 | 0.033400 | 0.047457 |
| M764 | 5.0462 | 0.0214 | 0.0437123 |  | M453 | 1.2453 | 0.003000 | 0.008372 |
| M765 | 8.7176 | 0.0106 | 0.0301899 |  | M454 | 1.2435 | 0.002900 | 0.008148 |
| M766 | 2.7578 | 0.0881 | 0.0919127 |  | M455 | 1.1731 | 0.013400 | 0.025386 |
| M771 | 3.258 | 0.063 | 0.0802358 |  | M456 | 1.1634 | 0.016500 | 0.028753 |
| M777 | 4.9921 | 0.027 | 0.0486503 |  | M458 | 1.0844 | 0.092500 | 0.094819 |
| M787 | 2.6043 | 0.0988 | 0.0989466 |  | M459 | 1.1082 | 0.055800 | 0.068047 |
| M789 | 3.2043 | 0.0709 | 0.0832304 |  | M461 | 1.0857 | 0.091300 | 0.093954 |
| M808 | 12.768 | 0.0133 | 0.0328846 |  | M462 | 1.22 | 0.004900 | 0.012191 |
| M810 | 4.7615 | 0.027 | 0.0486503 |  | M465 | 1.1526 | 0.020600 | 0.033986 |
| M811 | 4.2214 | 0.0374 | 0.0614234 |  | M466 | 1.9064 | 0.000010 | 0.000099 |
| M821 | 3.1551 | 0.0773 | 0.0863866 |  | M467 | 1.6795 | 0.000010 | 0.000099 |
| M823 | 4.878 | 0.0242 | 0.0463347 |  | M470 | 1.2417 | 0.002900 | 0.008148 |
| M825 | 3.5005 | 0.0635 | 0.080243 |  | M473 | 1.1245 | 0.039300 | 0.053100 |
| M832 | 3.5556 | 0.0585 | 0.0774265 |  | M474 | 1.3369 | 0.000400 | 0.001764 |
| M838 | 18.319 | 0.0024 | 0.0238235 |  | M475 | 1.2993 | 0.000900 | 0.003349 |
| M849 | 4.5059 | 0.0269 | 0.0486503 |  | M476 | 1.2898 | 0.001200 | 0.004142 |
| M852 | 4.2472 | 0.0604 | 0.0785549 |  | M479 | 1.0926 | 0.078500 | 0.085618 |
| M854 | 3.049 | 0.0815 | 0.089017 |  | M482 | 1.8764 | 0.000010 | 0.000099 |
| M855 | 3.7777 | 0.0484 | 0.0705616 |  | M483 | 1.4345 | 0.000090 | 0.000541 |
| M857 | 5.352 | 0.0111 | 0.030707 |  | M484 | 1.1051 | 0.059800 | 0.070902 |
| M863 | 2.7518 | 0.0992 | 0.0992 |  | M486 | 1.1734 | 0.013800 | 0.025867 |
| M866 | 6.8222 | 0.0108 | 0.030375 |  | M487 | 1.0823 | 0.099800 | 0.099900 |
| M867 | 5.4628 | 0.0173 | 0.0378871 |  | M488 | 1.0836 | 0.097900 | 0.098536 |
| M875 | 5.6378 | 0.0151 | 0.0353793 |  | M494 | 1.1518 | 0.021900 | 0.035423 |
| M885 | 3.016 | 0.0866 | 0.0912988 |  | M496 | 1.2075 | 0.007300 | 0.016505 |
| M893 | 2.2858 | 0.0849 | 0.0909271 |  | M498 | 1.0865 | 0.088600 | 0.092248 |
| M897 | 2.6208 | 0.0928 | 0.0951976 |  | M499 | 1.434 | 0.000050 | 0.000344 |
| M898 | 2.7968 | 0.0969 | 0.0973326 |  | M507 | 1.2921 | 0.001000 | 0.003602 |
| M899 | 17.448 | 0.0002 | 0.016875 |  | M509 | 1.1826 | 0.010500 | 0.021865 |
| M904 | 5.4391 | 0.0135 | 0.0330163 |  | M510 | 1.688 | 0.000010 | 0.000099 |
| M917 | 2.6974 | 0.0625 | 0.0802358 |  | M513 | 1.6839 | 0.000010 | 0.000099 |
| M923 | 2.6077 | 0.0709 | 0.0832304 |  | M514 | 1.1295 | 0.035800 | 0.049727 |
| M928 | 3.9418 | 0.0459 | 0.0682434 |  | M515 | 1.2553 | 0.002500 | 0.007237 |
| M930 | 14.186 | 0.0014 | 0.0215426 |  | M517 | 1.277 | 0.001700 | 0.005443 |
| M931 | 17.539 | 0.0058 | 0.0257566 |  | M518 | 1.9039 | 0.000010 | 0.000099 |
| M934 | 4.9753 | 0.0241 | 0.0463347 |  | M519 | 1.2439 | 0.003200 | 0.008830 |
| M935 | 11.647 | 0.0097 | 0.029361 |  | M520 | 1.1113 | 0.051200 | 0.063884 |
| M943 | 7.7368 | 0.0047 | 0.02538 |  | M522 | 1.1491 | 0.022700 | 0.036291 |
| M944 | 7.3674 | 0.0058 | 0.0257566 |  | M524 | 1.299 | 0.001100 | 0.003872 |
| M949 | 13.29 | 0.0012 | 0.0215426 |  | M525 | 1.1215 | 0.042100 | 0.055669 |
| M950 | 17.102 | 0.0145 | 0.0345848 |  | M528 | 1.5102 | 0.000060 | 0.000395 |
| M953 | 7.2316 | 0.0103 | 0.0301596 |  | M529 | 1.1329 | 0.032600 | 0.046641 |
| M954 | 10.934 | 0.0035 | 0.0247277 |  | M530 | 1.156 | 0.019500 | 0.032474 |
| M955 | 12.347 | 0.004 | 0.0252336 |  | M531 | 1.129 | 0.034800 | 0.048830 |
| M959 | 4.5973 | 0.0149 | 0.0351661 |  | M533 | 1.2733 | 0.001500 | 0.004917 |
| M963 | 10.295 | 0.0043 | 0.0252391 |  | M534 | 1.5721 | 0.000020 | 0.000171 |
| M964 | 7.8298 | 0.0039 | 0.0248349 |  | M536 | 1.2027 | 0.006900 | 0.015832 |
| M974 | 3.2891 | 0.0542 | 0.074816 |  | M545 | 1.1045 | 0.059700 | 0.070902 |
| M975 | 4.9357 | 0.029 | 0.0512435 |  | M552 | 1.2374 | 0.003500 | 0.009489 |
| M982 | 21.736 | 0.0022 | 0.0225 |  | M553 | 1.1966 | 0.008200 | 0.018208 |
| M985 | 4.7034 | 0.0235 | 0.0458454 |  | M555 | 1.2161 | 0.005200 | 0.012733 |
| M986 | 3.1612 | 0.0675 | 0.0813123 |  | M557 | 1.1692 | 0.014300 | 0.026248 |
| M996 | 17.488 | 0.0009 | 0.02025 |  | M558 | 1.142 | 0.026400 | 0.040332 |
| M1007 | 4.0642 | 0.0439 | 0.0668905 |  | M560 | 1.1236 | 0.043200 | 0.056941 |
| M1010 | 3.7088 | 0.0513 | 0.0722912 |  | M563 | 1.1717 | 0.015000 | 0.027013 |
| M1016 | 2.6598 | 0.0946 | 0.0961824 |  | M564 | 1.2588 | 0.002200 | 0.006616 |
| M1021 | 6.0516 | 0.0105 | 0.0301596 |  | M565 | 1.7229 | 0.000010 | 0.000099 |
| M1023 | 3.3176 | 0.0552 | 0.0755455 |  | M567 | 1.2073 | 0.006700 | 0.015516 |
| M1024 | 12.962 | 0.0008 | 0.02 |  | M569 | 1.4142 | 0.000080 | 0.000496 |
| M1025 | 3.6796 | 0.0414 | 0.065445 |  | M570 | 1.5307 | 0.000010 | 0.000099 |
| M1028 | 6.7253 | 0.007 | 0.027905 |  | M575 | 1.956 | 0.000010 | 0.000099 |
| M1029 | 3.6217 | 0.0513 | 0.0722912 |  | M581 | 1.0879 | 0.089100 | 0.092458 |
| M1031 | 9.1189 | 0.0056 | 0.0257143 |  | M583 | 1.1047 | 0.060000 | 0.070986 |
| M1034 | 7.675 | 0.0042 | 0.0252391 |  | M585 | 1.2178 | 0.005000 | 0.012341 |
| M1035 | 2.8998 | 0.0969 | 0.0973326 |  | M587 | 1.2077 | 0.006600 | 0.015313 |
| M1037 | 3.2399 | 0.0682 | 0.0814779 |  | M589 | 1.0908 | 0.084900 | 0.089735 |
| M1041 | 6.9086 | 0.0084 | 0.0286463 |  | M590 | 1.3981 | 0.000100 | 0.000543 |
| M1044 | 3.2211 | 0.075 | 0.085371 |  | M591 | 1.1479 | 0.022900 | 0.036516 |
| M1045 | 3.2464 | 0.0663 | 0.0813123 |  | M592 | 1.4414 | 0.000090 | 0.000541 |
| M1046 | 2.7557 | 0.0866 | 0.0912988 |  | M593 | 1.2209 | 0.005300 | 0.012901 |
| M1050 | 2.3168 | 0.0675 | 0.0813123 |  | M594 | 1.1249 | 0.037100 | 0.050792 |
| M1053 | 2.3305 | 0.0653 | 0.0813123 |  | M596 | 1.4164 | 0.000100 | 0.000543 |
| M1061 | 3.0111 | 0.0836 | 0.0901438 |  | M601 | 1.1695 | 0.014800 | 0.026887 |
| M1062 | 11.734 | 0.003 | 0.0247277 |  | M606 | 1.4414 | 0.000070 | 0.000447 |
| M1064 | 8.2312 | 0.0128 | 0.0326038 |  | M607 | 1.2444 | 0.003100 | 0.008593 |
| M1068 | 3.1298 | 0.0756 | 0.0856731 |  | M609 | 1.6144 | 0.000010 | 0.000099 |
| M1070 | 9.2378 | 0.0022 | 0.0225 |  | M613 | 1.144 | 0.026400 | 0.040332 |
| M1073 | 2.8676 | 0.0566 | 0.0759543 |  | M614 | 1.5193 | 0.000020 | 0.000171 |
| M1074 | 9.736 | 0.0052 | 0.0257143 |  | M616 | 1.2252 | 0.004500 | 0.011332 |
| M1077 | 6.3931 | 0.0096 | 0.0293213 |  | M625 | 1.1305 | 0.032700 | 0.046730 |
| M1078 | 9.3794 | 0.0043 | 0.0252391 |  | M626 | 1.1756 | 0.013400 | 0.025386 |
| M1083 | 4.7081 | 0.0315 | 0.0548003 |  | M627 | 1.1402 | 0.028000 | 0.042256 |
| M1084 | 5.7004 | 0.0174 | 0.0378871 |  | M628 | 1.1454 | 0.024900 | 0.038855 |
| M1092 | 3.3177 | 0.0679 | 0.0814076 |  | M630 | 1.461 | 0.000050 | 0.000344 |
| M1097 | 7.8089 | 0.0117 | 0.0312353 |  | M631 | 1.0987 | 0.069300 | 0.078629 |
| M1106 | 5.2525 | 0.0201 | 0.041875 |  | M633 | 1.2339 | 0.004100 | 0.010561 |
| M1108 | 19.579 | 0.0015 | 0.0215426 |  | M634 | 1.353 | 0.000400 | 0.001764 |
| M1109 | 11.724 | 0.0085 | 0.0286463 |  | M635 | 1.7083 | 0.000010 | 0.000099 |
| M1110 | 9.7351 | 0.0079 | 0.028516 |  | M636 | 2.2618 | 0.000010 | 0.000099 |
| M1113 | 4.3386 | 0.0405 | 0.0646277 |  | M637 | 1.3974 | 0.000100 | 0.000543 |
| M1114 | 6.2579 | 0.023 | 0.045 |  | M640 | 1.1237 | 0.039700 | 0.053466 |
| M1115 | 5.3988 | 0.02 | 0.041875 |  | M641 | 1.1101 | 0.056700 | 0.068605 |
| M1120 | 17.674 | 0.0038 | 0.0248349 |  | M643 | 1.2319 | 0.004000 | 0.010434 |
| M1121 | 4.6287 | 0.0246 | 0.0465126 |  | M646 | 1.1649 | 0.016900 | 0.029285 |
| M1122 | 5.9763 | 0.0067 | 0.027244 |  | M648 | 1.4329 | 0.000100 | 0.000543 |
| M1123 | 3.3696 | 0.0608 | 0.0787285 |  | M654 | 1.0869 | 0.092600 | 0.094819 |
| M1125 | 6.6407 | 0.0164 | 0.0366556 |  | M658 | 1.0958 | 0.075900 | 0.083964 |
| M1132 | 6.1779 | 0.0071 | 0.027905 |  | M660 | 1.1415 | 0.025800 | 0.039858 |
| M1138 | 4.7453 | 0.0417 | 0.0654593 |  | M662 | 1.1157 | 0.047300 | 0.060793 |
| M1141 | 7.0603 | 0.0114 | 0.0311538 |  | M663 | 1.4869 | 0.000030 | 0.000235 |
| M1147 | 2.8293 | 0.0833 | 0.0901082 |  | M664 | 1.1716 | 0.014600 | 0.026681 |
| M1155 | 7.6065 | 0.0081 | 0.0286463 |  | M665 | 1.1355 | 0.030200 | 0.044598 |
| M1158 | 4.1262 | 0.0349 | 0.0587469 |  | M668 | 1.1256 | 0.038100 | 0.051875 |
| M1161 | 4.476 | 0.0359 | 0.0599814 |  | M670 | 1.2474 | 0.002700 | 0.007726 |
| M1166 | 3.2488 | 0.0674 | 0.0813123 |  | M671 | 1.578 | 0.000010 | 0.000099 |
| M1167 | 4.8604 | 0.026 | 0.0480822 |  | M672 | 1.0924 | 0.081000 | 0.087042 |
| M1168 | 6.2219 | 0.0093 | 0.0290625 |  | M675 | 2.0206 | 0.000010 | 0.000099 |
| M1172 | 11.978 | 0.0114 | 0.0311538 |  | M676 | 1.1691 | 0.015900 | 0.028103 |
| M1173 | 18.186 | 0.0065 | 0.0269172 |  | M678 | 1.5015 | 0.000040 | 0.000295 |
| M1175 | 2.2195 | 0.0745 | 0.0852331 |  | M680 | 1.1503 | 0.022100 | 0.035654 |
| M1179 | 4.2943 | 0.035 | 0.0587687 |  | M681 | 1.1954 | 0.008200 | 0.018208 |
| M1190 | 10.784 | 0.0034 | 0.0247277 |  | M683 | 1.0954 | 0.074100 | 0.082415 |
| M1194 | 7.5964 | 0.0091 | 0.028838 |  | M685 | 1.4532 | 0.000100 | 0.000543 |
| M1198 | 3.4424 | 0.0541 | 0.074816 |  | M690 | 2.2263 | 0.000010 | 0.000099 |
| M1199 | 3.2571 | 0.0719 | 0.0839663 |  | M693 | 2.3184 | 0.000010 | 0.000099 |
| M1201 | 5.6038 | 0.02 | 0.041875 |  | M694 | 1.1503 | 0.023000 | 0.036629 |
| M1202 | 5.3301 | 0.0268 | 0.0486503 |  | M698 | 1.1209 | 0.041500 | 0.055170 |
| M1205 | 23.349 | 0.0015 | 0.0215426 |  | M699 | 1.7638 | 0.000010 | 0.000099 |
| M1211 | 22.4 | 0.0077 | 0.0284595 |  | M700 | 1.1033 | 0.065700 | 0.075794 |
| M1223 | 3.7466 | 0.0505 | 0.0716124 |  | M703 | 1.1335 | 0.032300 | 0.046266 |
| M1225 | 13.186 | 0.0037 | 0.0247277 |  | M704 | 1.0899 | 0.083400 | 0.088546 |
| M1226 | 2.7928 | 0.0759 | 0.0856731 |  | M706 | 1.1634 | 0.018400 | 0.031144 |
| M1233 | 10.968 | 0.0096 | 0.0293213 |  | M707 | 1.2376 | 0.003500 | 0.009489 |
| M1235 | 4.9061 | 0.0209 | 0.0431422 |  | M710 | 1.237 | 0.003700 | 0.009859 |
| M1236 | 3.0754 | 0.0616 | 0.0793511 |  | M712 | 2.2057 | 0.000010 | 0.000099 |
| M1247 | 2.3305 | 0.0662 | 0.0813123 |  | M715 | 1.1038 | 0.063500 | 0.073805 |
| M1248 | 5.8991 | 0.0117 | 0.0312353 |  | M716 | 1.257 | 0.002400 | 0.007097 |
| M1249 | 2.3471 | 0.0757 | 0.0856731 |  | M717 | 1.2561 | 0.002200 | 0.006616 |
| M1253 | 5.1728 | 0.0191 | 0.0406703 |  | M719 | 1.4303 | 0.000060 | 0.000395 |
| M1257 | 2.869 | 0.0787 | 0.0873399 |  | M720 | 1.0948 | 0.076800 | 0.084432 |
| M1261 | 3.235 | 0.05 | 0.0716124 |  | M722 | 1.3424 | 0.000500 | 0.002107 |
| M1268 | 2.4623 | 0.0492 | 0.0711058 |  | M724 | 1.3362 | 0.000600 | 0.002383 |
| M1272 | 9.5028 | 0.0094 | 0.0291055 |  | M726 | 1.1075 | 0.055300 | 0.067504 |
| M1274 | 3.6603 | 0.059 | 0.0774612 |  | M728 | 1.7279 | 0.000010 | 0.000099 |
| M1276 | 4.7631 | 0.0218 | 0.0439254 |  | M730 | 1.1105 | 0.052800 | 0.065282 |
| M1277 | 2.8612 | 0.0835 | 0.0901438 |  | M731 | 1.1833 | 0.010100 | 0.021282 |
| M1287 | 3.6392 | 0.0488 | 0.0709914 |  | M733 | 1.128 | 0.034900 | 0.048860 |
| M1291 | 4.1363 | 0.0381 | 0.06227 |  | M734 | 1.2713 | 0.001700 | 0.005443 |
| M1294 | 2.9002 | 0.0859 | 0.0912988 |  | M735 | 1.181 | 0.011300 | 0.023065 |
| M1296 | 6.7893 | 0.009 | 0.0286557 |  | M737 | 1.2638 | 0.002100 | 0.006377 |
| M1297 | 2.9446 | 0.0825 | 0.0896739 |  | M740 | 1.2513 | 0.003000 | 0.008372 |
| M1302 | 4.6238 | 0.0223 | 0.0444027 |  | M741 | 1.2533 | 0.002700 | 0.007726 |
| M1305 | 9.3206 | 0.0072 | 0.027905 |  | M743 | 1.2423 | 0.003600 | 0.009634 |
| M1307 | 3.7271 | 0.0509 | 0.0720283 |  | M746 | 1.5128 | 0.000020 | 0.000171 |
| M1319 | 7.6084 | 0.0022 | 0.0225 |  | M747 | 1.7378 | 0.000020 | 0.000171 |
| M1322 | 19.042 | 0.0011 | 0.0215426 |  | M748 | 1.6906 | 0.000010 | 0.000099 |
| M1323 | 3.2327 | 0.0744 | 0.0852331 |  | M749 | 1.134 | 0.031400 | 0.045502 |
| M1329 | 3.4824 | 0.0599 | 0.0783576 |  | M751 | 1.0854 | 0.093400 | 0.095323 |
| M1341 | 4.019 | 0.0459 | 0.0682434 |  | M753 | 1.1016 | 0.062600 | 0.073102 |
| M1343 | 2.9768 | 0.0846 | 0.090787 |  | M754 | 1.0841 | 0.094500 | 0.096367 |
| M1344 | 6.6542 | 0.0086 | 0.0286463 |  | M755 | 1.5933 | 0.000010 | 0.000099 |
| M1347 | 4.6462 | 0.0258 | 0.0480822 |  | M756 | 1.097 | 0.072500 | 0.081439 |
| M1349 | 8.4529 | 0.0021 | 0.0225 |  | M758 | 1.1055 | 0.060000 | 0.070986 |
| M1355 | 9.1214 | 0.0101 | 0.030033 |  | M759 | 1.3787 | 0.000100 | 0.000543 |
| M1361 | 6.7286 | 0.0172 | 0.0378871 |  | M762 | 1.0979 | 0.068700 | 0.078163 |
| M1362 | 2.8292 | 0.0804 | 0.0881006 |  | M764 | 1.0813 | 0.099400 | 0.099722 |
| M1373 | 4.0108 | 0.0571 | 0.0764554 |  | M765 | 1.2252 | 0.004800 | 0.012015 |
| M1375 | 4.3537 | 0.0419 | 0.0654688 |  | M766 | 1.1046 | 0.058400 | 0.069978 |
| M1386 | 3.4965 | 0.0609 | 0.0787285 |  | M767 | 1.2274 | 0.004400 | 0.011194 |
| M1387 | 3.2908 | 0.0725 | 0.084201 |  | M768 | 1.1058 | 0.057700 | 0.069408 |
| M1388 | 3.5083 | 0.0565 | 0.0759543 |  | M769 | 1.1106 | 0.052000 | 0.064622 |
| M1393 | 9.4289 | 0.0021 | 0.0225 |  | M770 | 1.1398 | 0.028300 | 0.042605 |
| M1397 | 4.4281 | 0.0505 | 0.0716124 |  | M771 | 1.503 | 0.000020 | 0.000171 |
| M1398 | 12.034 | 0.0044 | 0.02538 |  | M773 | 1.2923 | 0.001100 | 0.003872 |
| M1399 | 7.8375 | 0.0164 | 0.0366556 |  | M779 | 1.253 | 0.002300 | 0.006850 |
| M1400 | 6.3716 | 0.0138 | 0.0335072 |  | M781 | 1.3627 | 0.000300 | 0.001392 |
| M1404 | 2.7345 | 0.098 | 0.0982912 |  | M787 | 1.0873 | 0.086600 | 0.090623 |
| M1411 | 3.8249 | 0.0453 | 0.06795 |  | M794 | 1.1876 | 0.024600 | 0.038679 |
| M1419 | 3.4776 | 0.0583 | 0.0773134 |  | M799 | 1.0948 | 0.074200 | 0.082452 |
| M1420 | 6.3463 | 0.0123 | 0.0318103 |  | M801 | 1.1436 | 0.025900 | 0.039913 |
| M1421 | 13.909 | 0.0034 | 0.0247277 |  | M804 | 1.4952 | 0.000040 | 0.000295 |
| M1431 | 3.0708 | 0.0744 | 0.0852331 |  | M807 | 1.1628 | 0.016200 | 0.028511 |
| M1436 | 3.0324 | 0.0803 | 0.0881006 |  | M808 | 1.5442 | 0.000010 | 0.000099 |
| M1437 | 3.7129 | 0.0493 | 0.0711058 |  | M810 | 1.1594 | 0.018700 | 0.031395 |
| M1439 | 10.093 | 0.0028 | 0.0247277 |  | M811 | 1.2351 | 0.004100 | 0.010561 |
| M1442 | 10.541 | 0.0034 | 0.0247277 |  | M816 | 1.4323 | 0.000080 | 0.000496 |
| M1443 | 10.456 | 0.0085 | 0.0286463 |  | M818 | 1.3928 | 0.000200 | 0.000999 |
| M1444 | 2.7989 | 0.093 | 0.095258 |  | M820 | 1.1522 | 0.021100 | 0.034626 |
| M1448 | 5.1205 | 0.0161 | 0.0363462 |  | M821 | 1.1351 | 0.030600 | 0.044921 |
| M1455 | 10.803 | 0.0039 | 0.0248349 |  | M825 | 1.0848 | 0.090600 | 0.093467 |
| M1463 | 18.319 | 0.0046 | 0.02538 |  | M826 | 1.0977 | 0.069600 | 0.078897 |
| M1467 | 8.7188 | 0.0036 | 0.0247277 |  | M827 | 1.3186 | 0.000600 | 0.002383 |
| M1468 | 2.8434 | 0.0728 | 0.0842882 |  | M828 | 1.1854 | 0.011300 | 0.023065 |
| M1469 | 2.707 | 0.0935 | 0.0954803 |  | M829 | 1.1292 | 0.035300 | 0.049253 |
| M1473 | 7.5746 | 0.0052 | 0.0257143 |  | M832 | 1.1634 | 0.017400 | 0.029860 |
| M1490 | 2.661 | 0.0661 | 0.0813123 |  | M838 | 2.0983 | 0.000010 | 0.000099 |
| M1492 | 4.0802 | 0.0411 | 0.0651232 |  | M839 | 1.0904 | 0.080700 | 0.086945 |
| M1503 | 10.917 | 0.0009 | 0.02025 |  | M840 | 1.2053 | 0.006900 | 0.015832 |
| M1509 | 7.2107 | 0.0071 | 0.027905 |  | M842 | 1.0874 | 0.098000 | 0.098557 |
| M1511 | 10.961 | 0.0003 | 0.016875 |  | M843 | 1.206 | 0.007300 | 0.016505 |
| M1512 | 5.3848 | 0.0243 | 0.0463347 |  | M844 | 1.1678 | 0.015000 | 0.027013 |
| M1513 | 8.3287 | 0.0021 | 0.0225 |  | M846 | 1.1289 | 0.036200 | 0.049946 |
| M1516 | 6.3075 | 0.0087 | 0.0286463 |  | M849 | 1.235 | 0.004300 | 0.010962 |
| M1520 | 6.512 | 0.0064 | 0.0269172 |  | M852 | 1.3288 | 0.000600 | 0.002383 |
| M1525 | 3.6121 | 0.0477 | 0.0699946 |  | M854 | 1.3156 | 0.000800 | 0.003059 |
| M1529 | 19.114 | 0.0013 | 0.0215426 |  | M855 | 1.1754 | 0.013400 | 0.025386 |
| M1538 | 16.839 | 0.000020 | 0.0135 |  | M857 | 1.3201 | 0.000700 | 0.002736 |
| M1545 | 13.859 | 0.0043 | 0.0252391 |  | M860 | 1.0886 | 0.086900 | 0.090860 |
| M1546 | 7.7263 | 0.0025 | 0.0241071 |  | M863 | 1.1476 | 0.023100 | 0.036741 |
| M1553 | 5.0004 | 0.0179 | 0.0388505 |  | M865 | 1.0906 | 0.080300 | 0.086773 |
| M1556 | 4.1295 | 0.0416 | 0.0654593 |  | M866 | 1.1829 | 0.011300 | 0.023065 |
| M1560 | 18.186 | 0.003 | 0.0247277 |  | M867 | 1.1597 | 0.018300 | 0.031017 |
| M1561 | 4.7177 | 0.028 | 0.0498681 |  | M870 | 1.1166 | 0.048400 | 0.061568 |
| M1562 | 5.609 | 0.0156 | 0.0355743 |  | M875 | 1.2238 | 0.004500 | 0.011332 |
| M1563 | 6.3187 | 0.0078 | 0.0284595 |  | M876 | 1.0888 | 0.084800 | 0.089724 |
| M1564 | 4.8994 | 0.0247 | 0.0465712 |  | M877 | 1.1012 | 0.063800 | 0.074015 |
| M1565 | 3.8707 | 0.0927 | 0.0951976 |  | M878 | 1.3678 | 0.000300 | 0.001392 |
| M1569 | 3.5815 | 0.0519 | 0.0729844 |  | M879 | 1.5793 | 0.000020 | 0.000171 |
| M1572 | 3.2922 | 0.065 | 0.0813123 |  | M881 | 1.1945 | 0.009100 | 0.019711 |
| M1573 | 3.9747 | 0.0419 | 0.0654688 |  | M883 | 1.1673 | 0.015300 | 0.027434 |
| M1575 | 7.599 | 0.0059 | 0.0258604 |  | M885 | 1.2288 | 0.003900 | 0.010238 |
| M1576 | 7.9549 | 0.0045 | 0.02538 |  | M889 | 1.0904 | 0.085800 | 0.090272 |
| M1577 | 6.5866 | 0.0114 | 0.0311538 |  | M890 | 1.0916 | 0.080400 | 0.086773 |
| M1579 | 12.187 | 0.0056 | 0.0257143 |  | M891 | 1.1511 | 0.022600 | 0.036224 |
| M1582 | 6.8076 | 0.0087 | 0.0286463 |  | M893 | 1.3352 | 0.000500 | 0.002107 |
| M1583 | 6.3911 | 0.0118 | 0.0312353 |  | M897 | 1.3902 | 0.000300 | 0.001392 |
| M1586 | 7.9382 | 0.0121 | 0.031657 |  | M899 | 1.5613 | 0.000010 | 0.000099 |
| M1587 | 4.0865 | 0.0399 | 0.064125 |  | M900 | 1.1084 | 0.056100 | 0.068200 |
| M1590 | 5.9139 | 0.0134 | 0.0328909 |  | M904 | 1.1247 | 0.037900 | 0.051773 |
| M1591 | 3.149 | 0.0677 | 0.0813123 |  | M908 | 1.1525 | 0.023200 | 0.036805 |
| M1592 | 4.8788 | 0.0254 | 0.0477577 |  | M909 | 1.1378 | 0.029900 | 0.044473 |
| M1595 | 21.039 | 0.0026 | 0.024375 |  | M910 | 1.1922 | 0.009200 | 0.019824 |
| M1598 | 6.6627 | 0.0105 | 0.0301596 |  | M912 | 1.2787 | 0.001500 | 0.004917 |
| M1599 | 18.342 | 0.0133 | 0.0328846 |  | M914 | 1.1451 | 0.025500 | 0.039642 |
| M1601 | 3.2142 | 0.0734 | 0.084663 |  | M915 | 1.1919 | 0.012000 | 0.023987 |
| M1605 | 11.384 | 0.0002 | 0.016875 |  | M917 | 1.2131 | 0.006300 | 0.014784 |
| M1607 | 4.6601 | 0.0316 | 0.0548329 |  | M919 | 1.1804 | 0.012100 | 0.023987 |
| M1609 | 6.7845 | 0.0055 | 0.0257143 |  | M921 | 1.0946 | 0.073100 | 0.081855 |
| M1610 | 16.681 | 0.0058 | 0.0257566 |  | M922 | 1.1798 | 0.012300 | 0.024037 |
| M1612 | 2.6884 | 0.0817 | 0.0890913 |  | M923 | 1.3511 | 0.000500 | 0.002107 |
| M1614 | 2.9873 | 0.0645 | 0.0810754 |  | M926 | 1.1278 | 0.038400 | 0.052226 |
| M1619 | 6.6553 | 0.0042 | 0.0252391 |  | M928 | 1.2905 | 0.001300 | 0.004377 |
| M1630 | 9.0367 | 0.0104 | 0.0301596 |  | M930 | 1.729 | 0.000010 | 0.000099 |
| M1631 | 2.9595 | 0.0699 | 0.0824869 |  | M931 | 1.5861 | 0.000010 | 0.000099 |
| M1635 | 3.8797 | 0.0451 | 0.06795 |  | M932 | 1.1119 | 0.053600 | 0.066014 |
| M1638 | 4.7062 | 0.0228 | 0.0448688 |  | M934 | 1.2467 | 0.002900 | 0.008148 |
| M1643 | 9.5599 | 0.0013 | 0.0215426 |  | M935 | 1.4941 | 0.000020 | 0.000171 |
| M1647 | 4.5414 | 0.032 | 0.055102 |  | M937 | 1.1115 | 0.052400 | 0.064983 |
| M1649 | 8.959 | 0.006 | 0.0259615 |  | M938 | 1.1631 | 0.016400 | 0.028619 |
| M1652 | 17.579 | 0.0026 | 0.024375 |  | M940 | 1.1058 | 0.058600 | 0.070082 |
| M1657 | 14.823 | 0.0048 | 0.0255118 |  | M941 | 1.2006 | 0.007500 | 0.016895 |
| M1658 | 13.368 | 0.0065 | 0.0269172 |  | M942 | 1.169 | 0.014500 | 0.026537 |
| M1663 | 9.6604 | 0.0036 | 0.0247277 |  | M943 | 1.8171 | 0.000010 | 0.000099 |
| M1665 | 6.462 | 0.0075 | 0.028125 |  | M944 | 1.7822 | 0.000010 | 0.000099 |
| M1670 | 3.2263 | 0.069 | 0.0818541 |  | M947 | 1.123 | 0.039500 | 0.053312 |
| M1696 | 2.9395 | 0.0602 | 0.0784459 |  | M949 | 1.252 | 0.002900 | 0.008148 |
| M1699 | 6.1824 | 0.0067 | 0.027244 |  | M950 | 2.1375 | 0.000010 | 0.000099 |
| M1705 | 2.9595 | 0.0843 | 0.0906091 |  | M953 | 1.4191 | 0.000070 | 0.000447 |
| M1712 | 2.9524 | 0.077 | 0.0863866 |  | M954 | 1.4429 | 0.000060 | 0.000395 |
| M1715 | 9.81 | 0.0105 | 0.0301596 |  | M955 | 1.566 | 0.000010 | 0.000099 |
| M1723 | 6.1524 | 0.0158 | 0.0359091 |  | M956 | 1.1177 | 0.045200 | 0.058826 |
| M1725 | 11.101 | 0.009 | 0.0286557 |  | M958 | 1.1192 | 0.046400 | 0.059947 |
| M1728 | 6.0899 | 0.0105 | 0.0301596 |  | M963 | 1.354 | 0.000300 | 0.001392 |
| M1740 | 3.0075 | 0.0832 | 0.0901082 |  | M964 | 1.1152 | 0.048100 | 0.061313 |
| M1741 | 19.52 | 0.0083 | 0.0286463 |  | M971 | 1.2343 | 0.004100 | 0.010561 |
| M1742 | 5.5439 | 0.0198 | 0.041875 |  | M974 | 1.194 | 0.008000 | 0.017892 |
| M1744 | 2.9001 | 0.091 | 0.0937786 |  | M975 | 1.1045 | 0.060300 | 0.071086 |
| M1745 | 13.333 | 0.0055 | 0.0257143 |  | M976 | 1.1225 | 0.043100 | 0.056870 |
| M1747 | 4.9383 | 0.0267 | 0.0486503 |  | M977 | 1.1357 | 0.030000 | 0.044515 |
| M1748 | 4.4686 | 0.0332 | 0.0565909 |  | M978 | 1.1516 | 0.021900 | 0.035423 |
| M1757 | 22.799 | 0.0016 | 0.0216 |  | M980 | 1.4907 | 0.000060 | 0.000395 |
| M1765 | 4.679 | 0.0288 | 0.0511579 |  | M981 | 1.2437 | 0.003300 | 0.009026 |
| M1767 | 4.2551 | 0.0345 | 0.0583647 |  | M982 | 2.2154 | 0.000010 | 0.000099 |
| M1768 | 11.344 | 0.0018 | 0.0225 |  | M984 | 1.1626 | 0.016400 | 0.028619 |
| M1770 | 2.8265 | 0.0773 | 0.0863866 |  | M985 | 1.1173 | 0.045400 | 0.059025 |
| M1773 | 3.2885 | 0.0652 | 0.0813123 |  | M986 | 1.1614 | 0.016900 | 0.029285 |
| M1775 | 4.1087 | 0.0565 | 0.0759543 |  | M987 | 1.1226 | 0.041500 | 0.055170 |
| M1776 | 7.057 | 0.0126 | 0.0324618 |  | M989 | 1.2144 | 0.006000 | 0.014241 |
| M1779 | 2.8706 | 0.0668 | 0.0813123 |  | M990 | 1.091 | 0.082200 | 0.087722 |
| M1789 | 3.8963 | 0.0432 | 0.0668807 |  | M996 | 1.6684 | 0.000010 | 0.000099 |
| M1800 | 14.897 | 0.0049 | 0.0257143 |  | M997 | 1.1548 | 0.019600 | 0.032597 |
| M1802 | 5.5147 | 0.0174 | 0.0378871 |  | M998 | 1.1196 | 0.042300 | 0.055874 |
| M1806 | 3.4602 | 0.0668 | 0.0813123 |  | M999 | 1.157 | 0.019300 | 0.032227 |
| M1812 | 6.4683 | 0.0127 | 0.0324716 |  | M1001 | 1.1711 | 0.014000 | 0.026084 |
| M1817 | 19.174 | 0.0078 | 0.0284595 |  | M1002 | 1.3102 | 0.000800 | 0.003059 |
| M1824 | 7.8232 | 0.0088 | 0.0286557 |  | M1006 | 1.0903 | 0.085300 | 0.089870 |
| M1825 | 3.38 | 0.0636 | 0.080243 |  | M1007 | 1.1553 | 0.020100 | 0.033250 |
| M1826 | 3.4523 | 0.0523 | 0.0732417 |  | M1008 | 1.0975 | 0.073300 | 0.081892 |
| M1830 | 3.7044 | 0.0722 | 0.0841422 |  | M1009 | 1.1601 | 0.017100 | 0.029467 |
| M1831 | 13.141 | 0.0074 | 0.027905 |  | M1010 | 1.1361 | 0.030600 | 0.044921 |
| M1832 | 12.38 | 0.0057 | 0.0257566 |  | M1012 | 1.081 | 0.097300 | 0.098492 |
| M1835 | 16.886 | 0.0089 | 0.0286557 |  | M1014 | 1.1427 | 0.027900 | 0.042156 |
| M1836 | 3.1304 | 0.0665 | 0.0813123 |  | M1015 | 1.1044 | 0.062500 | 0.073054 |
| M1840 | 13.38 | 0.001 | 0.0210938 |  | M1017 | 1.3316 | 0.000600 | 0.002383 |
| M1848 | 5.4854 | 0.0144 | 0.0344681 |  | M1018 | 1.0925 | 0.080900 | 0.087010 |
| M1852 | 8.6304 | 0.0046 | 0.02538 |  | M1021 | 1.3336 | 0.000500 | 0.002107 |
| M1854 | 6.2526 | 0.0199 | 0.041875 |  | M1022 | 1.1176 | 0.046500 | 0.060014 |
| M1855 | 6.2068 | 0.0146 | 0.0347007 |  | M1023 | 1.137 | 0.030600 | 0.044921 |
| M1858 | 4.0036 | 0.0433 | 0.0668822 |  | M1024 | 1.4885 | 0.000020 | 0.000171 |
| M1860 | 5.7197 | 0.0087 | 0.0286463 |  | M1027 | 1.0876 | 0.090900 | 0.093620 |
| M1861 | 3.4872 | 0.053 | 0.0739153 |  | M1028 | 1.2852 | 0.001200 | 0.004142 |
| M1869 | 8.1099 | 0.0036 | 0.0247277 |  | M1029 | 1.1567 | 0.019800 | 0.032841 |
| M1878 | 11.185 | 0.0033 | 0.0247277 |  | M1030 | 1.0877 | 0.088900 | 0.092458 |
| M1884 | 3.1174 | 0.0529 | 0.0739153 |  | M1031 | 1.45 | 0.000090 | 0.000541 |
| M1885 | 4.6253 | 0.026 | 0.0480822 |  | M1034 | 1.316 | 0.000600 | 0.002383 |
| M1887 | 3.9549 | 0.048 | 0.070282 |  | M1037 | 1.2139 | 0.005300 | 0.012901 |
| M1892 | 11.132 | 0.0006 | 0.02 |  | M1040 | 1.1431 | 0.025700 | 0.039803 |
| M1893 | 5.857 | 0.0161 | 0.0363462 |  | M1041 | 1.2925 | 0.001000 | 0.003602 |
| M1894 | 4.1975 | 0.0389 | 0.062817 |  | M1042 | 1.1657 | 0.015800 | 0.028086 |
| M1895 | 4.8411 | 0.0237 | 0.0459698 |  | M1045 | 1.1442 | 0.025800 | 0.039858 |
| M1896 | 6.589 | 0.012 | 0.0315175 |  | M1047 | 1.1535 | 0.021300 | 0.034816 |
| M1907 | 11.38 | 0.0061 | 0.0262261 |  | M1048 | 1.1919 | 0.012000 | 0.023987 |
| M1908 | 8.7287 | 0.0029 | 0.0247277 |  | M1049 | 1.3431 | 0.000600 | 0.002383 |
| M1911 | 5.9567 | 0.0166 | 0.0368586 |  | M1050 | 1.3766 | 0.000300 | 0.001392 |
| M1913 | 4.4785 | 0.0313 | 0.0547345 |  | M1051 | 1.2878 | 0.001300 | 0.004377 |
| M1919 | 4.763 | 0.0308 | 0.054 |  | M1052 | 1.0982 | 0.072200 | 0.081176 |
| M1920 | 3.3258 | 0.0554 | 0.0755455 |  | M1056 | 1.1126 | 0.049800 | 0.062642 |
| M1924 | 7.7788 | 0.0073 | 0.027905 |  | M1060 | 1.1365 | 0.030800 | 0.045001 |
| M1925 | 6.4404 | 0.0072 | 0.027905 |  | M1062 | 1.6643 | 0.000010 | 0.000099 |
| M1926 | 10.63 | 0.008 | 0.0285714 |  | M1063 | 1.137 | 0.034800 | 0.048830 |
| M1934 | 2.5257 | 0.0885 | 0.0921396 |  | M1064 | 1.1266 | 0.035800 | 0.049727 |
| M1935 | 7.3971 | 0.0107 | 0.0302197 |  | M1066 | 1.1472 | 0.024300 | 0.038403 |
| M1936 | 14.617 | 0.0008 | 0.02 |  | M1069 | 1.1587 | 0.018600 | 0.031269 |
| M1937 | 2.2719 | 0.0913 | 0.0939444 |  | M1070 | 1.1838 | 0.011000 | 0.022602 |
| M1939 | 22.4 | 0.008 | 0.0285714 |  | M1072 | 1.1446 | 0.026000 | 0.040017 |
| M1941 | 2.7235 | 0.0867 | 0.0912988 |  | M1073 | 1.1056 | 0.059400 | 0.070698 |
| M1943 | 9.2622 | 0.007 | 0.027905 |  | M1074 | 1.4661 | 0.000070 | 0.000447 |
| M1949 | 5.5514 | 0.0118 | 0.0312353 |  | M1075 | 1.0927 | 0.076400 | 0.084217 |
| M1953 | 8.3597 | 0.0056 | 0.0257143 |  | M1076 | 1.0827 | 0.097200 | 0.098472 |
| M1960 | 15.202 | 0.0129 | 0.0326124 |  | M1077 | 1.4481 | 0.000050 | 0.000344 |
| M1981 | 3.3706 | 0.0501 | 0.0716124 |  | M1078 | 1.2089 | 0.005800 | 0.013954 |
| M1982 | 2.204 | 0.0782 | 0.087104 |  | M1083 | 1.1114 | 0.051400 | 0.064005 |
| M1987 | 2.3305 | 0.0681 | 0.0814779 |  | M1084 | 1.3245 | 0.000600 | 0.002383 |
| M1991 | 2.1324 | 0.0945 | 0.0961824 |  | M1088 | 1.0977 | 0.066200 | 0.076228 |
| M1996 | 3.3903 | 0.069 | 0.0818541 |  | M1091 | 1.1605 | 0.016900 | 0.029285 |
| M1997 | 12.678 | 0.0005 | 0.02 |  | M1092 | 1.1916 | 0.009700 | 0.020721 |
| M2009 | 4.0149 | 0.0444 | 0.0673483 |  | M1094 | 1.0864 | 0.089100 | 0.092458 |
| M2012 | 12.799 | 0.0054 | 0.0257143 |  | M1096 | 1.1066 | 0.056200 | 0.068200 |
| M2015 | 3.2904 | 0.0634 | 0.080243 |  | M1097 | 1.3704 | 0.000200 | 0.000999 |
| M2027 | 3.1193 | 0.0677 | 0.0813123 |  | M1100 | 1.0823 | 0.097100 | 0.098472 |
| M2028 | 5.37 | 0.0274 | 0.0489286 |  | M1105 | 1.1309 | 0.034000 | 0.048089 |
| M2035 | 5.2064 | 0.0214 | 0.0437123 |  | M1106 | 1.238 | 0.003800 | 0.010103 |
| M2038 | 2.6304 | 0.0958 | 0.0967164 |  | M1107 | 1.3882 | 0.000200 | 0.000999 |
| M2039 | 2.6986 | 0.0887 | 0.0921396 |  | M1108 | 1.7031 | 0.000010 | 0.000099 |
| M2040 | 12.738 | 0.0037 | 0.0247277 |  | M1110 | 1.896 | 0.000010 | 0.000099 |
| M2042 | 24.433 | 0.0008 | 0.02 |  | M1112 | 1.1277 | 0.036700 | 0.050412 |
| M2043 | 10.732 | 0.0004 | 0.02 |  | M1113 | 1.2471 | 0.002900 | 0.008148 |
| M2053 | 2.9185 | 0.0858 | 0.0912988 |  | M1114 | 1.1691 | 0.014200 | 0.026248 |
| M2061 | 4.9253 | 0.0565 | 0.0759543 |  | M1115 | 1.2027 | 0.007200 | 0.016368 |
| M2085 | 2.9318 | 0.0881 | 0.0919127 |  | M1118 | 1.2293 | 0.003900 | 0.010238 |
| M2093 | 4.6443 | 0.0445 | 0.0673487 |  | M1120 | 1.5619 | 0.000030 | 0.000235 |
| M2096 | 3.5185 | 0.0538 | 0.0747222 |  | M1121 | 1.2587 | 0.002000 | 0.006164 |
| M2097 | 3.0978 | 0.075 | 0.085371 |  | M1122 | 1.3529 | 0.000400 | 0.001764 |
| M2100 | 18.655 | 0.0088 | 0.0286557 |  | M1123 | 1.1389 | 0.028900 | 0.043403 |
| M2101 | 10.983 | 0.0118 | 0.0312353 |  | M1124 | 1.1515 | 0.021400 | 0.034888 |
| M2103 | 2.7021 | 0.0749 | 0.085371 |  | M1125 | 1.1885 | 0.009500 | 0.020399 |
| M2107 | 3.6065 | 0.058 | 0.0770669 |  | M1127 | 1.1009 | 0.067000 | 0.076793 |
| M2113 | 14.251 | 0.0042 | 0.0252391 |  | M1130 | 1.1106 | 0.054600 | 0.066980 |
| M2114 | 5.6277 | 0.0134 | 0.0328909 |  | M1132 | 1.4404 | 0.000070 | 0.000447 |
| M2116 | 2.8907 | 0.0872 | 0.0913605 |  | M1133 | 1.3134 | 0.001000 | 0.003602 |
| M2117 | 10.566 | 0.0022 | 0.0225 |  | M1139 | 1.122 | 0.040400 | 0.054173 |
| M2131 | 7.0667 | 0.0154 | 0.0355743 |  | M1140 | 1.2999 | 0.000900 | 0.003349 |
| M2133 | 14.195 | 0.0022 | 0.0225 |  | M1141 | 1.4032 | 0.000200 | 0.000999 |
| M2134 | 3.2997 | 0.059 | 0.0774612 |  | M1147 | 1.0822 | 0.097900 | 0.098536 |
| M2144 | 24.014 | 0.0008 | 0.02 |  | M1148 | 1.1284 | 0.036500 | 0.050304 |
| M2151 | 13.132 | 0.0055 | 0.0257143 |  | M1151 | 1.2985 | 0.001000 | 0.003602 |
| M2152 | 16.596 | 0.0012 | 0.0215426 |  | M1153 | 1.1222 | 0.041400 | 0.055170 |
| M2154 | 11.608 | 0.0087 | 0.0286463 |  | M1155 | 1.5186 | 0.000050 | 0.000344 |
| M2155 | 3.4608 | 0.0628 | 0.0802358 |  | M1157 | 1.0889 | 0.081900 | 0.087629 |
| M2158 | 2.3234 | 0.0862 | 0.0912988 |  | M1161 | 1.1609 | 0.017400 | 0.029860 |
| M2179 | 6.6118 | 0.0056 | 0.0257143 |  | M1163 | 1.1911 | 0.009200 | 0.019824 |
| M2186 | 2.6312 | 0.096 | 0.0967164 |  | M1166 | 1.1975 | 0.008600 | 0.018859 |
| M2188 | 7.5288 | 0.0092 | 0.0288837 |  | M1167 | 1.1062 | 0.057400 | 0.069182 |
| M2191 | 9.1007 | 0.0057 | 0.0257566 |  | M1168 | 1.3512 | 0.000400 | 0.001764 |
| M2198 | 3.1594 | 0.0692 | 0.0819474 |  | M1171 | 1.1805 | 0.012400 | 0.024157 |
| M2206 | 5.4072 | 0.0188 | 0.0401582 |  | M1172 | 1.4747 | 0.000040 | 0.000295 |
| M2208 | 4.4436 | 0.0323 | 0.0554771 |  | M1173 | 1.7952 | 0.000010 | 0.000099 |
| M2215 | 6.3563 | 0.0106 | 0.0301899 |  | M1176 | 1.1258 | 0.039100 | 0.052945 |
| M2216 | 7.2113 | 0.0037 | 0.0247277 |  | M1178 | 1.1354 | 0.030000 | 0.044515 |
| M2217 | 20.705 | 0.0012 | 0.0215426 |  | M1179 | 1.1219 | 0.040500 | 0.054248 |
| M2220 | 8.713 | 0.0012 | 0.0215426 |  | M1181 | 1.1073 | 0.056700 | 0.068605 |
| M2222 | 5.1837 | 0.0227 | 0.0448688 |  | M1184 | 1.1186 | 0.044400 | 0.058152 |
| M2228 | 10.772 | 0.0053 | 0.0257143 |  | M1187 | 1.2982 | 0.001200 | 0.004142 |
| M2232 | 4.9719 | 0.0259 | 0.0480822 |  | M1189 | 1.1129 | 0.049500 | 0.062455 |
| M2235 | 4.9021 | 0.0222 | 0.0443343 |  | M1190 | 1.264 | 0.002100 | 0.006377 |
| M2239 | 3.0221 | 0.0861 | 0.0912988 |  | M1191 | 1.2925 | 0.001100 | 0.003872 |
| M2240 | 4.7651 | 0.0273 | 0.0488793 |  | M1194 | 1.1844 | 0.011400 | 0.023193 |
| M2245 | 4.013 | 0.0473 | 0.0698632 |  | M1198 | 1.2223 | 0.004900 | 0.012191 |
| M2252 | 3.1863 | 0.055 | 0.0755455 |  | M1199 | 1.1578 | 0.019300 | 0.032227 |
| M2257 | 12.275 | 0.0025 | 0.0241071 |  | M1201 | 1.1102 | 0.052100 | 0.064681 |
| M2259 | 2.7996 | 0.0635 | 0.080243 |  | M1202 | 1.2297 | 0.003900 | 0.010238 |
| M2262 | 3.6788 | 0.0541 | 0.074816 |  | M1203 | 1.1007 | 0.067500 | 0.077294 |
| M2272 | 15.823 | 0.007 | 0.027905 |  | M1204 | 1.0916 | 0.078200 | 0.085365 |
| M2274 | 3.8832 | 0.0466 | 0.0691319 |  | M1205 | 2.1859 | 0.000010 | 0.000099 |
| M2277 | 3.0212 | 0.0786 | 0.0873399 |  | M1208 | 1.1444 | 0.025600 | 0.039747 |
| M2280 | 15.545 | 0.0035 | 0.0247277 |  | M1210 | 1.2274 | 0.004100 | 0.010561 |
| M2282 | 11.171 | 0.0039 | 0.0248349 |  | M1211 | 2.4727 | 0.000010 | 0.000099 |
| M2287 | 6.4428 | 0.009 | 0.0286557 |  | M1214 | 1.2609 | 0.002300 | 0.006850 |
| M2288 | 12.792 | 0.0037 | 0.0247277 |  | M1225 | 1.5967 | 0.000010 | 0.000099 |
| M2295 | 3.1121 | 0.0723 | 0.0841422 |  | M1226 | 1.1466 | 0.024700 | 0.038689 |
| M2303 | 3.9235 | 0.0499 | 0.0716124 |  | M1228 | 1.2832 | 0.001300 | 0.004377 |
| M2311 | 3.7492 | 0.0591 | 0.0774612 |  | M1233 | 1.5739 | 0.000010 | 0.000099 |
| M2312 | 3.2743 | 0.0776 | 0.0865785 |  | M1235 | 1.1765 | 0.012600 | 0.024355 |
| M2314 | 2.9457 | 0.0591 | 0.0774612 |  | M1239 | 1.2449 | 0.003300 | 0.009026 |
| M2315 | 2.7321 | 0.096 | 0.0967164 |  | M1240 | 1.2445 | 0.003100 | 0.008593 |
| M2316 | 8.4777 | 0.0116 | 0.0312353 |  | M1243 | 1.1306 | 0.035800 | 0.049727 |
| M2317 | 5.5419 | 0.0229 | 0.0449346 |  | M1245 | 1.1919 | 0.012100 | 0.023987 |
| M2319 | 7.0103 | 0.0037 | 0.0247277 |  | M1247 | 1.1193 | 0.047400 | 0.060796 |
| M2320 | 11.239 | 0.0021 | 0.0225 |  | M1248 | 1.6472 | 0.000010 | 0.000099 |
| M2323 | 13.799 | 0.0059 | 0.0258604 |  | M1249 | 1.3074 | 0.000900 | 0.003349 |
| M2324 | 8.0304 | 0.0073 | 0.027905 |  | M1250 | 1.1112 | 0.053100 | 0.065464 |
| M2325 | 2.9134 | 0.0888 | 0.0921396 |  | M1253 | 1.1992 | 0.008100 | 0.018050 |
| M2339 | 3.9952 | 0.0402 | 0.0643009 |  | M1256 | 1.133 | 0.035200 | 0.049224 |
| M2340 | 6.7556 | 0.0051 | 0.0257143 |  | M1257 | 1.1365 | 0.029300 | 0.043791 |
| M2342 | 3.2573 | 0.0633 | 0.080243 |  | M1260 | 1.1154 | 0.049200 | 0.062266 |
| M2343 | 6.5679 | 0.0102 | 0.0301596 |  | M1261 | 1.3073 | 0.001000 | 0.003602 |
| M2347 | 13.177 | 0.0098 | 0.0294 |  | M1262 | 1.2868 | 0.001100 | 0.003872 |
| M2361 | 16.176 | 0.0107 | 0.0302197 |  | M1264 | 1.0854 | 0.091900 | 0.094493 |
| M2363 | 2.9659 | 0.0804 | 0.0881006 |  | M1270 | 1.1424 | 0.026500 | 0.040435 |
| M2364 | 7.4516 | 0.0052 | 0.0257143 |  | M1272 | 1.866 | 0.000010 | 0.000099 |
| M2367 | 3.6004 | 0.0523 | 0.0732417 |  | M1275 | 1.2464 | 0.005000 | 0.012341 |
| M2368 | 5.5251 | 0.0147 | 0.0348158 |  | M1276 | 1.1602 | 0.018000 | 0.030635 |
| M2369 | 7.0187 | 0.0144 | 0.0344681 |  | M1277 | 1.0814 | 0.097900 | 0.098536 |
| M2371 | 5.3441 | 0.0139 | 0.033629 |  | M1282 | 1.1916 | 0.009500 | 0.020399 |
| M2372 | 14.731 | 0.0039 | 0.0248349 |  | M1283 | 1.1349 | 0.031400 | 0.045502 |
| M2373 | 10.724 | 0.011 | 0.0305556 |  | M1291 | 1.2133 | 0.006000 | 0.014241 |
| M2378 | 4.7217 | 0.0262 | 0.048188 |  | M1294 | 1.1676 | 0.015800 | 0.028086 |
| M2381 | 13.658 | 0.005 | 0.0257143 |  | M1295 | 1.1059 | 0.057500 | 0.069235 |
| M2395 | 3.6232 | 0.0468 | 0.0692763 |  | M1296 | 1.2124 | 0.006400 | 0.014990 |
| M2402 | 6.2666 | 0.0127 | 0.0324716 |  | M1297 | 1.106 | 0.058300 | 0.069926 |
| M2408 | 5.339 | 0.0255 | 0.0478125 |  | M1298 | 1.0803 | 0.099900 | 0.099900 |
| M2410 | 2.9382 | 0.0769 | 0.0863866 |  | M1302 | 1.3651 | 0.000300 | 0.001392 |
| M2435 | 2.5053 | 0.089 | 0.0921396 |  | M1304 | 1.1613 | 0.017200 | 0.029598 |
| M2436 | 5.2781 | 0.0228 | 0.0448688 |  | M1305 | 1.4614 | 0.000030 | 0.000235 |
| M2440 | 5.9226 | 0.0201 | 0.041875 |  | M1307 | 1.1039 | 0.059700 | 0.070902 |
| M2441 | 3.3923 | 0.0639 | 0.0804711 |  | M1309 | 1.2303 | 0.003900 | 0.010238 |
| M2444 | 9.6897 | 0.0017 | 0.0225 |  | M1310 | 1.2204 | 0.009100 | 0.019711 |
| M2450 | 2.6565 | 0.0799 | 0.0881006 |  | M1311 | 1.1405 | 0.027700 | 0.041956 |
| M2452 | 9.0201 | 0.0031 | 0.0247277 |  | M1315 | 1.1738 | 0.013000 | 0.024895 |
| M2453 | 14.13 | 0.0006 | 0.02 |  | M1317 | 1.1967 | 0.008700 | 0.019011 |
| M2465 | 3.4512 | 0.0547 | 0.075352 |  | M1318 | 1.1967 | 0.008300 | 0.018397 |
| M2466 | 2.8014 | 0.0889 | 0.0921396 |  | M1319 | 1.1611 | 0.017900 | 0.030506 |
| M2467 | 3.1418 | 0.0731 | 0.0844906 |  | M1321 | 1.1624 | 0.016600 | 0.028887 |
| M2470 | 2.8602 | 0.0949 | 0.0961824 |  | M1322 | 1.5171 | 0.000030 | 0.000235 |
| M2473 | 11.958 | 0.0003 | 0.016875 |  | M1323 | 1.0807 | 0.097700 | 0.098536 |
|  |  |  |  |  | M1326 | 1.1726 | 0.014000 | 0.026084 |
|  |  |  |  |  | M1327 | 1.0949 | 0.073600 | 0.082080 |
|  |  |  |  |  | M1329 | 1.2164 | 0.006100 | 0.014423 |
|  |  |  |  |  | M1334 | 1.0857 | 0.088600 | 0.092248 |
|  |  |  |  |  | M1340 | 1.1008 | 0.064700 | 0.074779 |
|  |  |  |  |  | M1341 | 1.2124 | 0.006100 | 0.014423 |
|  |  |  |  |  | M1344 | 1.5676 | 0.000010 | 0.000099 |
|  |  |  |  |  | M1349 | 1.379 | 0.000100 | 0.000543 |
|  |  |  |  |  | M1355 | 1.3201 | 0.000600 | 0.002383 |
|  |  |  |  |  | M1362 | 1.1331 | 0.031900 | 0.045958 |
|  |  |  |  |  | M1363 | 1.1178 | 0.045600 | 0.059037 |
|  |  |  |  |  | M1369 | 1.2803 | 0.001800 | 0.005646 |
|  |  |  |  |  | M1380 | 1.1125 | 0.049700 | 0.062580 |
|  |  |  |  |  | M1381 | 1.1664 | 0.017800 | 0.030420 |
|  |  |  |  |  | M1382 | 1.2464 | 0.005100 | 0.012562 |
|  |  |  |  |  | M1386 | 1.159 | 0.018300 | 0.031017 |
|  |  |  |  |  | M1392 | 1.1713 | 0.014300 | 0.026248 |
|  |  |  |  |  | M1393 | 1.2501 | 0.002900 | 0.008148 |
|  |  |  |  |  | M1396 | 1.1274 | 0.037100 | 0.050792 |
|  |  |  |  |  | M1397 | 1.1453 | 0.024700 | 0.038689 |
|  |  |  |  |  | M1398 | 1.5623 | 0.000020 | 0.000171 |
|  |  |  |  |  | M1399 | 1.2786 | 0.001600 | 0.005203 |
|  |  |  |  |  | M1400 | 1.2266 | 0.004300 | 0.010962 |
|  |  |  |  |  | M1402 | 1.1809 | 0.011300 | 0.023065 |
|  |  |  |  |  | M1405 | 1.0889 | 0.084000 | 0.088954 |
|  |  |  |  |  | M1409 | 1.0925 | 0.079000 | 0.086011 |
|  |  |  |  |  | M1412 | 1.1876 | 0.026300 | 0.040329 |
|  |  |  |  |  | M1419 | 1.1321 | 0.033100 | 0.047139 |
|  |  |  |  |  | M1420 | 1.3573 | 0.000300 | 0.001392 |
|  |  |  |  |  | M1421 | 1.5989 | 0.000010 | 0.000099 |
|  |  |  |  |  | M1428 | 1.1149 | 0.052900 | 0.065282 |
|  |  |  |  |  | M1430 | 1.1307 | 0.034100 | 0.048175 |
|  |  |  |  |  | M1431 | 1.1278 | 0.036600 | 0.050330 |
|  |  |  |  |  | M1432 | 1.1393 | 0.028700 | 0.043154 |
|  |  |  |  |  | M1436 | 1.1697 | 0.014300 | 0.026248 |
|  |  |  |  |  | M1439 | 1.2732 | 0.001800 | 0.005646 |
|  |  |  |  |  | M1440 | 1.0833 | 0.092400 | 0.094819 |
|  |  |  |  |  | M1441 | 1.1811 | 0.011500 | 0.023320 |
|  |  |  |  |  | M1442 | 1.5293 | 0.000030 | 0.000235 |
|  |  |  |  |  | M1444 | 1.1436 | 0.026200 | 0.040275 |
|  |  |  |  |  | M1447 | 1.0879 | 0.086300 | 0.090462 |
|  |  |  |  |  | M1448 | 1.323 | 0.000500 | 0.002107 |
|  |  |  |  |  | M1450 | 1.2141 | 0.005700 | 0.013767 |
|  |  |  |  |  | M1452 | 1.1124 | 0.050000 | 0.062702 |
|  |  |  |  |  | M1454 | 1.1167 | 0.048100 | 0.061313 |
|  |  |  |  |  | M1455 | 1.8749 | 0.000010 | 0.000099 |
|  |  |  |  |  | M1457 | 1.1102 | 0.055100 | 0.067393 |
|  |  |  |  |  | M1462 | 1.2208 | 0.005000 | 0.012341 |
|  |  |  |  |  | M1463 | 2.0097 | 0.000010 | 0.000099 |
|  |  |  |  |  | M1465 | 1.17 | 0.014200 | 0.026248 |
|  |  |  |  |  | M1466 | 1.1919 | 0.012100 | 0.023987 |
|  |  |  |  |  | M1467 | 1.5567 | 0.000030 | 0.000235 |
|  |  |  |  |  | M1468 | 1.3394 | 0.000400 | 0.001764 |
|  |  |  |  |  | M1469 | 1.2244 | 0.004900 | 0.012191 |
|  |  |  |  |  | M1472 | 1.0977 | 0.073600 | 0.082080 |
|  |  |  |  |  | M1473 | 1.1769 | 0.012300 | 0.024037 |
|  |  |  |  |  | M1478 | 1.1351 | 0.031300 | 0.045464 |
|  |  |  |  |  | M1479 | 1.084 | 0.092900 | 0.094970 |
|  |  |  |  |  | M1481 | 1.3916 | 0.000300 | 0.001392 |
|  |  |  |  |  | M1485 | 1.0942 | 0.076700 | 0.084397 |
|  |  |  |  |  | M1488 | 1.1709 | 0.013700 | 0.025797 |
|  |  |  |  |  | M1490 | 1.3134 | 0.000800 | 0.003059 |
|  |  |  |  |  | M1491 | 1.3138 | 0.000800 | 0.003059 |
|  |  |  |  |  | M1492 | 1.1489 | 0.022300 | 0.035883 |
|  |  |  |  |  | M1496 | 1.1384 | 0.030700 | 0.044961 |
|  |  |  |  |  | M1498 | 1.1424 | 0.026300 | 0.040329 |
|  |  |  |  |  | M1499 | 1.3591 | 0.000400 | 0.001764 |
|  |  |  |  |  | M1501 | 1.1134 | 0.052500 | 0.064983 |
|  |  |  |  |  | M1503 | 1.6684 | 0.000010 | 0.000099 |
|  |  |  |  |  | M1506 | 1.1463 | 0.024500 | 0.038620 |
|  |  |  |  |  | M1509 | 1.2868 | 0.001200 | 0.004142 |
|  |  |  |  |  | M1511 | 1.3472 | 0.000400 | 0.001764 |
|  |  |  |  |  | M1512 | 1.2657 | 0.002000 | 0.006164 |
|  |  |  |  |  | M1513 | 1.7068 | 0.000010 | 0.000099 |
|  |  |  |  |  | M1514 | 1.0824 | 0.099900 | 0.099900 |
|  |  |  |  |  | M1520 | 1.1286 | 0.034800 | 0.048830 |
|  |  |  |  |  | M1521 | 1.1603 | 0.018500 | 0.031186 |
|  |  |  |  |  | M1524 | 1.1227 | 0.042100 | 0.055669 |
|  |  |  |  |  | M1525 | 1.2305 | 0.004500 | 0.011332 |
|  |  |  |  |  | M1526 | 1.7342 | 0.000010 | 0.000099 |
|  |  |  |  |  | M1527 | 1.2227 | 0.004600 | 0.011537 |
|  |  |  |  |  | M1528 | 1.54 | 0.000050 | 0.000344 |
|  |  |  |  |  | M1529 | 1.4491 | 0.000020 | 0.000171 |
|  |  |  |  |  | M1531 | 1.1245 | 0.038700 | 0.052576 |
|  |  |  |  |  | M1535 | 1.0826 | 0.097800 | 0.098536 |
|  |  |  |  |  | M1537 | 1.0842 | 0.090900 | 0.093620 |
|  |  |  |  |  | M1538 | 1.3873 | 0.000200 | 0.000999 |
|  |  |  |  |  | M1539 | 1.2182 | 0.005400 | 0.013119 |
|  |  |  |  |  | M1540 | 1.1523 | 0.021100 | 0.034626 |
|  |  |  |  |  | M1543 | 1.1044 | 0.060500 | 0.071187 |
|  |  |  |  |  | M1545 | 1.4086 | 0.000200 | 0.000999 |
|  |  |  |  |  | M1546 | 1.7605 | 0.000010 | 0.000099 |
|  |  |  |  |  | M1548 | 1.0986 | 0.066500 | 0.076432 |
|  |  |  |  |  | M1550 | 1.3764 | 0.000300 | 0.001392 |
|  |  |  |  |  | M1552 | 1.4201 | 0.000100 | 0.000543 |
|  |  |  |  |  | M1553 | 1.5636 | 0.000010 | 0.000099 |
|  |  |  |  |  | M1555 | 1.2952 | 0.001000 | 0.003602 |
|  |  |  |  |  | M1556 | 1.1768 | 0.012200 | 0.024037 |
|  |  |  |  |  | M1557 | 1.3843 | 0.000200 | 0.000999 |
|  |  |  |  |  | M1559 | 1.1156 | 0.047400 | 0.060796 |
|  |  |  |  |  | M1560 | 1.8091 | 0.000010 | 0.000099 |
|  |  |  |  |  | M1561 | 1.1492 | 0.022800 | 0.036404 |
|  |  |  |  |  | M1562 | 1.3119 | 0.000700 | 0.002736 |
|  |  |  |  |  | M1563 | 1.2136 | 0.005900 | 0.014139 |
|  |  |  |  |  | M1564 | 1.1648 | 0.017000 | 0.029377 |
|  |  |  |  |  | M1565 | 1.1651 | 0.016400 | 0.028619 |
|  |  |  |  |  | M1566 | 1.1235 | 0.039300 | 0.053100 |
|  |  |  |  |  | M1569 | 1.094 | 0.073200 | 0.081855 |
|  |  |  |  |  | M1571 | 1.3301 | 0.000500 | 0.002107 |
|  |  |  |  |  | M1572 | 1.3273 | 0.000600 | 0.002383 |
|  |  |  |  |  | M1573 | 1.1161 | 0.047000 | 0.060470 |
|  |  |  |  |  | M1574 | 1.0929 | 0.076300 | 0.084217 |
|  |  |  |  |  | M1575 | 1.518 | 0.000030 | 0.000235 |
|  |  |  |  |  | M1576 | 1.167 | 0.015000 | 0.027013 |
|  |  |  |  |  | M1577 | 1.5707 | 0.000020 | 0.000171 |
|  |  |  |  |  | M1578 | 1.1291 | 0.034900 | 0.048860 |
|  |  |  |  |  | M1579 | 1.2055 | 0.006900 | 0.015832 |
|  |  |  |  |  | M1580 | 1.1456 | 0.024800 | 0.038748 |
|  |  |  |  |  | M1582 | 1.2935 | 0.001000 | 0.003602 |
|  |  |  |  |  | M1583 | 1.7599 | 0.000010 | 0.000099 |
|  |  |  |  |  | M1586 | 1.3105 | 0.000900 | 0.003349 |
|  |  |  |  |  | M1587 | 1.1336 | 0.032000 | 0.046049 |
|  |  |  |  |  | M1592 | 1.3934 | 0.000100 | 0.000543 |
|  |  |  |  |  | M1595 | 2.2187 | 0.000010 | 0.000099 |
|  |  |  |  |  | M1597 | 1.1666 | 0.015900 | 0.028103 |
|  |  |  |  |  | M1598 | 1.1726 | 0.013700 | 0.025797 |
|  |  |  |  |  | M1599 | 2.3133 | 0.000010 | 0.000099 |
|  |  |  |  |  | M1600 | 1.1638 | 0.016300 | 0.028619 |
|  |  |  |  |  | M1601 | 1.1438 | 0.025900 | 0.039913 |
|  |  |  |  |  | M1605 | 1.3916 | 0.000100 | 0.000543 |
|  |  |  |  |  | M1607 | 1.1974 | 0.008600 | 0.018859 |
|  |  |  |  |  | M1608 | 1.2657 | 0.001900 | 0.005900 |
|  |  |  |  |  | M1609 | 1.6715 | 0.000010 | 0.000099 |
|  |  |  |  |  | M1610 | 1.6987 | 0.000010 | 0.000099 |
|  |  |  |  |  | M1611 | 1.1381 | 0.029100 | 0.043597 |
|  |  |  |  |  | M1612 | 1.0969 | 0.069100 | 0.078474 |
|  |  |  |  |  | M1616 | 1.1697 | 0.014300 | 0.026248 |
|  |  |  |  |  | M1617 | 1.234 | 0.003600 | 0.009634 |
|  |  |  |  |  | M1619 | 1.2974 | 0.001100 | 0.003872 |
|  |  |  |  |  | M1621 | 1.2749 | 0.001800 | 0.005646 |
|  |  |  |  |  | M1624 | 1.1487 | 0.023900 | 0.037819 |
|  |  |  |  |  | M1626 | 1.0885 | 0.085900 | 0.090272 |
|  |  |  |  |  | M1629 | 1.1751 | 0.012600 | 0.024355 |
|  |  |  |  |  | M1630 | 1.3232 | 0.000600 | 0.002383 |
|  |  |  |  |  | M1631 | 1.2001 | 0.007400 | 0.016701 |
|  |  |  |  |  | M1634 | 1.1123 | 0.055900 | 0.068102 |
|  |  |  |  |  | M1638 | 1.2804 | 0.001700 | 0.005443 |
|  |  |  |  |  | M1640 | 1.1858 | 0.010500 | 0.021865 |
|  |  |  |  |  | M1643 | 1.5623 | 0.000020 | 0.000171 |
|  |  |  |  |  | M1645 | 1.1247 | 0.038000 | 0.051852 |
|  |  |  |  |  | M1646 | 1.0998 | 0.067600 | 0.077337 |
|  |  |  |  |  | M1647 | 1.2292 | 0.004000 | 0.010434 |
|  |  |  |  |  | M1649 | 1.4777 | 0.000030 | 0.000235 |
|  |  |  |  |  | M1652 | 1.7371 | 0.000010 | 0.000099 |
|  |  |  |  |  | M1657 | 1.6055 | 0.000020 | 0.000171 |
|  |  |  |  |  | M1658 | 1.7582 | 0.000010 | 0.000099 |
|  |  |  |  |  | M1662 | 1.1352 | 0.030500 | 0.044921 |
|  |  |  |  |  | M1663 | 1.4172 | 0.000050 | 0.000344 |
|  |  |  |  |  | M1665 | 1.4172 | 0.000200 | 0.000999 |
|  |  |  |  |  | M1667 | 1.0821 | 0.097900 | 0.098536 |
|  |  |  |  |  | M1681 | 1.0883 | 0.086400 | 0.090490 |
|  |  |  |  |  | M1688 | 1.232 | 0.003600 | 0.009634 |
|  |  |  |  |  | M1690 | 1.4925 | 0.000030 | 0.000235 |
|  |  |  |  |  | M1691 | 1.151 | 0.021500 | 0.035005 |
|  |  |  |  |  | M1694 | 1.2585 | 0.002600 | 0.007509 |
|  |  |  |  |  | M1698 | 1.3175 | 0.000900 | 0.003349 |
|  |  |  |  |  | M1699 | 1.7558 | 0.000010 | 0.000099 |
|  |  |  |  |  | M1700 | 1.25 | 0.002800 | 0.007994 |
|  |  |  |  |  | M1704 | 1.103 | 0.062700 | 0.073150 |
|  |  |  |  |  | M1708 | 1.105 | 0.059000 | 0.070357 |
|  |  |  |  |  | M1709 | 1.1432 | 0.025700 | 0.039803 |
|  |  |  |  |  | M1710 | 1.2212 | 0.005200 | 0.012733 |
|  |  |  |  |  | M1712 | 1.0907 | 0.081400 | 0.087276 |
|  |  |  |  |  | M1715 | 1.2838 | 0.001300 | 0.004377 |
|  |  |  |  |  | M1723 | 1.2494 | 0.002500 | 0.007237 |
|  |  |  |  |  | M1725 | 1.3029 | 0.001000 | 0.003602 |
|  |  |  |  |  | M1726 | 1.2821 | 0.001400 | 0.004650 |
|  |  |  |  |  | M1729 | 1.1748 | 0.013100 | 0.024971 |
|  |  |  |  |  | M1730 | 1.0908 | 0.079500 | 0.086404 |
|  |  |  |  |  | M1741 | 2.35 | 0.000010 | 0.000099 |
|  |  |  |  |  | M1742 | 1.1036 | 0.060700 | 0.071287 |
|  |  |  |  |  | M1743 | 1.1223 | 0.042000 | 0.055669 |
|  |  |  |  |  | M1744 | 1.1494 | 0.022100 | 0.035654 |
|  |  |  |  |  | M1745 | 1.4403 | 0.000100 | 0.000543 |
|  |  |  |  |  | M1746 | 1.1637 | 0.016400 | 0.028619 |
|  |  |  |  |  | M1747 | 1.1164 | 0.046800 | 0.060338 |
|  |  |  |  |  | M1748 | 1.193 | 0.008700 | 0.019011 |
|  |  |  |  |  | M1750 | 1.1312 | 0.033200 | 0.047227 |
|  |  |  |  |  | M1751 | 1.0804 | 0.097200 | 0.098472 |
|  |  |  |  |  | M1753 | 1.1647 | 0.016200 | 0.028511 |
|  |  |  |  |  | M1754 | 1.2168 | 0.005300 | 0.012901 |
|  |  |  |  |  | M1757 | 2.0475 | 0.000010 | 0.000099 |
|  |  |  |  |  | M1758 | 1.2126 | 0.005900 | 0.014139 |
|  |  |  |  |  | M1759 | 1.384 | 0.000200 | 0.000999 |
|  |  |  |  |  | M1760 | 1.3145 | 0.000700 | 0.002736 |
|  |  |  |  |  | M1765 | 1.3624 | 0.000200 | 0.000999 |
|  |  |  |  |  | M1767 | 1.2091 | 0.006300 | 0.014784 |
|  |  |  |  |  | M1768 | 1.4157 | 0.000080 | 0.000496 |
|  |  |  |  |  | M1769 | 1.1191 | 0.044000 | 0.057689 |
|  |  |  |  |  | M1770 | 1.2555 | 0.002500 | 0.007237 |
|  |  |  |  |  | M1776 | 1.1693 | 0.014900 | 0.026990 |
|  |  |  |  |  | M1779 | 1.0874 | 0.089800 | 0.092899 |
|  |  |  |  |  | M1781 | 1.3159 | 0.001000 | 0.003602 |
|  |  |  |  |  | M1782 | 1.1964 | 0.008600 | 0.018859 |
|  |  |  |  |  | M1785 | 1.2536 | 0.002500 | 0.007237 |
|  |  |  |  |  | M1787 | 1.093 | 0.077400 | 0.085016 |
|  |  |  |  |  | M1788 | 1.0992 | 0.068700 | 0.078163 |
|  |  |  |  |  | M1789 | 1.1339 | 0.031900 | 0.045958 |
|  |  |  |  |  | M1790 | 1.1667 | 0.015600 | 0.027851 |
|  |  |  |  |  | M1791 | 1.1386 | 0.031700 | 0.045884 |
|  |  |  |  |  | M1793 | 1.1811 | 0.011400 | 0.023193 |
|  |  |  |  |  | M1797 | 1.2428 | 0.003300 | 0.009026 |
|  |  |  |  |  | M1799 | 1.1886 | 0.011000 | 0.022602 |
|  |  |  |  |  | M1800 | 1.9597 | 0.000010 | 0.000099 |
|  |  |  |  |  | M1801 | 1.7342 | 0.000010 | 0.000099 |
|  |  |  |  |  | M1802 | 1.1434 | 0.024700 | 0.038689 |
|  |  |  |  |  | M1803 | 1.1809 | 0.011000 | 0.022602 |
|  |  |  |  |  | M1805 | 1.2256 | 0.004000 | 0.010434 |
|  |  |  |  |  | M1806 | 1.1524 | 0.021600 | 0.035075 |
|  |  |  |  |  | M1808 | 1.1879 | 0.009900 | 0.021040 |
|  |  |  |  |  | M1809 | 1.1236 | 0.040400 | 0.054173 |
|  |  |  |  |  | M1811 | 1.1347 | 0.031200 | 0.045464 |
|  |  |  |  |  | M1812 | 1.2942 | 0.001200 | 0.004142 |
|  |  |  |  |  | M1816 | 1.2076 | 0.006600 | 0.015313 |
|  |  |  |  |  | M1817 | 1.3615 | 0.000400 | 0.001764 |
|  |  |  |  |  | M1818 | 1.1591 | 0.019200 | 0.032147 |
|  |  |  |  |  | M1824 | 1.1136 | 0.049200 | 0.062266 |
|  |  |  |  |  | M1825 | 1.2307 | 0.004200 | 0.010752 |
|  |  |  |  |  | M1826 | 1.2069 | 0.006500 | 0.015167 |
|  |  |  |  |  | M1827 | 1.1782 | 0.012300 | 0.024037 |
|  |  |  |  |  | M1828 | 1.144 | 0.026600 | 0.040488 |
|  |  |  |  |  | M1829 | 1.1183 | 0.044600 | 0.058291 |
|  |  |  |  |  | M1830 | 1.0892 | 0.085100 | 0.089735 |
|  |  |  |  |  | M1831 | 1.6221 | 0.000010 | 0.000099 |
|  |  |  |  |  | M1832 | 1.4217 | 0.000200 | 0.000999 |
|  |  |  |  |  | M1833 | 1.1145 | 0.049400 | 0.062392 |
|  |  |  |  |  | M1835 | 1.7525 | 0.000010 | 0.000099 |
|  |  |  |  |  | M1840 | 1.3974 | 0.000100 | 0.000543 |
|  |  |  |  |  | M1844 | 1.0951 | 0.076300 | 0.084217 |
|  |  |  |  |  | M1846 | 1.1787 | 0.012100 | 0.023987 |
|  |  |  |  |  | M1847 | 1.0904 | 0.082200 | 0.087722 |
|  |  |  |  |  | M1848 | 1.2257 | 0.004500 | 0.011332 |
|  |  |  |  |  | M1850 | 1.1503 | 0.022600 | 0.036224 |
|  |  |  |  |  | M1852 | 1.156 | 0.019700 | 0.032719 |
|  |  |  |  |  | M1854 | 1.4993 | 0.000030 | 0.000235 |
|  |  |  |  |  | M1855 | 1.5504 | 0.000050 | 0.000344 |
|  |  |  |  |  | M1857 | 1.083 | 0.095000 | 0.096717 |
|  |  |  |  |  | M1858 | 1.2595 | 0.002000 | 0.006164 |
|  |  |  |  |  | M1860 | 1.2009 | 0.007100 | 0.016171 |
|  |  |  |  |  | M1861 | 1.1058 | 0.059000 | 0.070357 |
|  |  |  |  |  | M1863 | 1.1417 | 0.027000 | 0.040996 |
|  |  |  |  |  | M1867 | 1.0981 | 0.071100 | 0.080157 |
|  |  |  |  |  | M1869 | 1.3346 | 0.000600 | 0.002383 |
|  |  |  |  |  | M1871 | 1.084 | 0.096200 | 0.097698 |
|  |  |  |  |  | M1874 | 1.1304 | 0.034600 | 0.048715 |
|  |  |  |  |  | M1875 | 1.2281 | 0.004100 | 0.010561 |
|  |  |  |  |  | M1876 | 1.2281 | 0.003900 | 0.010238 |
|  |  |  |  |  | M1878 | 1.4834 | 0.000060 | 0.000395 |
|  |  |  |  |  | M1884 | 1.4031 | 0.000100 | 0.000543 |
|  |  |  |  |  | M1885 | 1.3627 | 0.000300 | 0.001392 |
|  |  |  |  |  | M1887 | 1.1449 | 0.024800 | 0.038748 |
|  |  |  |  |  | M1892 | 1.3616 | 0.000300 | 0.001392 |
|  |  |  |  |  | M1893 | 1.3484 | 0.000300 | 0.001392 |
|  |  |  |  |  | M1894 | 1.11 | 0.054500 | 0.066923 |
|  |  |  |  |  | M1895 | 1.1177 | 0.044000 | 0.057689 |
|  |  |  |  |  | M1896 | 1.1833 | 0.011000 | 0.022602 |
|  |  |  |  |  | M1898 | 1.2156 | 0.005500 | 0.013336 |
|  |  |  |  |  | M1901 | 1.1747 | 0.013100 | 0.024971 |
|  |  |  |  |  | M1903 | 1.1084 | 0.056100 | 0.068200 |
|  |  |  |  |  | M1907 | 1.6968 | 0.000010 | 0.000099 |
|  |  |  |  |  | M1908 | 1.7204 | 0.000010 | 0.000099 |
|  |  |  |  |  | M1909 | 1.3259 | 0.000600 | 0.002383 |
|  |  |  |  |  | M1910 | 1.097 | 0.071100 | 0.080157 |
|  |  |  |  |  | M1911 | 1.1934 | 0.009100 | 0.019711 |
|  |  |  |  |  | M1913 | 1.4101 | 0.000100 | 0.000543 |
|  |  |  |  |  | M1914 | 1.0913 | 0.081500 | 0.087276 |
|  |  |  |  |  | M1915 | 1.1321 | 0.033600 | 0.047632 |
|  |  |  |  |  | M1916 | 1.0923 | 0.078800 | 0.085869 |
|  |  |  |  |  | M1919 | 1.3315 | 0.000600 | 0.002383 |
|  |  |  |  |  | M1920 | 1.2945 | 0.000900 | 0.003349 |
|  |  |  |  |  | M1924 | 1.6471 | 0.000020 | 0.000171 |
|  |  |  |  |  | M1925 | 1.6766 | 0.000010 | 0.000099 |
|  |  |  |  |  | M1926 | 1.2081 | 0.006500 | 0.015167 |
|  |  |  |  |  | M1927 | 1.0835 | 0.094900 | 0.096695 |
|  |  |  |  |  | M1928 | 1.1723 | 0.012900 | 0.024780 |
|  |  |  |  |  | M1929 | 1.2731 | 0.001600 | 0.005203 |
|  |  |  |  |  | M1933 | 1.3896 | 0.000300 | 0.001392 |
|  |  |  |  |  | M1934 | 1.3648 | 0.000300 | 0.001392 |
|  |  |  |  |  | M1935 | 1.4187 | 0.000100 | 0.000543 |
|  |  |  |  |  | M1936 | 1.6398 | 0.000010 | 0.000099 |
|  |  |  |  |  | M1939 | 2.4727 | 0.000010 | 0.000099 |
|  |  |  |  |  | M1941 | 1.2887 | 0.001300 | 0.004377 |
|  |  |  |  |  | M1943 | 1.2108 | 0.006000 | 0.014241 |
|  |  |  |  |  | M1948 | 1.1284 | 0.036100 | 0.049864 |
|  |  |  |  |  | M1949 | 1.1316 | 0.033000 | 0.047051 |
|  |  |  |  |  | M1950 | 1.178 | 0.012100 | 0.023987 |
|  |  |  |  |  | M1951 | 1.1073 | 0.057000 | 0.068900 |
|  |  |  |  |  | M1952 | 1.212 | 0.006000 | 0.014241 |
|  |  |  |  |  | M1953 | 1.656 | 0.000010 | 0.000099 |
|  |  |  |  |  | M1956 | 1.1062 | 0.057900 | 0.069514 |
|  |  |  |  |  | M1959 | 1.1988 | 0.008600 | 0.018859 |
|  |  |  |  |  | M1960 | 2.1593 | 0.000010 | 0.000099 |
|  |  |  |  |  | M1965 | 1.2572 | 0.002400 | 0.007097 |
|  |  |  |  |  | M1966 | 1.274 | 0.001500 | 0.004917 |
|  |  |  |  |  | M1967 | 1.1044 | 0.064100 | 0.074224 |
|  |  |  |  |  | M1970 | 1.1995 | 0.007000 | 0.016002 |
|  |  |  |  |  | M1971 | 1.1706 | 0.014300 | 0.026248 |
|  |  |  |  |  | M1975 | 1.1776 | 0.012700 | 0.024472 |
|  |  |  |  |  | M1976 | 1.1835 | 0.010900 | 0.022546 |
|  |  |  |  |  | M1977 | 1.2524 | 0.002700 | 0.007726 |
|  |  |  |  |  | M1978 | 1.1789 | 0.012100 | 0.023987 |
|  |  |  |  |  | M1980 | 1.1383 | 0.030200 | 0.044598 |
|  |  |  |  |  | M1981 | 1.2483 | 0.002500 | 0.007237 |
|  |  |  |  |  | M1982 | 1.1297 | 0.036100 | 0.049864 |
|  |  |  |  |  | M1985 | 1.1883 | 0.010100 | 0.021282 |
|  |  |  |  |  | M1986 | 1.3439 | 0.000400 | 0.001764 |
|  |  |  |  |  | M1988 | 1.2273 | 0.004200 | 0.010752 |
|  |  |  |  |  | M1991 | 1.367 | 0.000500 | 0.002107 |
|  |  |  |  |  | M1994 | 1.2082 | 0.006300 | 0.014784 |
|  |  |  |  |  | M1997 | 1.5235 | 0.000050 | 0.000344 |
|  |  |  |  |  | M2003 | 1.1732 | 0.013000 | 0.024895 |
|  |  |  |  |  | M2006 | 1.1666 | 0.015900 | 0.028103 |
|  |  |  |  |  | M2007 | 1.1382 | 0.028100 | 0.042355 |
|  |  |  |  |  | M2009 | 1.2476 | 0.003100 | 0.008593 |
|  |  |  |  |  | M2010 | 1.1861 | 0.010000 | 0.021143 |
|  |  |  |  |  | M2012 | 1.3898 | 0.000200 | 0.000999 |
|  |  |  |  |  | M2015 | 1.2708 | 0.001800 | 0.005646 |
|  |  |  |  |  | M2016 | 1.2327 | 0.003600 | 0.009634 |
|  |  |  |  |  | M2018 | 1.0809 | 0.097500 | 0.098536 |
|  |  |  |  |  | M2022 | 1.102 | 0.063100 | 0.073478 |
|  |  |  |  |  | M2024 | 1.0864 | 0.087500 | 0.091410 |
|  |  |  |  |  | M2027 | 1.0854 | 0.087700 | 0.091542 |
|  |  |  |  |  | M2028 | 1.3722 | 0.000200 | 0.000999 |
|  |  |  |  |  | M2029 | 1.0915 | 0.079800 | 0.086502 |
|  |  |  |  |  | M2032 | 1.0888 | 0.083300 | 0.088515 |
|  |  |  |  |  | M2033 | 1.1354 | 0.030500 | 0.044921 |
|  |  |  |  |  | M2035 | 1.0885 | 0.082400 | 0.087785 |
|  |  |  |  |  | M2037 | 1.1939 | 0.008900 | 0.019414 |
|  |  |  |  |  | M2038 | 1.1082 | 0.054800 | 0.067092 |
|  |  |  |  |  | M2040 | 1.4252 | 0.000080 | 0.000496 |
|  |  |  |  |  | M2041 | 1.381 | 0.000200 | 0.000999 |
|  |  |  |  |  | M2042 | 1.6374 | 0.000010 | 0.000099 |
|  |  |  |  |  | M2043 | 1.1127 | 0.049700 | 0.062580 |
|  |  |  |  |  | M2048 | 1.1325 | 0.032200 | 0.046176 |
|  |  |  |  |  | M2049 | 1.3163 | 0.000800 | 0.003059 |
|  |  |  |  |  | M2050 | 1.1137 | 0.048200 | 0.061377 |
|  |  |  |  |  | M2052 | 1.1895 | 0.009700 | 0.020721 |
|  |  |  |  |  | M2054 | 1.1766 | 0.012500 | 0.024237 |
|  |  |  |  |  | M2055 | 1.1944 | 0.008600 | 0.018859 |
|  |  |  |  |  | M2057 | 1.208 | 0.006000 | 0.014241 |
|  |  |  |  |  | M2058 | 1.1543 | 0.019900 | 0.032963 |
|  |  |  |  |  | M2059 | 1.1885 | 0.009800 | 0.020899 |
|  |  |  |  |  | M2060 | 1.1685 | 0.015000 | 0.027013 |
|  |  |  |  |  | M2061 | 1.2678 | 0.002100 | 0.006377 |
|  |  |  |  |  | M2063 | 1.1611 | 0.017100 | 0.029467 |
|  |  |  |  |  | M2076 | 1.1095 | 0.053800 | 0.066129 |
|  |  |  |  |  | M2078 | 1.1167 | 0.045200 | 0.058826 |
|  |  |  |  |  | M2079 | 1.0905 | 0.082400 | 0.087785 |
|  |  |  |  |  | M2084 | 1.1273 | 0.036000 | 0.049837 |
|  |  |  |  |  | M2085 | 1.0844 | 0.092600 | 0.094819 |
|  |  |  |  |  | M2086 | 1.1357 | 0.030700 | 0.044961 |
|  |  |  |  |  | M2092 | 1.2668 | 0.001800 | 0.005646 |
|  |  |  |  |  | M2093 | 1.1152 | 0.048500 | 0.061632 |
|  |  |  |  |  | M2094 | 1.277 | 0.001300 | 0.004377 |
|  |  |  |  |  | M2096 | 1.3022 | 0.000900 | 0.003349 |
|  |  |  |  |  | M2097 | 1.0825 | 0.099400 | 0.099722 |
|  |  |  |  |  | M2100 | 1.898 | 0.000010 | 0.000099 |
|  |  |  |  |  | M2101 | 1.4863 | 0.000030 | 0.000235 |
|  |  |  |  |  | M2103 | 1.1102 | 0.052000 | 0.064622 |
|  |  |  |  |  | M2107 | 1.0957 | 0.071900 | 0.080912 |
|  |  |  |  |  | M2110 | 1.1508 | 0.023200 | 0.036805 |
|  |  |  |  |  | M2113 | 1.5296 | 0.000040 | 0.000295 |
|  |  |  |  |  | M2116 | 1.1732 | 0.013800 | 0.025867 |
|  |  |  |  |  | M2117 | 1.1741 | 0.012500 | 0.024237 |
|  |  |  |  |  | M2118 | 1.0995 | 0.070800 | 0.080038 |
|  |  |  |  |  | M2121 | 1.1018 | 0.063200 | 0.073526 |
|  |  |  |  |  | M2122 | 1.0928 | 0.077700 | 0.085120 |
|  |  |  |  |  | M2126 | 1.1375 | 0.029200 | 0.043694 |
|  |  |  |  |  | M2127 | 1.1149 | 0.050100 | 0.062764 |
|  |  |  |  |  | M2128 | 1.0942 | 0.080900 | 0.087010 |
|  |  |  |  |  | M2131 | 1.0936 | 0.075800 | 0.083929 |
|  |  |  |  |  | M2133 | 1.6048 | 0.000020 | 0.000171 |
|  |  |  |  |  | M2134 | 1.1005 | 0.064200 | 0.074271 |
|  |  |  |  |  | M2138 | 1.2786 | 0.001400 | 0.004650 |
|  |  |  |  |  | M2144 | 1.559 | 0.000010 | 0.000099 |
|  |  |  |  |  | M2150 | 1.084 | 0.093200 | 0.095198 |
|  |  |  |  |  | M2151 | 1.5828 | 0.000010 | 0.000099 |
|  |  |  |  |  | M2152 | 1.8348 | 0.000010 | 0.000099 |
|  |  |  |  |  | M2154 | 1.3329 | 0.000500 | 0.002107 |
|  |  |  |  |  | M2165 | 1.2148 | 0.005800 | 0.013954 |
|  |  |  |  |  | M2166 | 1.185 | 0.010800 | 0.022377 |
|  |  |  |  |  | M2167 | 1.1221 | 0.041500 | 0.055170 |
|  |  |  |  |  | M2169 | 1.0963 | 0.070400 | 0.079731 |
|  |  |  |  |  | M2172 | 1.0852 | 0.090300 | 0.093235 |
|  |  |  |  |  | M2173 | 1.1919 | 0.013100 | 0.024971 |
|  |  |  |  |  | M2176 | 1.1129 | 0.050000 | 0.062702 |
|  |  |  |  |  | M2177 | 1.0993 | 0.068600 | 0.078163 |
|  |  |  |  |  | M2178 | 1.1663 | 0.014800 | 0.026887 |
|  |  |  |  |  | M2179 | 1.4453 | 0.000070 | 0.000447 |
|  |  |  |  |  | M2181 | 1.0827 | 0.095200 | 0.096841 |
|  |  |  |  |  | M2182 | 1.1785 | 0.011700 | 0.023648 |
|  |  |  |  |  | M2183 | 1.1254 | 0.038900 | 0.052790 |
|  |  |  |  |  | M2184 | 1.1912 | 0.010000 | 0.021143 |
|  |  |  |  |  | M2185 | 1.1707 | 0.014100 | 0.026231 |
|  |  |  |  |  | M2186 | 1.1174 | 0.044700 | 0.058298 |
|  |  |  |  |  | M2188 | 1.2944 | 0.001100 | 0.003872 |
|  |  |  |  |  | M2190 | 1.1056 | 0.061800 | 0.072510 |
|  |  |  |  |  | M2191 | 1.4049 | 0.000100 | 0.000543 |
|  |  |  |  |  | M2193 | 1.0887 | 0.085900 | 0.090272 |
|  |  |  |  |  | M2198 | 1.1218 | 0.040100 | 0.053946 |
|  |  |  |  |  | M2205 | 1.152 | 0.022700 | 0.036291 |
|  |  |  |  |  | M2206 | 1.1771 | 0.012300 | 0.024037 |
|  |  |  |  |  | M2208 | 1.2653 | 0.002100 | 0.006377 |
|  |  |  |  |  | M2211 | 1.2731 | 0.001900 | 0.005900 |
|  |  |  |  |  | M2212 | 1.1024 | 0.063600 | 0.073852 |
|  |  |  |  |  | M2213 | 1.0875 | 0.089900 | 0.092899 |
|  |  |  |  |  | M2215 | 1.3594 | 0.000200 | 0.000999 |
|  |  |  |  |  | M2216 | 1.5702 | 0.000010 | 0.000099 |
|  |  |  |  |  | M2217 | 1.7587 | 0.000010 | 0.000099 |
|  |  |  |  |  | M2218 | 1.5673 | 0.000070 | 0.000447 |
|  |  |  |  |  | M2219 | 1.1034 | 0.060300 | 0.071086 |
|  |  |  |  |  | M2220 | 1.6909 | 0.000010 | 0.000099 |
|  |  |  |  |  | M2222 | 1.1504 | 0.021400 | 0.034888 |
|  |  |  |  |  | M2224 | 1.0898 | 0.082500 | 0.087816 |
|  |  |  |  |  | M2227 | 1.1034 | 0.060600 | 0.071237 |
|  |  |  |  |  | M2228 | 1.5397 | 0.000010 | 0.000099 |
|  |  |  |  |  | M2231 | 1.0898 | 0.083900 | 0.088954 |
|  |  |  |  |  | M2232 | 1.1249 | 0.036800 | 0.050493 |
|  |  |  |  |  | M2234 | 1.1348 | 0.031300 | 0.045464 |
|  |  |  |  |  | M2235 | 1.1916 | 0.010000 | 0.021143 |
|  |  |  |  |  | M2239 | 1.2092 | 0.006300 | 0.014784 |
|  |  |  |  |  | M2240 | 1.4661 | 0.000040 | 0.000295 |
|  |  |  |  |  | M2242 | 1.1153 | 0.047500 | 0.060798 |
|  |  |  |  |  | M2243 | 1.1313 | 0.033600 | 0.047632 |
|  |  |  |  |  | M2245 | 1.1412 | 0.027300 | 0.041401 |
|  |  |  |  |  | M2248 | 1.0968 | 0.075700 | 0.083929 |
|  |  |  |  |  | M2250 | 1.1356 | 0.029900 | 0.044473 |
|  |  |  |  |  | M2251 | 1.1643 | 0.016200 | 0.028511 |
|  |  |  |  |  | M2257 | 1.1938 | 0.009200 | 0.019824 |
|  |  |  |  |  | M2259 | 1.4165 | 0.000080 | 0.000496 |
|  |  |  |  |  | M2262 | 1.1891 | 0.010200 | 0.021384 |
|  |  |  |  |  | M2263 | 1.1588 | 0.018900 | 0.031688 |
|  |  |  |  |  | M2264 | 1.2326 | 0.004100 | 0.010561 |
|  |  |  |  |  | M2266 | 1.0869 | 0.086000 | 0.090300 |
|  |  |  |  |  | M2267 | 1.0892 | 0.082700 | 0.087953 |
|  |  |  |  |  | M2272 | 1.8751 | 0.000010 | 0.000099 |
|  |  |  |  |  | M2274 | 1.1734 | 0.014400 | 0.026393 |
|  |  |  |  |  | M2276 | 1.1375 | 0.029000 | 0.043500 |
|  |  |  |  |  | M2277 | 1.093 | 0.073800 | 0.082155 |
|  |  |  |  |  | M2278 | 1.2006 | 0.007300 | 0.016505 |
|  |  |  |  |  | M2279 | 1.105 | 0.058800 | 0.070254 |
|  |  |  |  |  | M2280 | 1.5478 | 0.000030 | 0.000235 |
|  |  |  |  |  | M2282 | 1.2513 | 0.002400 | 0.007097 |
|  |  |  |  |  | M2287 | 1.0943 | 0.073800 | 0.082155 |
|  |  |  |  |  | M2288 | 1.3771 | 0.000300 | 0.001392 |
|  |  |  |  |  | M2290 | 1.0825 | 0.098900 | 0.099381 |
|  |  |  |  |  | M2294 | 1.1245 | 0.039100 | 0.052945 |
|  |  |  |  |  | M2295 | 1.188 | 0.010200 | 0.021384 |
|  |  |  |  |  | M2301 | 1.1193 | 0.043700 | 0.057478 |
|  |  |  |  |  | M2302 | 1.4278 | 0.000100 | 0.000543 |
|  |  |  |  |  | M2303 | 1.3681 | 0.000200 | 0.000999 |
|  |  |  |  |  | M2305 | 1.2055 | 0.010400 | 0.021730 |
|  |  |  |  |  | M2307 | 1.0949 | 0.073000 | 0.081852 |
|  |  |  |  |  | M2311 | 1.1286 | 0.036600 | 0.050330 |
|  |  |  |  |  | M2313 | 1.1039 | 0.060500 | 0.071187 |
|  |  |  |  |  | M2314 | 1.1326 | 0.034200 | 0.048262 |
|  |  |  |  |  | M2315 | 1.1333 | 0.032100 | 0.046086 |
|  |  |  |  |  | M2316 | 1.1873 | 0.010400 | 0.021730 |
|  |  |  |  |  | M2317 | 1.1081 | 0.053800 | 0.066129 |
|  |  |  |  |  | M2319 | 1.3277 | 0.000600 | 0.002383 |
|  |  |  |  |  | M2320 | 1.9526 | 0.000010 | 0.000099 |
|  |  |  |  |  | M2321 | 1.1138 | 0.050800 | 0.063513 |
|  |  |  |  |  | M2323 | 1.5148 | 0.000030 | 0.000235 |
|  |  |  |  |  | M2324 | 1.3192 | 0.000600 | 0.002383 |
|  |  |  |  |  | M2325 | 1.166 | 0.015700 | 0.027989 |
|  |  |  |  |  | M2335 | 1.1402 | 0.027800 | 0.042056 |
|  |  |  |  |  | M2338 | 1.1696 | 0.014300 | 0.026248 |
|  |  |  |  |  | M2339 | 1.1267 | 0.035300 | 0.049253 |
|  |  |  |  |  | M2340 | 1.357 | 0.000200 | 0.000999 |
|  |  |  |  |  | M2347 | 1.5887 | 0.000020 | 0.000171 |
|  |  |  |  |  | M2359 | 1.2039 | 0.007700 | 0.017283 |
|  |  |  |  |  | M2361 | 2.2074 | 0.000010 | 0.000099 |
|  |  |  |  |  | M2363 | 1.1175 | 0.044700 | 0.058298 |
|  |  |  |  |  | M2364 | 1.2756 | 0.001700 | 0.005443 |
|  |  |  |  |  | M2368 | 1.1367 | 0.030200 | 0.044598 |
|  |  |  |  |  | M2369 | 1.3398 | 0.000400 | 0.001764 |
|  |  |  |  |  | M2371 | 1.3863 | 0.000200 | 0.000999 |
|  |  |  |  |  | M2372 | 1.8522 | 0.000010 | 0.000099 |
|  |  |  |  |  | M2373 | 1.413 | 0.000100 | 0.000543 |
|  |  |  |  |  | M2378 | 1.1499 | 0.021700 | 0.035191 |
|  |  |  |  |  | M2380 | 1.0974 | 0.076400 | 0.084217 |
|  |  |  |  |  | M2381 | 1.4819 | 0.000040 | 0.000295 |
|  |  |  |  |  | M2385 | 1.154 | 0.021000 | 0.034554 |
|  |  |  |  |  | M2389 | 1.0859 | 0.089800 | 0.092899 |
|  |  |  |  |  | M2390 | 1.1566 | 0.021600 | 0.035075 |
|  |  |  |  |  | M2392 | 1.0977 | 0.068800 | 0.078205 |
|  |  |  |  |  | M2393 | 1.0924 | 0.079100 | 0.086045 |
|  |  |  |  |  | M2394 | 1.1566 | 0.019400 | 0.032351 |
|  |  |  |  |  | M2399 | 1.1365 | 0.030200 | 0.044598 |
|  |  |  |  |  | M2402 | 1.5906 | 0.000010 | 0.000099 |
|  |  |  |  |  | M2403 | 1.1206 | 0.043600 | 0.057407 |
|  |  |  |  |  | M2404 | 1.0914 | 0.079900 | 0.086535 |
|  |  |  |  |  | M2408 | 1.0995 | 0.068500 | 0.078151 |
|  |  |  |  |  | M2411 | 1.1067 | 0.058500 | 0.070030 |
|  |  |  |  |  | M2413 | 1.1443 | 0.025100 | 0.039069 |
|  |  |  |  |  | M2415 | 1.1019 | 0.066800 | 0.076634 |
|  |  |  |  |  | M2420 | 1.1558 | 0.018500 | 0.031186 |
|  |  |  |  |  | M2421 | 1.1189 | 0.045600 | 0.059037 |
|  |  |  |  |  | M2422 | 1.1769 | 0.012700 | 0.024472 |
|  |  |  |  |  | M2428 | 1.0903 | 0.082100 | 0.087722 |
|  |  |  |  |  | M2431 | 1.1121 | 0.051200 | 0.063884 |
|  |  |  |  |  | M2435 | 1.2192 | 0.005200 | 0.012733 |
|  |  |  |  |  | M2436 | 1.1843 | 0.010200 | 0.021384 |
|  |  |  |  |  | M2438 | 1.1428 | 0.026400 | 0.040332 |
|  |  |  |  |  | M2439 | 1.193 | 0.008400 | 0.018585 |
|  |  |  |  |  | M2440 | 1.1998 | 0.007700 | 0.017283 |
|  |  |  |  |  | M2441 | 1.1796 | 0.012200 | 0.024037 |
|  |  |  |  |  | M2442 | 1.1284 | 0.035700 | 0.049727 |
|  |  |  |  |  | M2445 | 1.1695 | 0.014800 | 0.026887 |
|  |  |  |  |  | M2447 | 1.0962 | 0.072800 | 0.081702 |
|  |  |  |  |  | M2450 | 1.1764 | 0.012100 | 0.023987 |
|  |  |  |  |  | M2451 | 1.1891 | 0.009600 | 0.020579 |
|  |  |  |  |  | M2452 | 1.2623 | 0.002200 | 0.006616 |
|  |  |  |  |  | M2453 | 1.6273 | 0.000010 | 0.000099 |
|  |  |  |  |  | M2455 | 1.0922 | 0.080000 | 0.086568 |
|  |  |  |  |  | M2457 | 1.0933 | 0.078100 | 0.085331 |
|  |  |  |  |  | M2458 | 1.2594 | 0.002500 | 0.007237 |
|  |  |  |  |  | M2461 | 1.163 | 0.017900 | 0.030506 |
|  |  |  |  |  | M2464 | 1.101 | 0.063000 | 0.073431 |
|  |  |  |  |  | M2468 | 1.1076 | 0.057900 | 0.069514 |
|  |  |  |  |  | M2470 | 1.1274 | 0.035900 | 0.049810 |
|  |  |  |  |  | M2473 | 1.2559 | 0.002500 | 0.007237 |
|  |  |  |  |  | M2475 | 1.1755 | 0.012100 | 0.023987 |

**TABLE S13** Results of AMOVA with respect to *Phragmites australis* within and among habitats based on MSAP banding patterns (A. Methylation-susceptible loci (MSL); B. Nonmethylated loci (NML)).

| Source | d.f. | SS | Variance components | % variation | *P* value |
| --- | --- | --- | --- | --- | --- |
| A. MSL |  |  |  |  |  |
| Among habitats | 3 | 2232 | 22.34 | 6.99 |  |
| Within habitats | 76 | 22589 | 297.2 | 93.01 |  |
| Total | 79 | 24821 | 319.54 |  |  |
| Phi_ST=0.06992 |  |  |  |  | *P*<0.001 |
|  | |  |  |  |  |
| B. NML |  |  |  |  |  |
| Among habitats | 3 | 509.1 | 5.642 | 9.03 |  |
| Within habitats | 76 | 4321 | 56.86 | 90.97 |  |
| Total | 79 | 4830 | 62.502 |  |  |
| Phi_ST=0.09027 |  |  |  |  | *P*<0.001 |

**TABLE S14** Nei's genetic distance and Nei's genetic identity among *Phragmites australis* in 4 different habitats based on AFLPs (A.) and methylation-susceptible loci (MSL) of MSAP markers (B.).

A.

| Habitat | H1 | H2 | H3 | H4 |
| --- | --- | --- | --- | --- |
| H1 |  | 0.957 | 0.967 | 0.930 |
| H2 | 0.044 |  | 0.973 | 0.939 |
| H3 | 0.033 | 0.028 |  | 0.932 |
| H4 | 0.073 | 0.063 | 0.071 |  |

B.

| Habitat | H1 | H2 | H3 | H4 |
| --- | --- | --- | --- | --- |
| H1 |  | 0.967 | 0.958 | 0.934 |
| H2 | 0.034 |  | 0.951 | 0.938 |
| H3 | 0.043 | 0.050 |  | 0.953 |
| H4 | 0.068 | 0.064 | 0.048 |  |

Note: Nei's genetic distance is shown below the diagonal. Nei's genetic identity is shown above the diagonal.

**TABLE S15** Locus-by-locus AMOVA and the proportion of *Phragmites australis* individuals with methylation at MSAP loci between habitats 1 and 3 (A) and habitats 2 and 3 (B).

A.

|  |  |  | Proportion methylated | |
| --- | --- | --- | --- | --- |
| Locus | F_ST_ | *P*-value | L1 | L3 |
| 153 | 0.75 | 0.00293 | 0.7778 | 0 |
| 525 | 0.625 | 0.00978 | 0 | 0.6667 |
| 633 | 0.75 | 0.00098 | 1 | 0.2222 |
| 645 | 0.875 | 0 | 0 | 0.8889 |
| 651 | 0.75 | 0.00196 | 0.2222 | 1 |
| 1122 | 0.57328 | 0.00978 | 0.1111 | 0.7778 |

B.

|  |  |  | Proportion methylated | |
| --- | --- | --- | --- | --- |
| Locus | F_ST_ | *P*-value | L2 | L3 |
| 58 | 0.64286 | 0.00293 | 0.5714 | 0 |
| 94 | 0.6256 | 0.00196 | 0.7143 | 0.0667 |
| 305 | 0.63351 | 0.00489 | 0 | 0.7333 |
| 380 | 0.6256 | 0.00391 | 0.7143 | 0.0667 |
| 415 | 0.77196 | 0.00196 | 0.1429 | 0.9333 |
| 422 | 0.55128 | 0.00489 | 0 | 0.6667 |
| 487 | 0.6256 | 0.00391 | 0.7143 | 0.0667 |
| 525 | 0.55128 | 0.00684 | 0 | 0.6667 |
| 584 | 0.54976 | 0.00684 | 0.8571 | 0.2 |
| 590 | 0.6256 | 0.00782 | 0.7143 | 0.0667 |
| 647 | 0.65726 | 0.00391 | 0.1429 | 0.8667 |
| 653 | 0.64286 | 0.00587 | 0.4286 | 1 |
| 663 | 0.55128 | 0.00782 | 0 | 0.6667 |
| 908 | 0.64286 | 0.00684 | 0.5714 | 0 |
| 1477 | 0.54976 | 0.00587 | 0.8571 | 0.2 |
| 2087 | 0.77196 | 0.00098 | 0.8571 | 0.0667 |
| 2100 | 0.54976 | 0.00782 | 0.8571 | 0.2 |
| 2101 | 0.89437 | 0 | 0.8571 | 0 |
| 2132 | 0.5513 | 0.0029 | 0 | 0.6667 |
| 2214 | 0.6256 | 0.00391 | 0.7143 | 0.0667 |
| 2279 | 0.54976 | 0.00489 | 0.8571 | 0.2 |
| 2499 | 0.55128 | 0.00587 | 1 | 0.3333 |
| 2857 | 0.64286 | 0.00293 | 0.5714 | 0 |

**TABLE S16** Correlations between characteristics of *Phragmites australis* in 3 heterogeneous habitats (H1, 2, and 3) and soil characteristics in the field (R^2^ and asterisk denote statistical significance (α=0.05))

|  | Soil moisture (%) | pH | Conductivity  (us cm^-1^) | Content of total P (g kg^-1^) | Content of total N (g kg^-1^) | Organic matter  (g kg^-1^) | NH4-N (mg kg^-1^) | NO3-N (mg kg^-1^) | Cl^-^  (mg L^-1^) | SO_4_^2-^  (mg L^-1^) | Na^+^  (mg L^-1^) | K^+^  (mg L^-1^) | Mg^2+^  (mg L^-1^) | Ca^2+^  (mg L^-1^) |
| --- | --- | --- | --- | --- | --- | --- | --- | --- | --- | --- | --- | --- | --- | --- |
| Height (cm) | 0.865  * | 0.982  * | 0.954  * | 0.844  * | 0.992  * | 0.991  * | 0.765  * | 0.815  * | 0.971  * | 0.768  * | 0.973  * | 0.986  * | 0.892  * | 0.998  * |
| Stem diameter (cm) | 0.965  * | 0.953  * | 0.951  * | 0.975  * | 0.919  * | 0.984  * | 0.563 | 0.796  * | 0.912  * | 0.356 | 0.930  * | 0.912  * | 0.986  * | 0.986  * |
| Leaf length (cm) | 0.938  * | 0.970  * | 0.900  * | 0.918  * | 0.963  * | 0.982  * | 0.657  * | 0.698  * | 0.936  * | 0.508  * | 0.917  * | 0.958  * | 0.954  * | 0.980  * |
| Leaf width (cm） | 0.898  * | 0.986  * | 0.806  * | 0.812  * | 0.884  * | 0.959  * | 0.461 | 0.596 | 0.913  * | 0.042 | 0.825  * | 0.850  * | 0.913  * | 0.967  * |
| Node number | 0.822  * | 0.861  * | 0.740  * | 0.815  * | 0.797  * | 0.849  * | 0.715  * | 0.630 | 0.882  * | 0.569 | 0.904  * | 0.881  * | 0.831  * | 0.856  * |
| Internode length (cm) | 0.803  * | 0.969  * | 0.972  * | 0.794  * | 0.976  * | 0.969  * | 0.788  * | 0.871  * | 0.951  * | 0.819  * | 0.981  * | 0.976  * | 0.861  * | 0.993  * |
| Stem biomass (g) | 0.916  * | 0.971  * | 0.950  * | 0.926  * | 0.971  * | 0.971  * | 0.655  * | 0.800  * | 0.902  * | 0.650  * | 0.966  * | 0.946  * | 0.954  * | 0.980  * |
| Leaf biomass (g) | 0.944  * | 0.977  * | 0.937  * | 0.946  * | 0.969  * | 0.988  * | 0.680  * | 0.781  * | 0.935  * | 0.577  * | 0.970  * | 0.963  * | 0.975  * | 0.995  * |
| Leaf sheath biomass (g) | 0.907  * | 0.983  * | 0.968  * | 0.894  * | 0.973  * | 0.970  * | 0.669  * | 0.836  * | 0.932  * | 0.652  * | 0.953  * | 0.947  * | 0.929  * | 0.983  * |
| Total biomass (g) | 0.932  * | 0.973  * | 0.948  * | 0.937  * | 0.971  * | 0.981  * | 0.660  * | 0.794  * | 0.915  * | 0.608  * | 0.967  * | 0.952  * | 0.964  * | 0.988  * |
| Stem fraction (%) | 0.401 | 0.979  * | 0.963  * | 0.386 | 0.917  * | 0.823  * | 0.888  * | 0.981  * | 0.942  * | 0.940  * | 0.954  * | 0.966  * | 0.500 | 0.942  * |
| Leaf fraction (%) | 0.362 | 0.841  * | 0.852  * | 0.355 | 0.757  * | 0.663  * | 0.755  * | 0.919  * | 0.806  * | 0.818  * | 0.829  * | 0.851  * | 0.397 | 0.790  * |
| Leaf sheath fraction (%) | 0.767  * | 0.869  * | 0.808  * | 0.836  * | 0.879  * | 0.890  * | 0.756  * | 0.653  * | 0.842  * | 0.559  * | 0.918  * | 0.924  * | 0.913  * | 0.902  * |

**TABLE S17** Significant correlations between the characteristics of *Phragmites australis* at the sampling sites in heterogeneous habitats (H1, 2, and 3) and estimates of their genetic (A) and epigenetic diversity (B).

| Variable y | Variable x | Equation | R^2^ | *P* value |
| --- | --- | --- | --- | --- |
| (A) |  |  |  |  |
| Node number | He^1^ | y=6.452+28.637x | 0.451 | 0.047 |
| Node number | I^2^ | y=6.559+18.794x | 0.448 | 0.049 |
| (B) |  |  |  |  |
| Height (cm) | (0,1)^3^ | y=693.167-4026.826x | 0.547 | 0.023 |
| Leaf length (cm) | (0,1) | y=94.828-515.674x | 0.499 | 0.034 |
| Node number | (0,1) | y=27.355-93.898x | 0.450 | 0.048 |
| Internode length (cm) | (0,1) | y=39.941-228.784x | 0.549 | 0.022 |
| Stem biomass (g) | (0,1) | y=7.223-43.018x | 0.481 | 0.038 |
| Leaf biomass (g) | (0,1) | y=6.145-35.887x | 0.490 | 0.036 |
| Leaf sheath biomass (g) | (0,1) | y=3.419-19.68x | 0.451 | 0.048 |
| Total biomass (g) | (0,1) | y=17.951-105.829x | 0.482 | 0.038 |
| Leaf sheath fraction (%) | (0,1) | y=-0.360+3.948x | 0.588 | 0.016 |

Note: ^1^ He, Nei’s gene diversity; ^2^I, Shannon's information index; ^3^the mean epiallelic frequency of (0,1) methylation-susceptible polymorphic loci detected by *Hpa*Ⅱ and *Msp*Ⅰ.

**
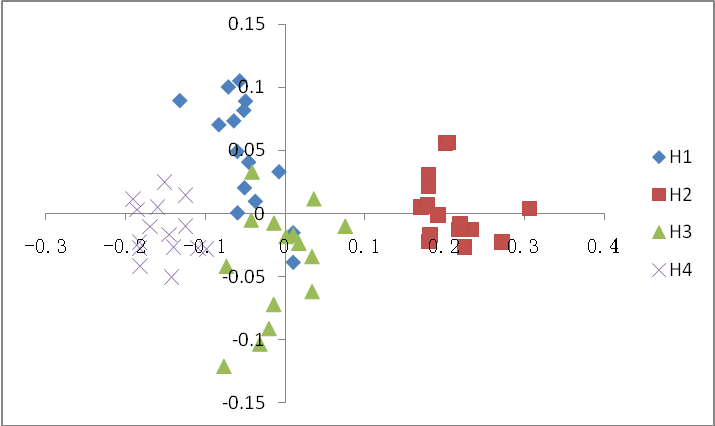
**

**FIGURE S1** Principal coordinate diagram for the soil characteristics of the 4 habitats in the field survey experiment

Note: soil characteristics include soil moisture, pH, electrical conductivity (EC), and the contents of total P and N, organic matter, NO^3^-N, NH^4^-N, Cl^-^, SO_4_^2-^, Na^+^, K^+^, Mg^2+^, and Ca^2+^


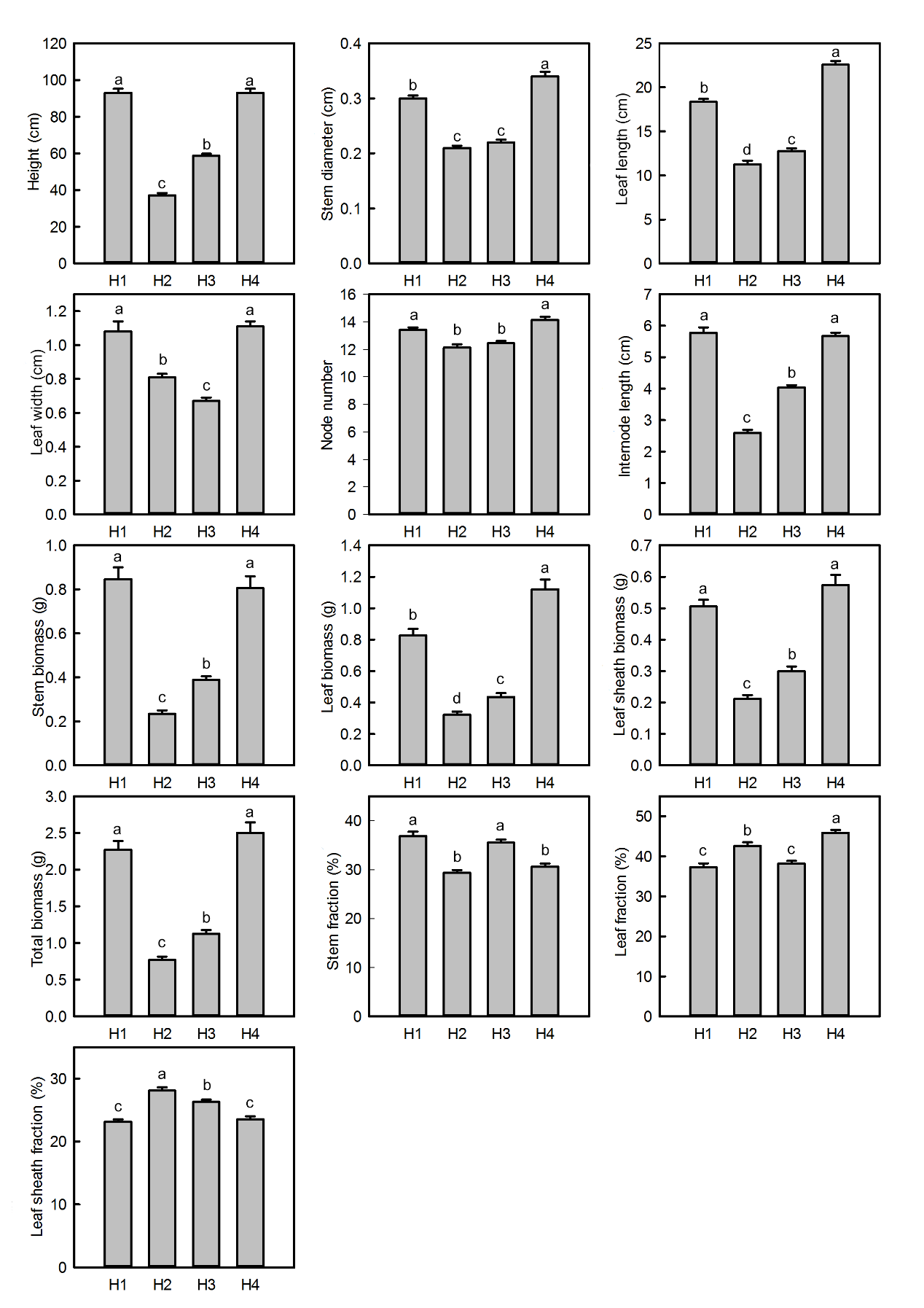


**FIGURE S2** Comparison of the characteristics of *Phragmites australis* in four different habitats in the field survey (M±SE)

Note: Different letters indicate significant differences between habitats at the 5% level.

**
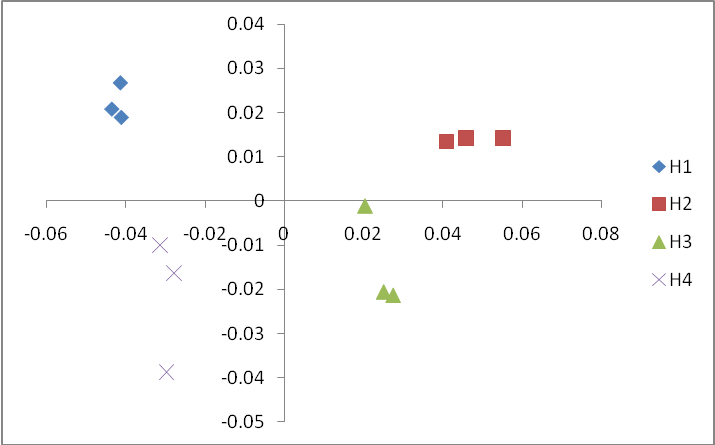
**

**FIGURE S3** Principal coordinate diagram for plant characteristics of *Phragmites australis* in 4 habitats in the field survey experiment

Note: plant characteristics include height, stem diameter, leaf length and width, node number, internode length, stem biomass, leaf biomass, leaf sheath biomass, total biomass, and the stem, leaf and leaf sheath fractions. Each point corresponds to the mean of the characteristic at each site.


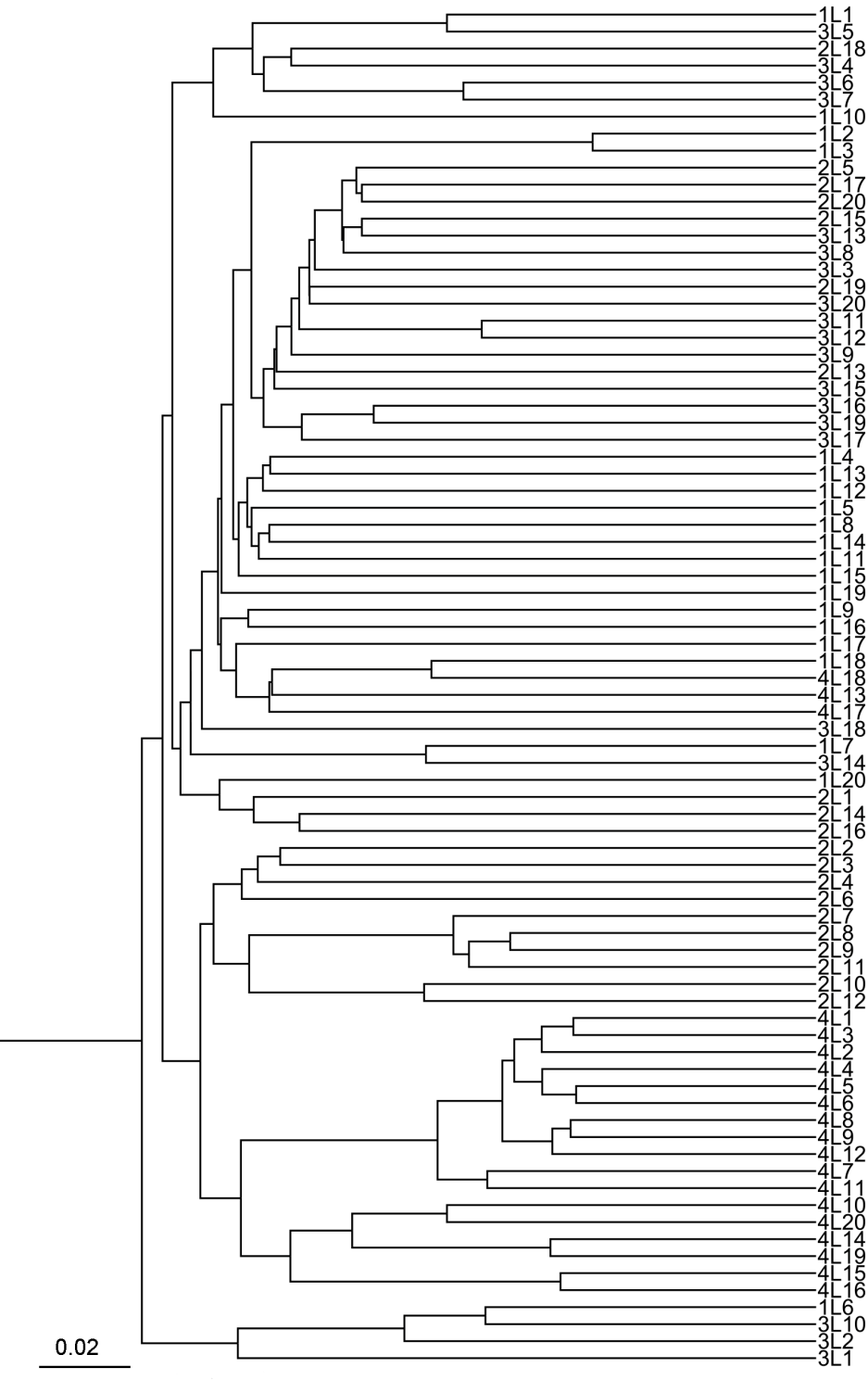


**FIGURE S4** Dendrogram of 96 clones of *Phragmites australis* in the four habitats at small scales in the Songnen Steppe constructed by the UPGMA method.

Note: 1L, 2L, 3L and 4L indicate the four habitats.


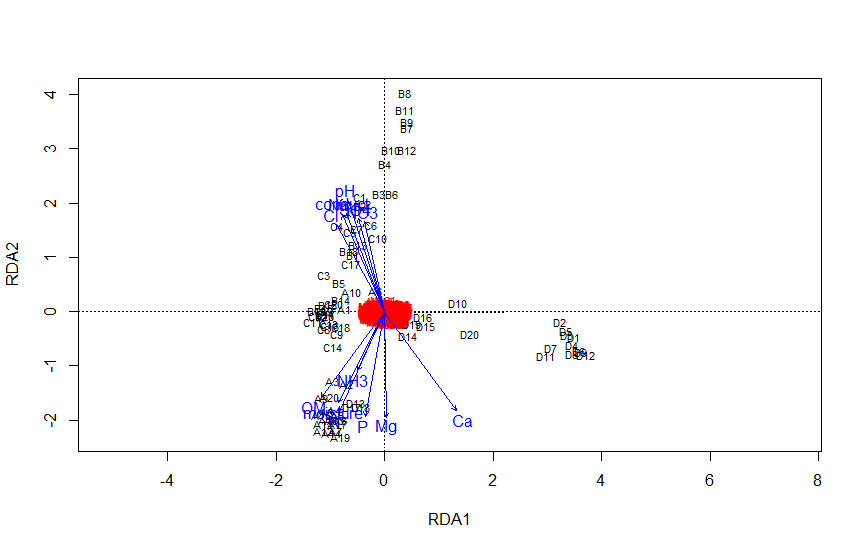


**FIGURE S5** Projection of AFLPs and environmental variables in the RDA. The first two axes of the RDA projection represent 7.35% (RDA1) and 3.41% (RDA2) of the explained variance, with A, B, C, and D representing habitat1, 2, 3, 4 and 1-20 representing 20 individuals correspondingly.


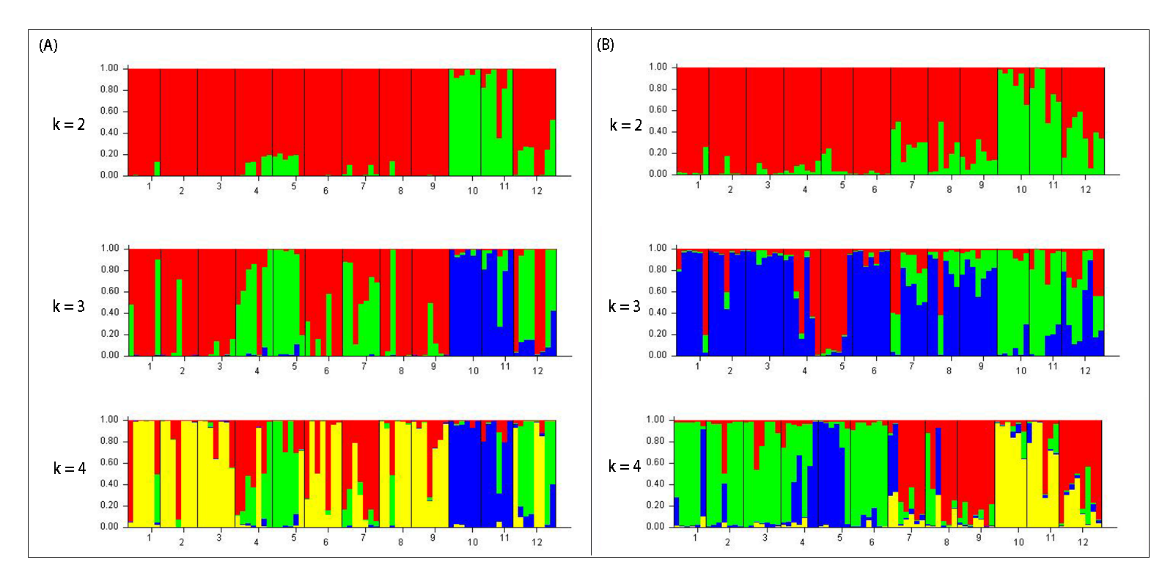


**FIGURE S6** Genetic and epigenetic divergence among four habitats of reed illustrated by STRUCTRUE2.3.1 based on AFLPs (a) and MSAPs (b) (k=2-4). We identified K=3 for AFLPs and K=4 for MSAPs as the most probable number of clusters according to ΔK. Each individual is represented by a single vertical line broken into k colored segments, with lengths proportional to each of the K inferred clusters. Populations were identified at the bottom of the histogram, with 1,2,3 representing habitat 1 distributed in the western, middle, and eastern areas, likewise, 4,5,6 representing habitat 2, 7,8,9 representing habitat 3 and 10,11,12 representing habitat 4.


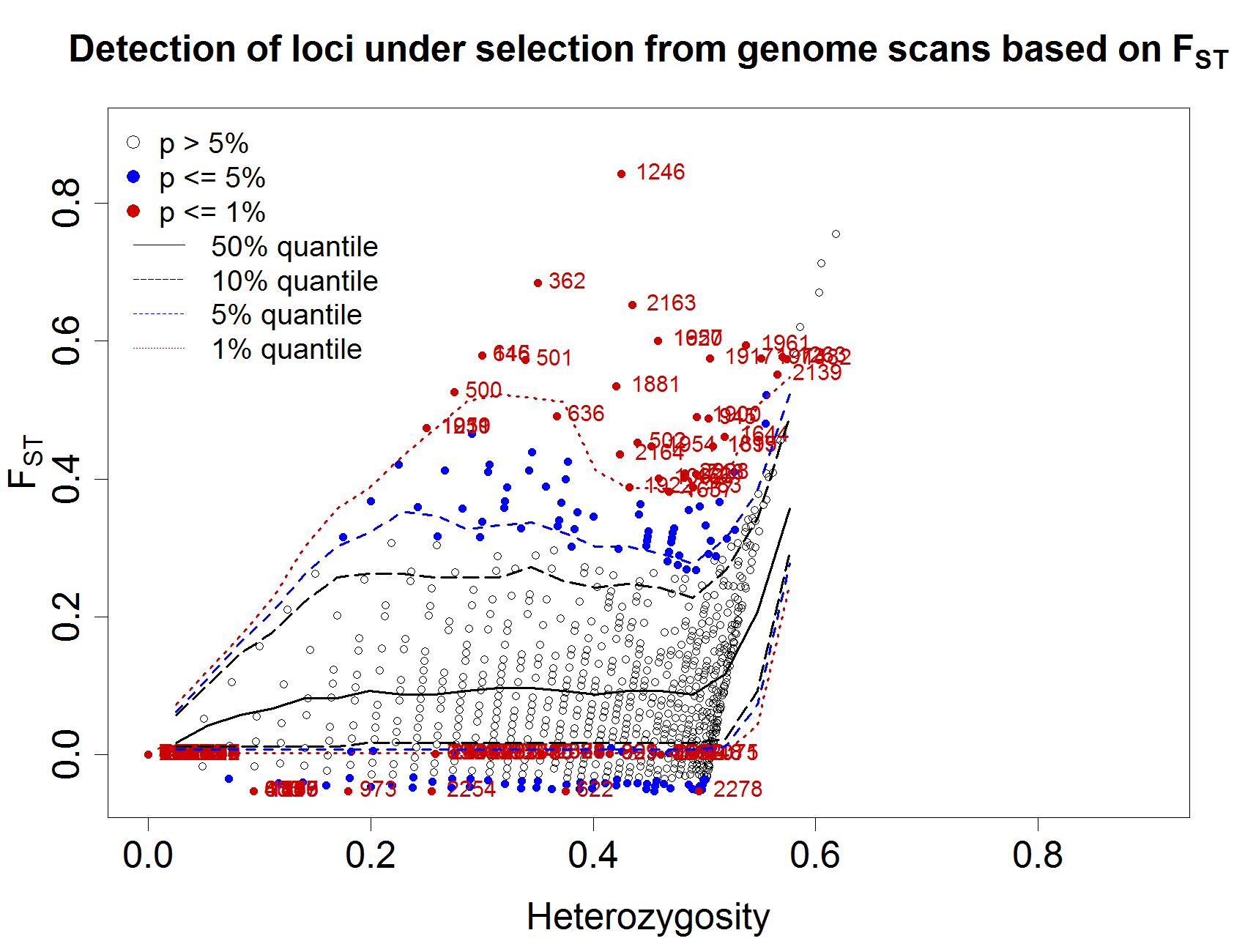
 **FIGURE S7** Detection of loci under selection in *Phragmites australis* from 4 habitats obtained with Arlequin 3.5

Note: A plot of the joint distribution of Fst values and heterozygosity generated is shown. Loci significant at the 5% and 1% levels are shown as blue and red filled circles, respectively, whereas the numbers of loci under selection at the 1% level are indicated. Some outliers presented a lower F_ST_ value than expected under neutrality, which suggests that they have potentially undergone balancing selection. The dots above the uppermost line are classified as outliers potentially under directional selection, which are indicated in Table S10 with 35, 24 outliers showing higher F_ST_ values than expected under neutrality at the 99%,99.5% probability level, respectively.


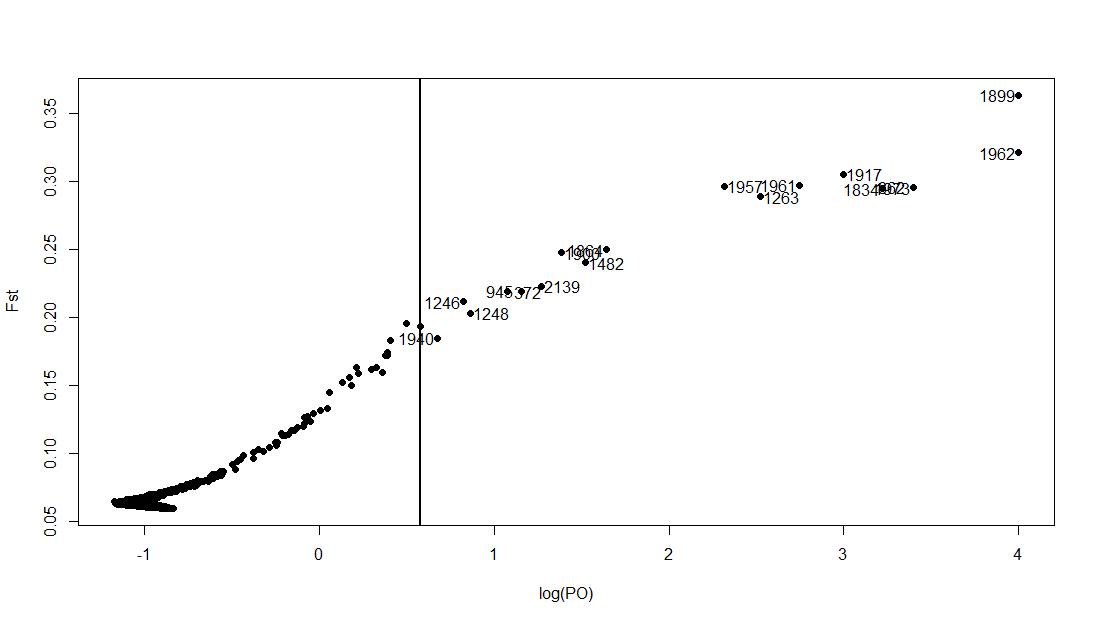


**FIGURE S8** Detection of loci under selection in *Phragmites australis* from 4 habitats obtained with BayeScan 2.0

Note: F_ST_ is plotted against the log10 posterior odds (PO). Each dot corresponds to an AFLP locus. The vertical line shows the critical PO to identify outlier markers. The 18 points on the right side of the vertical line are interpreted as having been affected by selection, which are indicated in Table S11.


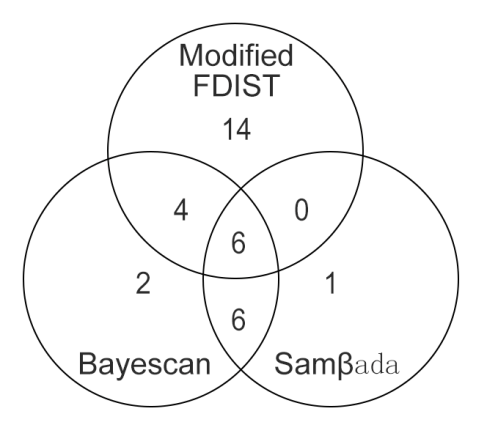


**FIGURE S9** Venn diagram illustrating the overlap of outliers detected with three different methods.


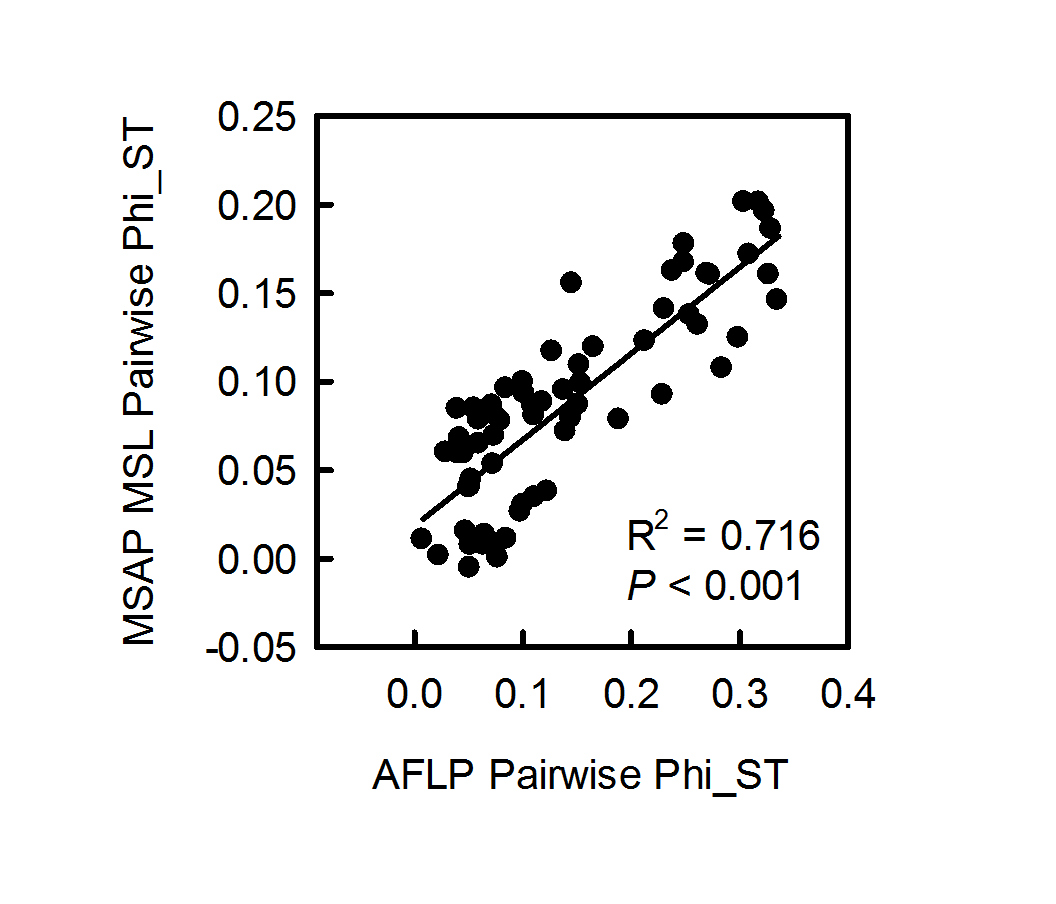


**FIGURE S10** Significant relationships between AFLP pairwise Phi_ST and pairwise Phi_ST of MSAP MSL of reed populations


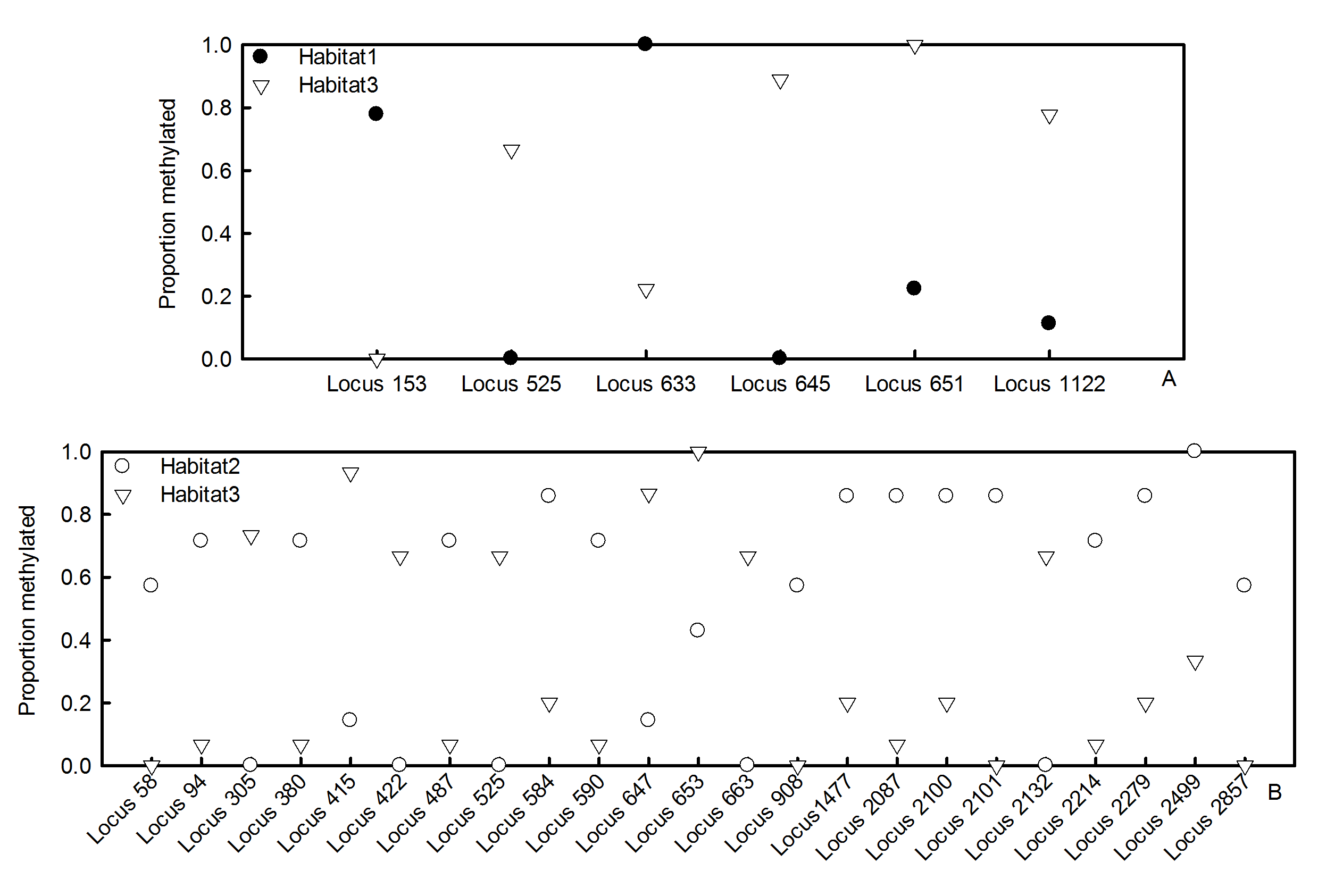


**FIGURE S11** The proportion of *P. australis* individuals with methylation at MSAP loci that showed significant epigenetic differentiation between habitats 1 and 3 (A) and habitats 2 and 3 (B).
